# Molecular architecture of *Streptococcus pneumoniae* surface thioredoxin-fold lipoproteins crucial for extracellular oxidative stress resistance and maintenance of virulence

**DOI:** 10.1002/emmm.201202435

**Published:** 2013-10-18

**Authors:** Malek Saleh, Sergio G Bartual, Mohammed R Abdullah, Inga Jensch, Tauseef M Asmat, Lothar Petruschka, Thomas Pribyl, Manuela Gellert, Christopher H Lillig, Haike Antelmann, Juan A Hermoso, Sven Hammerschmidt

**Affiliations:** 1 Department Genetics of Microorganisms, Interfaculty Institute for Genetics and Functional Genomics, Ernst Moritz Arndt University of GreifswaldGreifswald, Germany; 2 Departamento de Cristalografía y Biología Estructural, Instituto de Química-Física “Rocasolano”CSIC, Madrid, Spain; 3 Institute for Medical Biochemistry and Molecular Biology, University Medicine, Ernst Moritz Arndt University of GreifswaldGreifswald, Germany; 4 Institute for Microbiology, Ernst Moritz Arndt University of GreifswaldGreifswald, Germany

**Keywords:** lipoproteins, meningitis, oxidative stress, pneumococci, pneumonia

## Abstract

The respiratory pathogen *Streptococcus pneumoniae* has evolved efficient mechanisms to resist oxidative stress conditions and to displace other bacteria in the nasopharynx. Here we characterize at physiological, functional and structural levels two novel surface-exposed thioredoxin-family lipoproteins, Etrx1 and Etrx2. The impact of both Etrx proteins and their redox partner methionine sulfoxide reductase *Sp*MsrAB2 on pneumococcal pathogenesis was assessed in mouse virulence studies and phagocytosis assays. The results demonstrate that loss of function of either both Etrx proteins or *Sp*MsrAB2 dramatically attenuated pneumococcal virulence in the acute mouse pneumonia model and that Etrx proteins compensate each other. The deficiency of Etrx proteins or *Sp*MsrAB2 further enhanced bacterial uptake by macrophages, and accelerated pneumococcal killing by H_2_O_2_ or free methionine sulfoxides (MetSO). Moreover, the absence of both Etrx redox pathways provokes an accumulation of oxidized *Sp*MsrAB2 *in vivo*. Taken together our results reveal insights into the role of two extracellular electron pathways required for reduction of *Sp*MsrAB2 and surface-exposed MetSO. Identification of this system and its target proteins paves the way for the design of novel antimicrobials.

## INTRODUCTION

*Streptococcus pneumoniae* (pneumococci) are Gram-positive human commensals but also pathogens with high virulence potential. Pneumococci are dreaded as the etiologic agent of respiratory and life-threatening invasive diseases, such as pneumonia, meningitis and septicemia. The disease burden is high in developed and developing countries and, *e.g*. one million children under the age of 5 years are killed every year (Gamez & Hammerschmidt, 2012; Kadioglu et al, 2008). Its location in the human respiratory tract forces the bacterial cell to develop mechanisms to resist the host defenses like the oxidative burst produced by the innate immune system (West et al, 2011).

Reactive oxygen species (ROS) are also produced during one-electron transfer reactions to O_2_ that include hydrogen peroxide (H_2_O_2_), hydroxyl radical (HO^•^) and superoxide anions (O_2_^−^). ROS are able to react with all cellular macromolecules, such as proteins, lipids, carbohydrates and nucleotides (Gutteridge & Halliwell, 2000; Johansson et al, 2004). Cysteine and methionine (Met) residues are the most susceptible targets for oxidation by ROS. The oxidation of Met residues in proteins generates methionine sulfoxides (MetSO) that can induce conformational changes leading either to activation or inactivation of proteins (Zeller & Klug, 2006). In eukaryotic cells, Met oxidation has been shown to inactivate calmodulin and activate calmodulin kinase (Bigelow & Squier, 2011; Erickson et al, 2008) or in *Escherichia coli* the transcription factor HypT is activated specifically by MetSO formation (Drazic et al, 2013). This reaction is reversible and MetSO modifications can be reduced to Met by methionine sulfoxide reductases (Msr). Depending on the stereospecific orientation two distinct classes of Msr enzymes have been described. MsrA is specific for the reduction of the S-form and MsrB reduces the R-form of MetSO (Brot et al, 1981; Grimaud et al, 2001; Sharov & Schoneich, 2000; Weissbach et al, 2005). MsrA and MsrB occur typically as separate enzymes, but in some bacteria like *S. pneumoniae*, *Neisseria gonorrhoeae* and *Haemophilus influenzae* they are translationally fused as MsrAB (Delaye et al, 2007; Kim et al, 2009; Tarrago & Gladyshev, 2012; Wizemann et al, 1996). The reduction of MetSO by Msr involves the formation of an intramolecular disulphide in MsrA and MsrB, which is reduced by thioredoxins (Trx) that transfer electrons from their CXXC active site to the MsrA and MsrB disulphides (Hoshi & Heinemann, 2001; Lowther et al, 2000; Ranaivoson et al, 2009). The reduction of MsrA and MsrB by Trx leads to oxidation of Trx, which is reduced by the thioredoxin reductase (TrxB) on expense of NADPH. The Trx/TrxB system together with the glutaredoxin/glutathione (GSH)/GSH reductase system maintain the cellular reducing environment (Fernandes & Holmgren, 2004; Paget & Buttner, 2003). Trx proteins also function as electron donors to regenerate peroxiredoxins and other antioxidant enzymes in their detoxification cycle (Collet & Messens, 2010; Das & Das, 2000; Hanschmann et al, 2013; Kang et al, 1998; Zander et al, 1998).

Pneumococci are able to produce millimolar H_2_O_2_ by the pyruvate oxidase SpxB as a chemical weapon against bacterial competitors and hence this pathogen can tolerate high H_2_O_2_ concentrations (Pericone et al, 2002, 2003). By H_2_O_2_ production *S. pneumoniae* can displace *H. influenzae*, *N. meningitidis* and *Staphylococcus aureus*, which also involves the induction of resident prophages in *S. aureus* (Margolis, 2009; Pericone et al, 2000; Selva et al, 2009).

Despite their peroxide resistance, pneumococci lack the major peroxide scavenging enzyme catalase. However, other ROS scavenging enzymes, like the superoxide dismutase (SodA) (Yesilkaya et al, 2000), NADH oxidase (Auzat et al, 1999) and alkyl hydroperoxidase (Paterson et al, 2006) limit ROS mediated killing in the cytoplasm. In addition, the thiol peroxidase (TpxD) functions in H_2_O_2_ detoxification and survival under aerobic conditions and is responsible for enhanced survival of pneumococci in mice virulence assays after intranasal infections (Hajaj et al, 2012; Hiller et al, 2007; Yesilkaya et al, 2013). Recently, the roles of the cellular redox buffer GSH, its GshT importer and the glutathione reductase (Gor) in protection against oxidative stress and during pneumococcal colonization have been demonstrated (Potter et al, 2012). Such cytoplasmic antioxidant and protein reducing systems are well studied in many bacteria (Kim et al, 2009; Wizemann et al, 1996). In contrast, the extracellular compartment of Gram-positive bacteria and the periplasm of Gram-negatives are considered as an oxidizing environment where disulphide bond oxidases and isomerases, such as the DsbA/DsbB and DsbC/DsbD pathways catalyse oxidative protein folding (Cho & Collet, 2013; Denoncin & Collet, 2013). However, also periplasmic reducing redox pathways have been described in few pathogenic Gram-negative bacteria, such as in *N. gonorrhoeae* and *N. meningitidis* (Brot et al, 2006; Ranaivoson et al, 2006; Wu et al, 2005). In these periplasmic reducing redox pathways of Gram-negative bacteria, the membrane-bound DsbD protein provides the electrons for reduction of sulfenic acids, for cytochrome maturation and to reduce periplasmic antioxidant enzymes, such as peroxidases and Msr (Cho & Collet, 2013; Denoncin & Collet, 2013). In pneumococci, the extracellular thioredoxin-like protein TlpA was suggested to be involved in the extracellular oxidative stress resistance, but DsbD-like reducing redox pathways that are associated with TlpA were not explored (Andisi et al, 2012). In addition, the molecular interplay of the surface-exposed TlpA (renamed Etrx1) with its extracellular paralogue Etrx2 on oxidative stress resistance and virulence are unknown. This study discovers the unique molecular architecture of two surface-exposed thioredoxin-systems, Etrx1 and Etrx2 and their redox partners CcdA1, CcdA2 and *Sp*MsrAB2 as important pneumococcal extracellular reducing systems essential for pneumococcal pathogenesis and oxidative stress resistance. We further provide evidence that both CcdA-Etrx pathways function in *Sp*MsrAB2 reduction *in vivo*. Thus, Etrx1, Etrx2 and *Sp*MsrAB2 are attractive targets for the design of novel anti-infectives to block the initial states of pneumococcal infection.

## RESULTS

### Identification and genetic organization of the *etrx1* and *etrx2* operons in pneumococci

**Figure 1:**
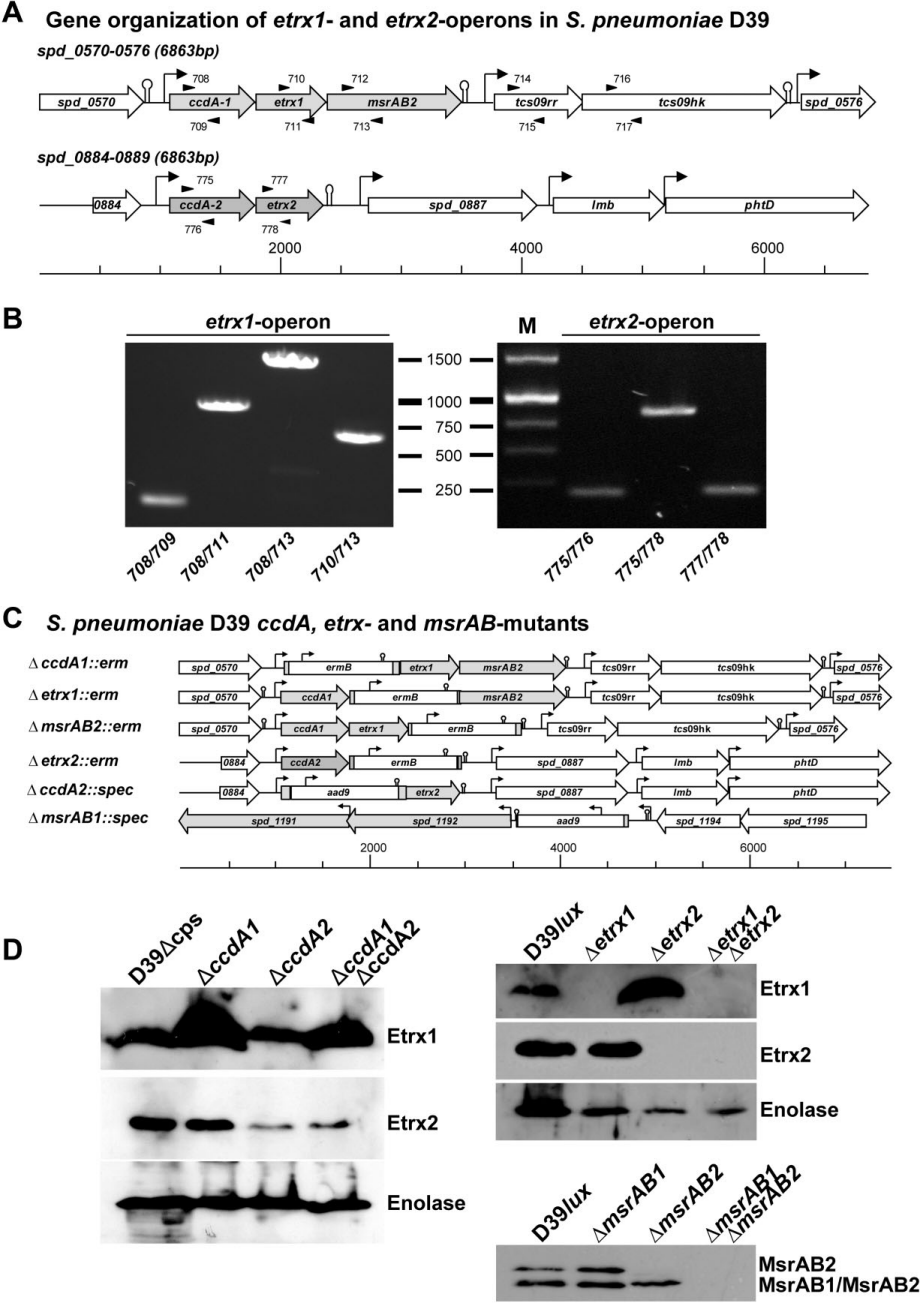
Molecular organization of the *etrx* operons in *Streptococcus pneumoniae* and schematic molecular model of isogenic *etrx*-mutants Source data is available for this figure in the Supporting Information.

The bioinformatic analysis of pneumococcal genomes identified two genes encoding surface-exposed thioredoxin-like lipoproteins, renamed here Etrx1 and Etrx2. Production of the Etrx proteins in *S. pneumoniae* D39 and other strains was demonstrated by immunoblot analysis (Fig 1). Etrx1 consists of 188 amino acids (20.8 kDa) and shows 39.4% sequence identity with Etrx2 (Supporting Information Fig S1) that consists of 185 amino acids (20.7 kDa). The two potential redox partners encoded by the conserved pneumococcal three-gene *etrx1* operon are the methionine sulfoxide reductase AB2 (*Sp*MsrAB2) and cytochrome C-type biogenesis protein, renamed here CcdA1 (Fig 1 and Supporting Information Figs S2–S5). *Sp*MsrAB2 shows 77% of sequence homology with the intracellular pneumococcal *Sp*MsrAB1 (Kim et al, 2009; Supporting Information Fig S6). The *etrx2* gene is located together with a second *ccdA*-like gene (renamed *ccdA2*) in a bicistronic operon, but without a second *msrAB-*like gene (Fig 1A and B and Supporting Information Figs S7 and S8). CcdA1 and CcdA2 share 58.8% sequence identity (Supporting Information Fig S9) and possess 18 and 21% sequence identity, respectively, with the transmembrane domain of the *Neisseria* periplasmic DsbD protein (Krupp et al, 2001; Supporting Information Fig S10).

1. Genetic organization of operons encoding the *etrx1* and *etrx2* genes in *S. pneumoniae* D39. Putative promoters (arrows) were predicted by the Neural Network Promoter Prediction program (http://www.fruitfly.org/seq_tools/promoter.html) and potential rho-independent termination sequences were extracted from the TransTermHP Terminator Prediction list of *S. pneumoniae* D39 (http://transterm.cbcb.umd.edu/tt/Streptococcus_pneumoniae_D39.tt). Black arrowheads indicate primers used for RT-PCR analysis.
2. Transcript length analysis of the putative *etrx1* and *etrx2* operons by RT-PCR. PCR-fragments, generated from cDNA of strain *S. pneumoniae* D39 using the indicated primer combinations, are shown.
3. Schematic models of constructed mutants in the *etrx1*-, et*rx2*-operon and the *msrAB1* gene. Shown are the genetic organization of the operons and insertion sites of the *ermB*- or *aad9*-gene cassette in *ccdA*, *etrx* or *msrAB* genes.
4. Immunoblot analysis of Etrx and MsrAB production in wild-type and mutant pneumococci. Left panel and upper right panel: production of Etrx1 and Etrx2 in *S. pneumoniae* D39 and its isogenic *ccdA1-*, *ccdA2-*, *etrx1-* and *etrx2-*mutants using Etrx specific polyclonal antibodies. Enolase was used as a control and detected with anti-enolase antibodies (Hermans et al, 2006). Lower right panel: production of MsrAB2 in pneumococcal *msrAB1-* and *msrAB2-*mutants using MsrAB2 specific polyclonal antibodies recognizing also the intracellular MsrAB1.

### Etrx1, Etrx2 and *Sp*MsrAB2 are displayed on the pneumococcal surface and are essential for resistance against oxidative stress

**Figure 2:**
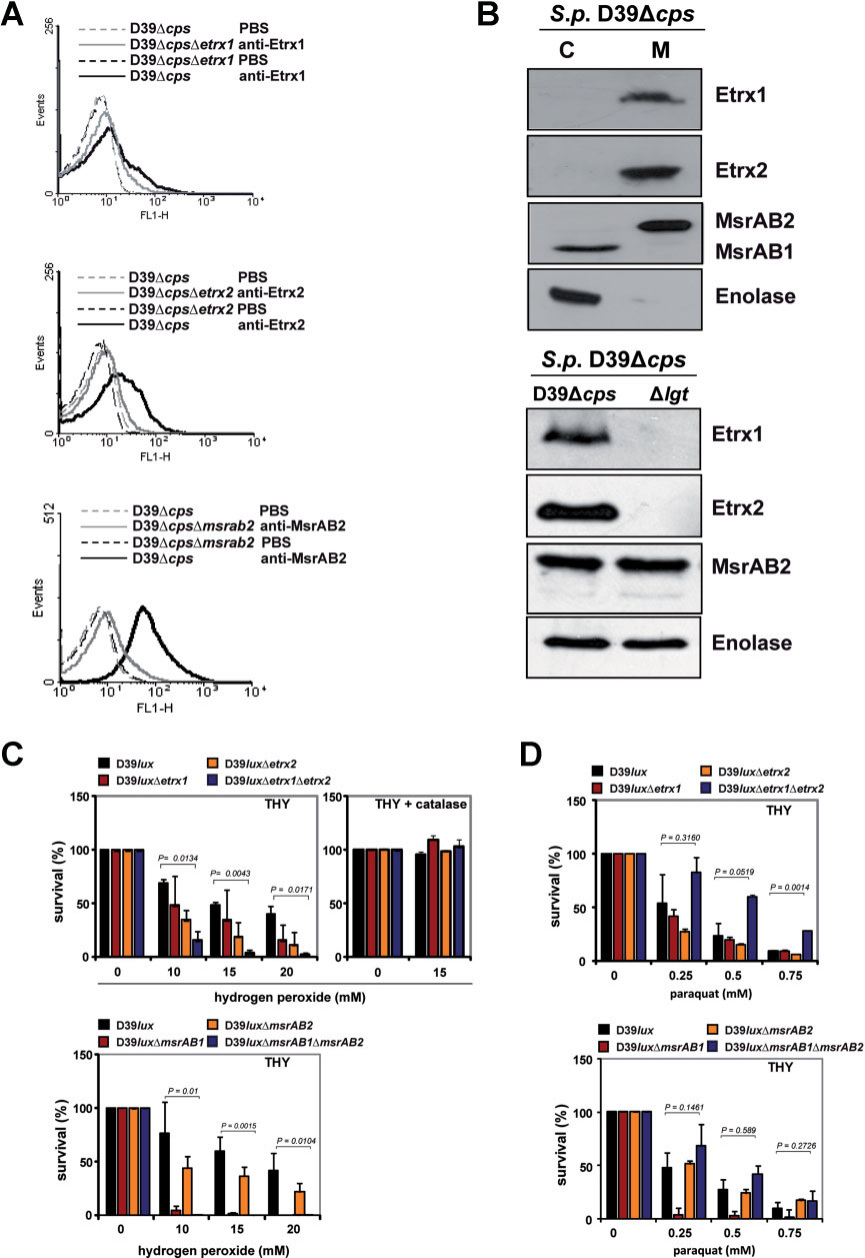
Etrx proteins are displayed on the pneumococcal surface and essential for oxidative stress resistance Source data is available for this figure in the Supporting Information.

Both Etrx proteins possess a typical lipoprotein-specific signal peptide containing a lipobox suggesting covalent binding of the putative lipoproteins to the outer leaflet of the phospholipid bilayer. The anchoring of lipoproteins is catalysed by a diacylglyceryl transferase (Lgt), which adds a diacylglyceryl residue to the thiol group of the conserved cysteine in the lipobox, while the signal peptide is cleaved after translocation and lipid-modification by the lipoprotein-specific signal peptidase Lsp. The lipid-modified cysteine residue remains at the mature lipoprotein as the new N-terminus (Kovacs-Simon et al, 2011). *Sp*MsrAB2 contains one transmembrane segment and is probably anchored via this segment to the pneumococcal membrane. To analyse the surface localization of the putative lipoproteins Etrx1 and Etrx2 and of the membrane anchored protein *Sp*MsrAB2, isogenic mutants D39Δ*etrx1*, D39Δ*etrx2* and D39Δ*msrAB2* were constructed by allelic replacement and verified by immunoblot analysis (Fig 1C and D). Flow cytometric analysis of wild-type and mutants demonstrated that Etrx1, Etrx2, and also *Sp*MsrAB2 are displayed on the pneumococcal surface of wild-type bacteria, while no surface association of Etrx1, Etrx2 and *Sp*MsrAB2 was found in the Δ*etrx1*, Δ*etrx2* and Δ*msrAB2* mutants, respectively (Fig 2A). Proteolytic treatment of pneumococci with trypsin and pronase E, respectively, followed by immunoblot analysis confirmed these results (Supporting Information Fig S11). Immunoblot analysis was further conducted for the cytoplasmic and surface-displayed protein fractions of the wild-type and an *lgt*-mutant that is unable to anchor lipoproteins in the membrane (Voss et al, 2013). This subcellular analysis indicated that Etrx1 and Etrx2 are indeed lipoproteins and surface-exposed. They are released into the medium fraction of the *lgt*-mutant because of their inefficient anchoring to the membrane (Fig 2B). *Sp*MsrAB2 is also displayed on the cell surface but has no lipid-anchor characteristic for lipoproteins and thus, is retained in the *lgt*-mutant membrane fraction (Fig 2B). However, we noticed that the *Sp*MsrAB2 antibodies cross-reacted with the intracellular MsrAB1 that was detected in the cytoplasm of pneumococci (Figs 1D and 2B). Interestingly, the amount of *Sp*MsrAB2 was enhanced in the *ccdA1*- and *etrx1*-mutants, which was not caused by the strategy of the mutant construction (Fig 1C and Supporting Information Fig S12).

A. The surface localization of Etrx1, Etrx2 and MsrAB2 was analysed by flow cytometry. Wild-type pneumococci and isogenic mutants were incubated with anti-Etrx antibodies or PBS followed by detection using a goat anti-mouse IgG coupled Alexa-Fluor-488. The increase of fluorescence intensity (FL1-H) in the histograms indicates the presence of the Etrx and MsrAB2 proteins on the surface of non-encapsulated D39 bacteria, while the mutants and control treated pneumococci do not show an increase in fluorescence intensity.
B. Immunoblot analysis showing the surface localization of Etrx proteins and MsrAB2. Upper panel: pneumococci were fractionated and cytoplasmic (C) and cell membrane (M) fractions were separated by SDS–PAGE. Anti-Etrx or anti-MsrAB polyclonal antibodies were used to detect the proteins. Enolase was used as loading control and detected with anti-enolase antibodies (Hermans et al, 2006). Lower panel: Etrx1 and Etrx2 are lipoproteins as indicated by the absence of the protein in the isogenic *lgt*-mutant of D39Δ*cps* (Δ*lgt*) (Kovacs-Simon et al, 2011; Voss et al, 2013), while *Sp*MsrAB2 is anchored in the membrane via one transmembrane domain (see also Fig 8 and Supporting Information S6).
C,D. Oxidative stress response. Encapsulated bioluminescent D39 and isogenic pneumococcal *etrx*-mutants were treated for 30 min with various concentrations of H_2_O_2_ (C) or 90 min with paraquat (D). Survival was determined by plating and determination of the CFU. Data are represented as mean ± SEM of three independent experiments.

To test the effect of Etrx lipoproteins and *Sp*MsrAB2 on resistance against oxidative stress, D39 and its isogenic *etrx*-mutants were exposed for 30 min to 10, 15 and 20 mM H_2_O_2_ or for 90 min to 0.25, 0.5 and 0.75 mM paraquat (Fig 2C and D). The exposure to 10–20 mM H_2_O_2_ reduced the survival of the wild-type to about 50%. However, the *etrx1*/*etrx2* double mutant was more affected and showed only 20–30% survival after exposure to 10 mM H_2_O_2_, and less than 2% survival in the presence of 20 mM H_2_O_2_. The survival of the *etrx1* or *etrx2* single mutants was also decreased by H_2_O_2_, but to a lower degree as determined for the double mutant (Fig 2C). Similar to the *etrx*-mutants, the survival of the *msrAB2*-mutant was affected by H_2_O_2_. Remarkably, the *msrAB1*-mutant was much more sensitive to H_2_O_2_ compared to the *msrAB2* single or *etrx* double mutants (Fig 2C). This peroxide sensitive phenotype of the *msrAB2*-mutant is in agreement with a previous study (Andisi et al, 2012). The superoxide-generating compound paraquat showed a weaker effect on the survival of the *etrx* single mutants (Fig 2D). Surprisingly, the *etrx* double and *msrAB2* mutants were resistant to 0.25–0.5 mM paraquat, but the reasons are not known. The most dramatic effect on survival was determined for the *msrAB1*-mutant suggesting that *Sp*MsrAB1 is more important for paraquat resistance than *Sp*MsrAB2. These results suggest that both Etrx1 and Etrx2 can function as redox partners for *Sp*MsrAB2 since the inactivation of both Etrx proteins or *Sp*MsrAB2 renders pneumococci, to varying degrees, sensitive to extracellular peroxide stress.

### Crystal structures of Etrx1 and Etrx2

**Figure 3:**
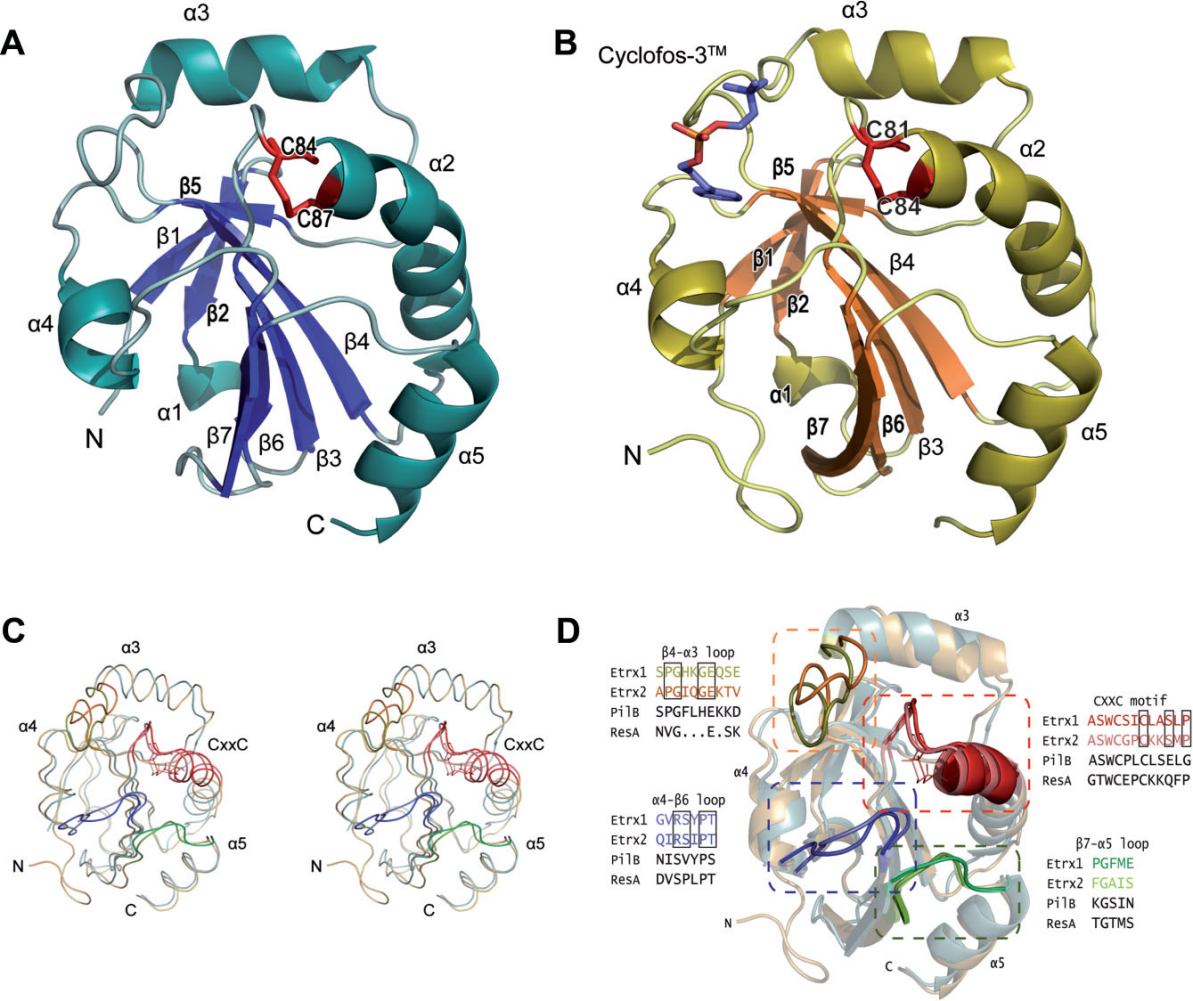
Three-dimensional structures of pneumococcal surface-exposed thioredoxins

The crystal structures of oxidized Etrx1 and Etrx2 have been solved in this study using the untagged-recombinant lipoproteins (Supporting Information Fig S13; details of the expression cloning and protein purification are described in the Supporting Information). Crystallographic data collection and model statistics are summarized in Table1. The Etrx1 model comprises amino acid residues from Ala53 to Leu187. Etrx1 presents a globular structure (38 × 28 × 29 Å) displaying a thioredoxin-like fold that contains seven β-strands and five α-helices (Fig 3A). Besides the canonical Trx fold, Etrx1 has two insertions (Supporting Information Fig S14). The first insertion (residues 53–72) results in β1, β2 and α1 elements, and the second insertion (residues 112–144) gives rise to an additional β-strand (β5) and α-helix (α3). The loop connecting β3 with α2 contains the CXXC motif (^84^CSIC^87^) defining the nucleophilic active site Cys84 and the resolving Cys87 (Fig 3D). These cysteine residues form a disulphide bridge reflecting the oxidized state of the protein. The structural data clearly show that only the active site Cys84 is solvent-exposed and accessible for electron transfer reactions. At the beginning of the second insertion and near the active site there is an additional loop connecting β4 with α3 (residues 112–120) that is not present in the closely related family of cytochrome maturation proteins. A search for proteins structurally related to Etrx1 performed with DALI server (Holm & Rosenstrom, 2010) identified the N-terminal domain of PilB protein (NterPilB) from *N. gonorrhoeae* (PDB code 2H30, *Z* score of 21.1 and rmsd of 1.5 Å for 133 Cα atoms), and *N. meningitidis* (PDB code 2FY6, *Z* score of 21.1 and rmsd of 2.1 Å for 136 Cα atoms) as the most closely related 3D structures. PilB is secreted to the periplasm and involved in the pathogen survival strategies against the oxidative burst as encountered in the host (Quinternet et al, 2008).

**Table 1:** Data collection and refinement statistics

Data collection	Etrx1	Etrx2:Cyclofos 3™	Etrx2:HED
Space group	*P*4_3_2_1_2	*P*2_1_2_1_2_1_	*P*1
*a*, *b*, *c* (Å)	62.85, 62.85, 89.60	61.46, 116.31, 116,42	32.10, 36.08, 58.64
*α*, *β*, *γ* (°)	90, 90, 90	90, 90, 90	101.13, 100.26, 101.59
*T* (K)	100	100	100
X-ray source	Synchrotron	Synchrotron	Synchrotron
Wavelength (Å)	1.0053	0.93340	1.00000
Resolution (Å)	29.87–1.48 (1.56–1.48)	32.81–1.77 (1.86–1.77)	23.89–1.22 (1.24–1.22)
Total no. reflections	810807 (118971)	467968 (66226)	243076 (69159)
No. unique reflections	30641(4393)	82391 (11813)	67595 (3219)
Redundancy	26.5 (27.1)	5.7 (5.6)	3.2 (3.2)
Completeness (%)	99.9 (100)	99.9 (99.4)	92.1 (89.0)
Average *I/σ*(*I*)	24.2 (7.1)	37.1 (4.2)	13.3 (5.5)
*R*_merge_	0.088 (0.51)	0.041 (0.48)	0.043 (0.16)
Refinement statistics			
Resolution (Å)	28.11 (1.48)	32.81 (1.77)	23.87 (1.22)
*R*_work_*/R*_free_	0.18/0.20	0.16/0.19	0.15/0.18
Non-hydrogen atoms	1435	5649	2957
Protein	1173	4990	2531
Ligands	39	236	23
Solvent	223	423	403
B-factor values (Å)^2^			
Protein	17.80	18.71	16.88
Ligands	33.48	64.53	44.43
Solvent	31.39	34.14	28.43
Rmsd bond length (Å)	0.058	0.012	0.009
Rmsd bond angles (°)	1.373	1.302	1.256
PDB code	4HQS	2YP6	4HQZ

1. Overall Etrx1 structure showing β-strands as blue arrows and α-helices as light blue helices. Catalytic Cys84 and Cys87 residues are labelled in red.
2. Overall Etrx2 structure representation showing β-strands as orange arrows and α-helices in green. Cyclofos-3™ is shown as sticks. Catalytic Cys81 and Cys84 residues are labelled in red.
3. Stereo view of the superimposition of Etrx1 (cyan) and Etrx2 (light brown). Loops around the active site are highlighted: the CXXC region in red, the β4–α3 loop in yellow (Etrx1) and orange (Etrx2), the α4–β6 loop in blue and the β7–α5 in green.
4. Structural comparison between Etrx1 and Etrx2. Loops around the active site are highlighted: the CXXC region in red, the β4–α3 loop in yellow (Etrx1) and orange (Etrx2), the α4–β6 loop in blue and the β7–α5 in green. Sequence alignment of Etrx1, Etrx2 and equivalent regions in NterPilB and ResA loop regions are shown for comparison. Composition and colours were chosen according to Quinternet et al (2009).

**Figure 4:**
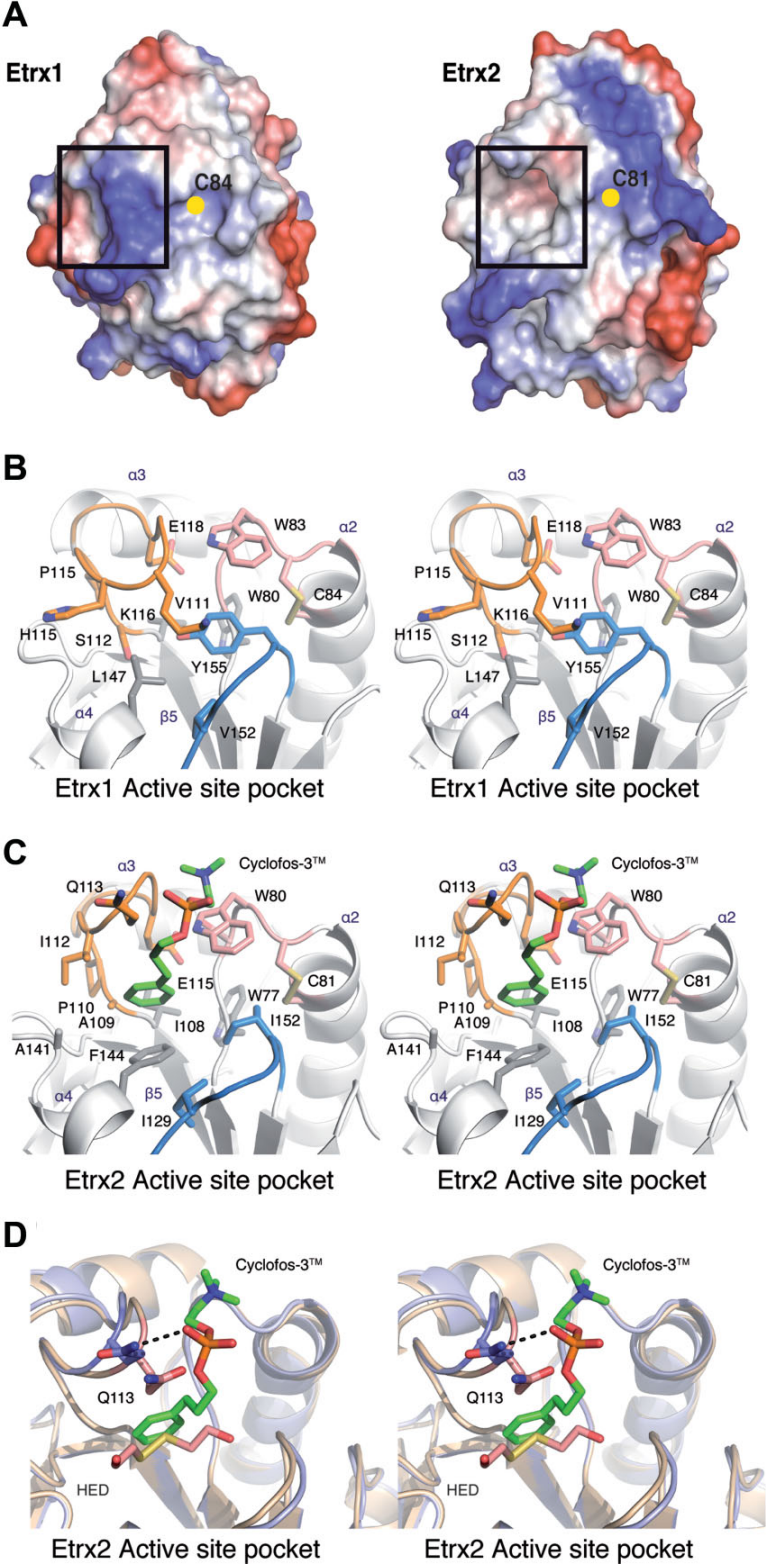
Structural differences between Etrx1 and Etrx2 active sites

The three-dimensional structure of Etrx2 in complex with Cyclofos-3™ comprises the sequence from Ile43 to Asn185. Four molecules were found in the asymmetric unit presenting a similar structure (rmsd value of 0.184 Å for all Cα atoms) (Fig 3B). Etrx2 presents also a modified thioredoxin-like fold that is highly reminiscent of Etrx1 (rmsd value of 1.5 Å for 130 Cα atoms), where the classical thioredoxin-like motif of Etrx2 is embellished by a central αβ insertion and an N-terminal β-hairpin (Fig 3C). The CXXC motif (^81^CGPC^84^) defines the active site (Fig 3D). The main differences between Etrx1 and Etrx2 structures are the presence of eight extra residues forming a coil at the C-terminus of Etrx2, the conformation of β4–α3 loop, the disposition of α3 helix and the more extended conformation of the β7 strand in Etrx2 (Fig 3C and D). ResA, a thiol-disulfide oxidoreductase involved in cytochrome c biosynthesis in *Bacillus subtilis* and accepting electrons from CcdA, is the closest reported homologue of Etrx2 (rmsd of 1.5 Å for 132 Cα atoms and *Z* score of 20.7). The Cyclofos-3™ is located in close proximity to the Etrx2 active site. The ligand, found in the four monomers of the asymmetric unit, is stabilized in a pocket (Fig 4A) formed by the β4–α3 loop, the α4 helix, the α4–β3 loop and the CXXC region (Fig 3B). This pocket presents a strong hydrophobic character (Ala78, Trp80, Ala109, Pro110, Ile112, Ala141, Phe144, Ile149, Ile 122) *versus* the more polar character of the equivalent region in Etrx1 (Fig 4A–C). Cyclofos-3™ is stabilized by an H-bond with Gln113, by cation–π interaction of the polar face of the detergent with Trp80 and by pi–pi stacking interaction between phenyl ring of the detergent and Phe144 (Fig 4C). Attempts to obtain the reduced form of Etrx2 by co-crystallization with β-mercaptoethanol resulted in a new oxidized structure in which Etrx2 forms a complex with a 2-hydroxyethyl disulfide (HED) molecule (Fig 4D). A different conformation of the β4–α3 loop was observed coupled with the presence of the HED molecule at the hydrophobic cavity.

1. Electrostatic potential on the Etrx1 and Etrx2 molecular surface. The exposed active sites are marked with a black box. Catalytic cysteines are labelled. Acidic regions are coloured in red and basic regions in blue.
2. Stereo view of Etrx1 active site. Colour code as in Fig 3C.
3. Stereo view of Etrx2 active site. Relevant residues and Cyclofos-3™ ligand are drawn as capped sticks. Carbon atoms of the ligand are shown in green, while those in the protein are colour coded as in Fig 3C. Hydrogen bonds are shown as dashed lines.
4. Stereo view of the Etrx2 catalytic core in complex with ligands. Shown is the superimposition of the reduced (light blue) and oxidized (light brown) forms. Relevant residues are labelled and shown as capped sticks. Polar interactions are shown as dashed lines.

Both Etrx proteins share a conserved *cis*-proline residue (Pro156 in Etrx1 and Pro153 in Etrx2), placed in front of the catalytic cysteine residue, which is conserved in all thioredoxin-like proteins. As already mentioned above, the hydrophobic binding site, which is observed for Etrx2 but not for Etrx1, is of special interest. This hydrophobic binding site has not been observed in any extra-cytoplasmic thiol-disulfide oxidoreductases (TDORs) reported so far. Remarkably, both pockets differ in their amino acid composition and also in their conformation. Etrx2 presents a deep groove, while the Etrx1 site is filled by Tyr155 and covered by the α4–β3 loop. The electrostatic potential on the molecular surface is also quite different in both proteins. The Etrx2 binding site presents a hydrophobic character while Etrx1 presents a highly basic character in this region (Fig 4A). These differences point to a differential specificity of each Etrx protein for its redox partner.

### Functional analysis of the extracellular thioredoxin proteins Etrx1 and Etrx2 of *S. pneumoniae*

**Figure 5:**
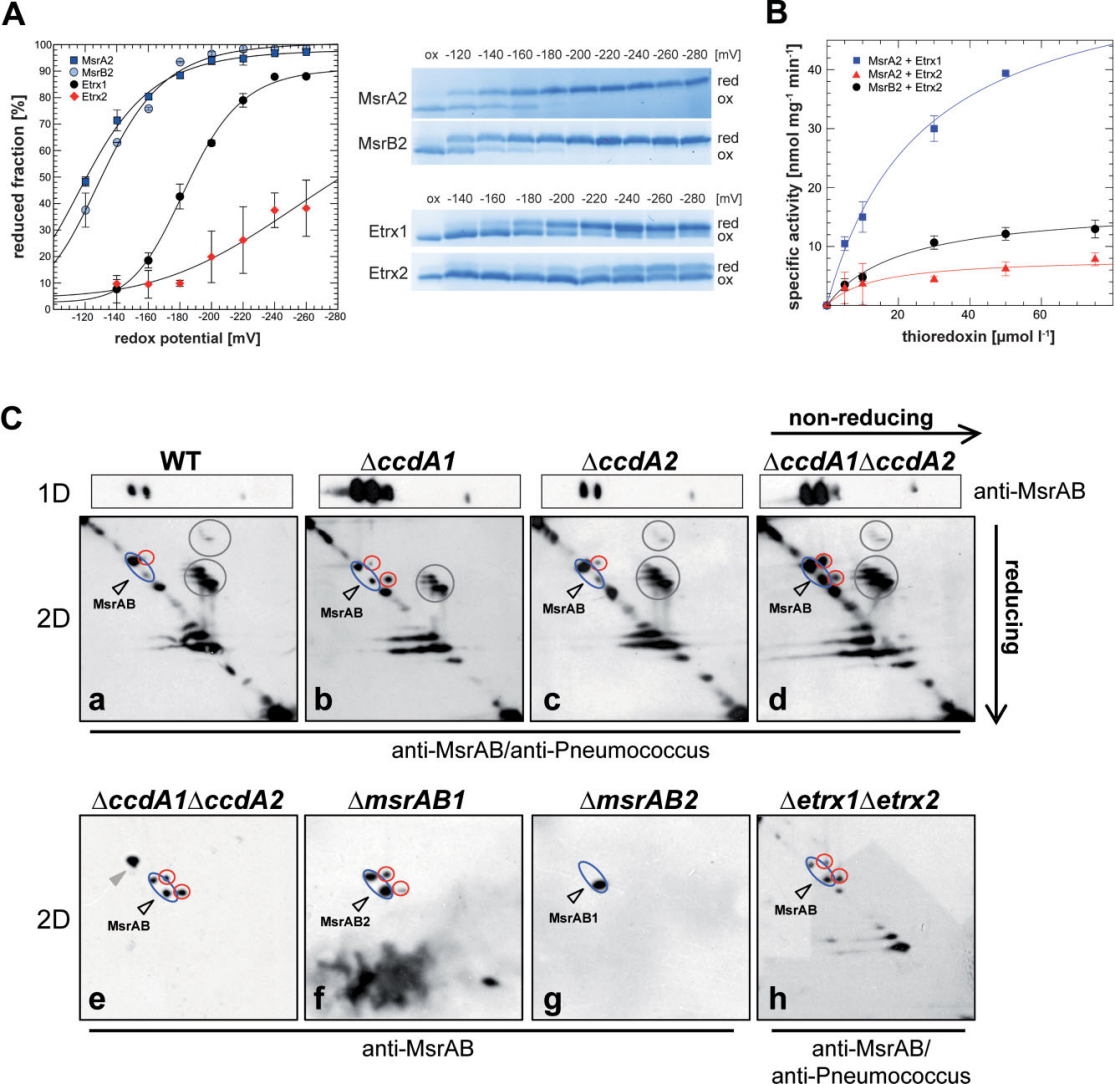
Reduction of SpMsrAB2 by thioredoxin lipoproteins Etrx1 and Etrx2 Source data is available for this figure in the Supporting Information.

To decipher the function of Etrx1 and Etrx2 as thioredoxin proteins reducing the potential redox partner protein *Sp*MsrAB2, the redox states and potentials of purified Etrx proteins and *Sp*MsrAB2 subunits (MsrA2 and MsrB2; Supporting Information Fig S13) were determined. Different ratios of GSH and glutathione disulfide (GSSG) were used to determine the redox potentials. The reduced thiols of Etrx1, Etrx2 MsrA2 and MsrB2 were alkylated with AMS in an anaerobic nitrogen environment, causing a mass shift after separation by SDS–PAGE. The redox potentials were calculated from densitometric analysis and the results revealed redox potentials of −191 ± 6 mV for Etrx1, −282 ± 16.5 mV for Etrx2, −132.8 ± 5.9 mV for MsrA2 and −120.9 ± 0.6 mV for MsrB2 (Fig 5A). The apparent redox potential of purified MsrAB2 has also been analysed and was determined between the redox potential of the individual MsrA2 and MsrB2 domains, confirming the previous results. Thus, thermodynamically, the transfer of electrons from both Etrx proteins to the Msr subunits of *Sp*MsrAB2 would be possible. In order to elucidate whether both Etrx proteins can transfer electrons to *Sp*MsrAB2 kinetically, the NADPH-dependent methionine sulfoxide reductase activity of rMsrA2 and rMsrB2, respectively, was measured in kinetic experiments with Etrx1 or Etrx2 as electron donor. Calculation of the specific activities indicated that Etrx1 efficiently regenerates oxidized MsrA2 but not MsrB2. In contrast, Etrx2 can reduce MsrB2 but has a 3.6-fold lower activity for the reduction of MsrA2 compared to Etrx1 (Fig 5B and Table2).

1. Redox potential of Etrx proteins and methionine sulfoxide reductases. The redox potential was determined in the presence of different ratios of glutathione (GSH) and glutathione disulfide (GSSG) by specific alkylation using AMS in an anaerobic nitrogen environment. Free thiols were alkylated which resulted in a shift of 536 Da per alkylated thiol. The redox potential was calculated and the CBB staining shows at this point similar ratios of the oxidized and reduced protein forms.
2. The kinetic parameters of Etrx proteins for the methionine sulfoxide reductase subunits MsrA2 and MsrB2 were determined in the presence of NADPH as described in the Supporting Information. Activity was determined in an optical assay from the decrease in *A*_340 nm_. Each data point represents the mean value of two/three independent experiments.
3. Diagonal non-reducing/reducing SDS–PAGE combined with *Sp*MsrAB-specific immunoblot analysis showed accumulation of *Sp*MsrAB2 intramolecular disulphides (marked in red) particularly in the D39Δ*cps*Δ*ccdA1*Δ*ccdA2* and D39Δ*cps*Δ*etrx1*Δ*etrx2* double mutants *in vivo*. The diagonal non-reducing/reducing immunoblot analyses were performed using whole cell protein extracts of parental strain D39Δ*cps* (a) and isogenic mutants D39Δ*cps*Δ*ccdA1* (b), D39Δ*cps*Δ*ccdA2* (c), D39Δ*cps*Δ*ccdA1*Δ*ccdA2* (d and e), D39Δ*cps*Δ*msrAB1* (f), D39Δ*cps*Δ*msrAB2* (g) and D39Δ*cps*Δ*etrx1*Δ*etrx2* (h), respectively, as described previously (Pother et al, 2009). Pneumococcal protein extracts were harvested from bacteria cultured under non-stress conditions and proteins with reduced thiol-groups were alkylated with 20 mM NEM. Proteins were first separated using a non-reducing SDS–PAGE without DTT (1D) and the lane was cut and separated horizontally by a second reducing SDS–PAGE with DTT (2D). The reducing diagonal SDS-gel (2D) was subjected to *Sp*MsrAB-and pneumococcus-specific immunoblot analysis. The non-reducing one-dimensional gel (1D) was subjected to *Sp*MsrAB immunoblot analysis and is shown on top of the corresponding diagonal immunoblot (a–d). In the diagonal immunoblots the *Sp*MsrAB intramolecular disulphides were detected using the anti-MsrAB antiserum alone as shown for D39Δ*cps*Δ*msrAB1* (f), D39Δ*cps*Δ*msrAB2* (g) and the double mutant D39Δ*cps*Δ*ccdA1*Δ*ccdA2* (e), or with anti-MsrAB2 antiserum in combination with the anti-pneumococcus antiserum (a–d, h) to visualize the reduced proteins migrating along the diagonal. *Sp*MsrAB2 appeared in two isoforms as indicated in the diagonal assay by the blue circles. The lower isoform of *Sp*MsrAB2 overlapped with *Sp*MsrAB1 as revealed by the immunoblots of the *msrAB1-* and *msrAB2-*mutants (f and g) and as shown by the immunoblot in Fig 1D. The *Sp*MsrAB intramolecular disulphides migrate slightly above the diagonal (red circles) and accumulate especially in the *ccdA1/2* and *etrx1/2* double mutants suggesting that *Sp*MsrAB2 is more oxidized in the double knockouts compared to the single *ccdA*-mutants. Grey circles show proteins with intramolecular disulphides detected by anti-pneumococcal antibodies. The grey arrowhead in (e) indicates a non-specific signal.

**Table 2:** Kinetic parameters of methionine sulfoxide reductase activity of MsrA2 or MsrB2

Enzyme–substrate	*V*_max_	*K*_m_	Efficiency (*V*_max_/*K*_m_)
	nmol/mg min	µmol/L	AU
MsrA2 + Etrx1	59.0	26.5	2.22
MsrA2 + Etrx2	8.2	13.3	0.61
MsrB2 + Etrx1	(No activity)	(No activity)	(No activity)
MsrB2 + Etrx2	17.1	21.0	0.81

The methionine sulfoxide reductase activity was measured in the presence of Etrx1 and Etrx2 protein, respectively. The reaction was performed at pH 7.4 in a mixture containing the Etrx protein, NADPH, human thioredoxin reductase and the reaction was started by the addition of MsrAB2 subunits.

AU, arbitrary units.

The redox state of *Sp*MsrAB2 was analysed *in vivo* for the *ccdA* single mutants as well as the *ccdA1/2* and *etrx1/2* double mutants using diagonal non-reducing/reducing SDS–PAGE assays followed by immunoblot analysis with anti-*Sp*MsrAB2 and anti-pneumococcal antisera (Fig 5C). This diagonal assay distinguishes intramolecular and intermolecular disulphides in proteins (Leichert & Jakob, 2006). Bacterial extracts of non-encapsulated pneumococci were harvested under non-stress control conditions and proteins with reduced thiol-groups were irreversibly alkylated with NEM (*N*-ethylmaleimide), while disulphide bonds within the same protein and between different proteins are maintained. In this diagonal assay pneumococcal protein extracts were separated in the first dimension by non-reducing SDS–PAGE, the lane was cut and separated horizontally by a second reducing SDS-PAGE. Proteins with no disulphides run along the diagonal, while intramolecular disulphides migrate slightly above the diagonal. The diagonal immunoblot analysis revealed several *Sp*MsrAB2 isoforms in the reduced form that were detected along the diagonal with the anti-*Sp*MsrAB2 antiserum (Fig 5C). The lower *Sp*MsrAB2 isoform probably is mixed with *Sp*MsrAB1 as shown by *msrAB1*- and *msrAB2*-mutant blots (Fig 5C; see also Fig 1D). Importantly, the oxidized intramolecular disulphide of the upper *Sp*MsrAB2 isoform accumulates strongly above the diagonal in the D39Δ*cps*Δ*ccdA1*Δ*ccdA2* and D39Δ*cps*Δ*etrx1*Δ*etrx2* mutants (Fig 5C), but is only weakly detected in the single *ccdA* mutants. The results suggest that *Sp*MsrAB2 is more oxidized in the *ccdA1/2* and *etrx1/2* double mutants compared to the single mutants. The functions of Etrx1 and Etrx2 as electron partners for *Sp*MsrAB2 were further demonstrated by growth experiments in the presence of 6 mM free MetSO as physiological Msr substrate. Growth in the presence of free MetSO was significantly impaired in the *msrAB2* single or *etrx1/2* and *ccdA1/2* double mutants compared to the *etrx* or *ccdA* single mutants (Supporting Information Fig S15). This MetSO-sensitive phenotype is indicative for the deficiency of methionine sulfoxide reductase activity in the absence of both functional CcdA-Etrx electron pathways *in vivo* (Supporting Information Fig S15).

### Etrx1 and Etrx2 are required for full virulence of pneumococci in an acute pneumonia mouse infection model

**Figure 6:**
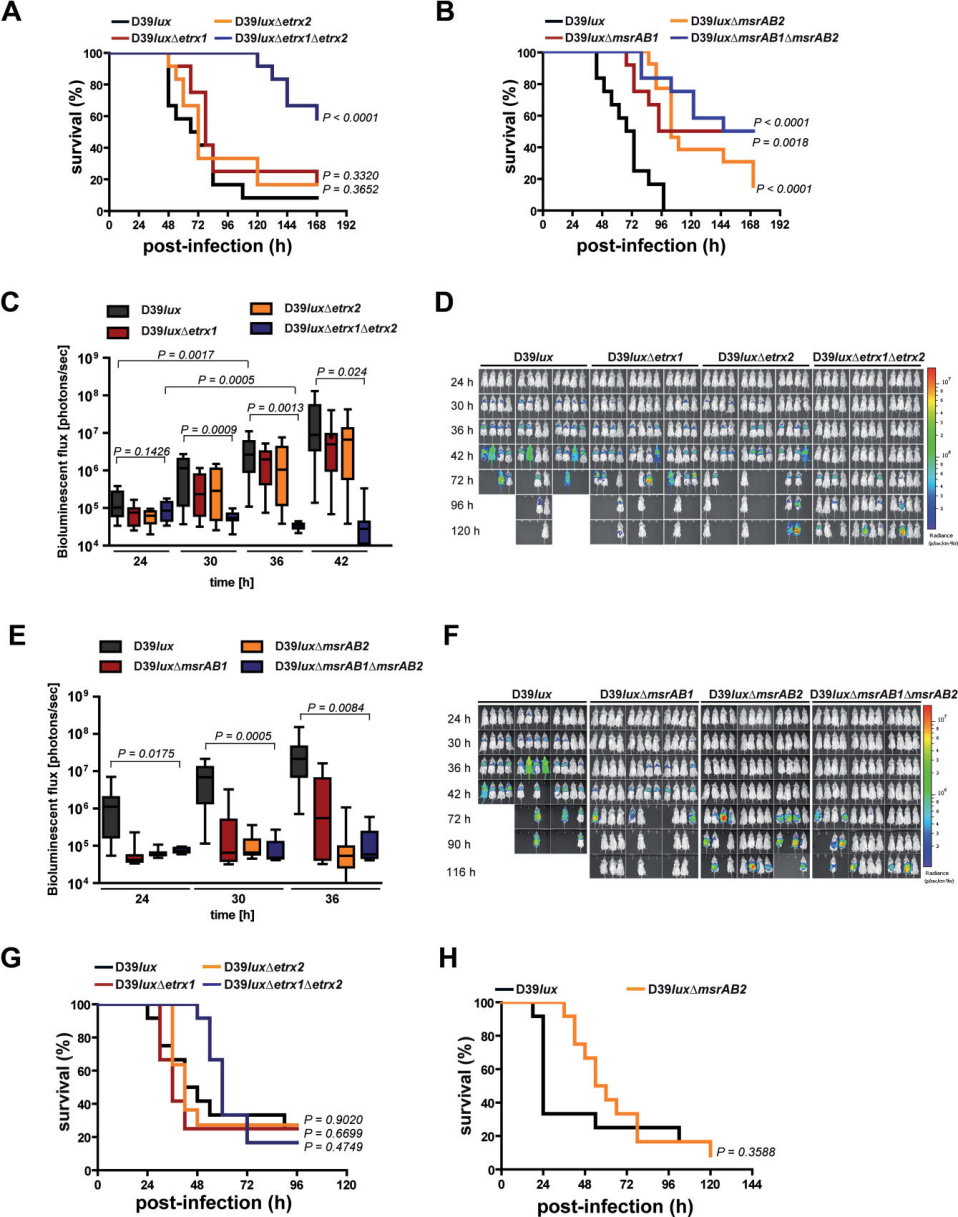
Impact of the Etrx proteins on pneumococcal virulence in mice

The acute experimental pneumonia and sepsis infection models were applied to assess the role of thioredoxin-like lipoproteins Etrx1 and Etrx2 on pneumococcal colonization and virulence in CD-1 outbred mice. In the acute pneumonia model mice (*n* = 12) were challenged intranasally with 1.0 × 10^7^ bioluminescent wild-type D39*lux* or its isogenic mutants D39*lux*Δ*etrx1*, D39*lux*Δ*etrx2* and D39*lux*Δ*etrx1*Δ*etrx2*, respectively. Mice infected with wild-type pneumococci or single *etrx* mutants showed the first weak signs of pneumococcal spread into the lungs at 30 h post-infection. In contrast, the D39*lux*Δ*etrx1*Δ*etrx2* double mutant showed earliest at 72 h post-infection a strong increase in bioluminescence in the lungs as monitored by real-time bioimaging, which could be correlated with a strong increase in bacterial load in the lungs (Fig 6). This delay of pneumococcal pneumonia and septicemia after intranasal infection suggested an attenuation of virulence for the double mutant D39*lux*Δ*etrx1*Δ*etrx2*. In contrast, the single knockout mutants D39*lux*Δ*etrx1* and D39*lux*Δ*etrx2* showed no significant differences in bioluminescent flux compared to D39*lux* and had mostly developed severe lung infections or succumbed to sepsis 72 h post-infection (Fig 6D). The results of the real-time monitoring correlated with the survival rates of mice. The intranasal infection with the double mutant D39*lux*Δ*etrx1*Δ*etrx2* prolonged significantly the survival time of mice (*p* < 0.0001), whereas survival of mice infected with the Etrx1- or Etrx2-deficient single mutants was not significantly altered compared to the wild-type infected mice (Fig 6A). Similar to the H_2_O_2_ resistance only the *etrx* double mutant is affected in virulence during *in vivo* infection, while in the study of Andisi et al the attenuation of the mutant is due to the deficiency of Etrx1 (TlpA) and *Sp*MsrAB (Andisi et al, 2012). To investigate whether the deficiency of *Sp*MsrAB2 results in a phenotype similar to the double mutant D39*lux*Δ*etrx1*Δ*etrx2*, mice were also infected intranasally with the *msrAB*-mutants. Indeed, the results revealed significant attenuation of the *msrAB2*-mutant (*p* < 0.0001) compared to the wild-type D39*lux* (Fig 6B, E and F). Importantly, the *etrx*-mutants and the *msrAB2*-mutant showed no growth defects under *in vitro* conditions (Supporting Information Fig S16). In contrast, the deficiency of *Sp*MsrAB1 impaired growth in a chemically defined medium (Supporting Information Fig S16) and pneumococcal virulence was also significantly reduced (Fig 6B) as has been shown previously (Wizemann et al, 1996). The real-time monitoring correlates with the survival rates of the mice (Fig 6E and F) and the lack of *Sp*MsrAB1 and *Sp*MsrAB2 had no additive effect. Furthermore, CD-1 mice (*n* = 9) were infected with a lower infection dose (1 × 10^6^) to explore the effect of Etrx proteins or *Sp*MsrAB2 on nasopharyngeal colonization in the carriage model. Pneumococci were recovered after 1, 3 and 5 days from the nasopharynx and the lung by a bronchioalveolar lavage. These results showed at all time points a significant reduction of nasopharyngeal carriage for the double mutant D39*lux*Δ*etrx1*Δ*etrx2* compared to the wild-type (Supporting Information Fig S17). The other *etrx*- or *msrAB2*-mutants did not significantly differ in nasopharyngeal carriage from the isogenic wild-type (Supporting Information Fig S17). However, the *msrAB2*-mutant showed immediately at 24 h post-infection a significantly reduced number of CFU in the lung (Supporting Information Fig S17B).

A,B. Survival of CD-1 mice after intranasal infection with pneumococci. Groups of mice (*n* = 12) were intranasally infected with 10^7^ CFU of bioluminescent *S. pneumoniae* D39 wild-type (D39*lux*) or its isogenic mutants deficient for Etrx (A) or MsrAB proteins (B).
C–F Bioluminescent optical imaging of pneumococcal dissemination after intranasal infection of CD-1 mice (*n* = 12). Dissemination of bioluminescent D39*lux*, and isogenic mutants deficient for Etrx1, Etrx2, Etrx1 and 2 (D), MsrAB1, MsrAB2 or MsrAB1 and 2 (F) were analysed at indicated time points by determination of the luminescence intensity (photons/s) measured with the IVIS® Spectrum system. The bioluminescent flux of grouped mice is shown for indicated time points in the box whisker graph (C and E).
G,H. Survival of CD-1 mice in a systemic infection model. A CFU of 5 × 10^3^ pneumococci was used for intraperitoneal infection of mice (*n* = 12).

To analyse the impact of the Etrx and *Sp*MsrAB2 proteins on pneumococcal survival during sepsis, mice were infected via the intraperitoneal route. The survival rates of mice (*n* = 12) were similar for groups infected with wild-type or single *etrx* mutants, although there was a moderate but not significant attenuation for the double mutant D39*lux*Δ*etrx1*Δ*etrx2* (Fig 6G). Similarly, the *msrAB2*-mutant showed no significant attenuation during invasive disease (Fig 6H).

Taken together, these *in vivo* infection experiments suggest that loss of one of the Etrx lipoproteins does not affect virulence. In contrast, loss of function of either surface-exposed Etrx lipoproteins or the *Sp*MsrAB2 protein does significantly reduce virulence and spread of pneumococci from the nasopharynx into the lungs and blood.

### Pneumococcal resistance against killing phagocytosis relies on Etrx and *Sp*MsrAB2

**Figure 7:**
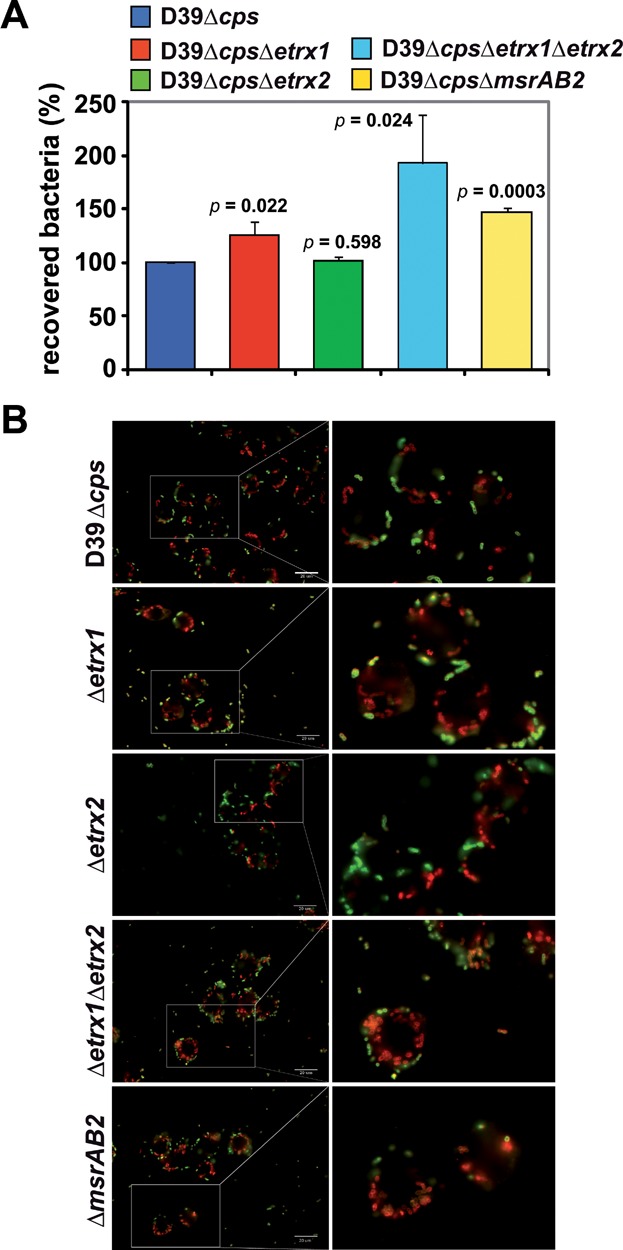
Influence of extracellular thioredoxin lipoproteins and MsrAB2 deficiency on uptake of *S. pneumoniae* D39Δ*cps* by macrophages

To investigate the role of the Etrx and *Sp*MsrAB2 proteins on uptake by professional phagocytes and to allow significant phagocytosis non-encapsulated pneumococci were incubated with macrophages. The results showed significantly higher numbers of internalized and recovered D39Δ*cps*Δ*etrx1*Δ*etrx2* pneumococci deficient in both Etrx proteins compared to the isogenic D39Δ*cps* and individual *etrx*-mutants (Fig 7A). The number of recovered pneumococci was also significantly increased for the *msrAB2*-mutant (Fig 7A). The increased number of phagocytosed *etrx* double mutants or *msrAB2*-mutants was also confirmed by immunofluorescence microscopy (Fig 7B). Remarkably, the lack of Etrx1 in D39Δ*cps* also accelerated phagocytosis (Fig 7A). In addition, the intracellular fate of wild-type and *etrx*-mutants was assessed. Regarding the intracellular survival, all strains showed a time-dependent decrease in the number of recovered and viable pneumococci. However, the relative decline of recovered D39Δ*cps*Δ*etrx1*Δ*etrx2* mutants did not show significant differences compared to the non-encapsulated *S. pneumoniae* D39Δ*cps*. Similar to the non-encapsulated pneumococci, phagocytosis of the encapsulated strains showed a higher number of phagocytosed and recovered mutants deficient in both Etrx proteins. These data suggest that the total loss of Etrx lipoprotein or *Sp*MsrAB2 activity accelerates phagocytosis and hence, killing of pneumococci.

1. J774 cells were infected with a multiplicity of infection of 50 non-encapsulated D39Δ*cps* and isogenic *etrx*-mutants per cell. The internalization of the bacteria was determined by applying antibiotic protection and quantitative plating 30 min post-infection. Experiments were done at least three times in triplicate and data represent the mean ± SEM.
2. Immunofluorescence microscopy of pneumococci attached (green) to J774 macrophages and phagocytosed, intracellular pneumococci (red) 30 min post-infection. Attached bacteria are stained green (Alexa Fluor® 488), while intracellular bacteria are stained red (Alexa Fluor® 568).

## DISCUSSION

### Thioredoxin lipoproteins are required for resistance to extracellular oxidative stress

In this study, we performed a comprehensive functional and structural analysis of the extracellular oxidative stress resistance system of *S. pneumoniae* mediated by two CcdA-Etrx pathways and their redox partner *Sp*MsrAB2. Our results demonstrated that single *etrx*-mutants expressing *Sp*MsrAB2 showed lower susceptibility to killing by H_2_O_2_, compared to the *etrx* double mutant that had a similar peroxide sensitive phenotype like the *Sp*MsrAB2 mutant. Thus, only the deficiency of both Etrx1 and Etrx2 proteins or *Sp*MsrAB2 significantly accelerated pneumococcal killing by H_2_O_2_ and diminished growth in the presence of MetSO, respectively. Similarly, only the deficiency of both, Etrx1 and Etrx2, or their electron acceptor *Sp*MsrAB2 attenuates significantly pneumococcal virulence in the acute pneumonia model but not in the sepsis model. The importance of the Etrx proteins and *Sp*MsrAB2 for virulence functions is supported by our phagocytosis assays, which showed that only the uptake of the *etrx* double or *msrAB2* mutant is massively enhanced, whereas only a minor effect was observed for the single *etrx*-mutants. These data suggest that the Etrx proteins can functionally replace each other, which ensures proper function of surface-exposed pneumococcal proteins. The determination of the redox potentials for the Etrx and *Sp*MsrAB2 proteins together with our structural and kinetic data further demonstrate that *Sp*MsrAB2 can be reduced by both Etrx proteins. Interestingly, Etrx1 seems to reduce preferentially the MsrA2 subunit of *Sp*MsrAB2 while Etrx2 is able to reduce both MsrA2 and MsrB2 domains *in vitro* (Fig 5). Importantly, diagonal non-reducing/reducing SDS–PAGE analysis combined with *Sp*MsrAB2 specific immunoblots further verified the *in vivo* accumulation of oxidized *Sp*MsrAB2 protein especially in the *ccdA1/2* and *etrx1/2* double mutants. This indicates that both thioredoxin systems are required for efficient regeneration of *Sp*MsrAB2. Interestingly, we detected strong growth sensitivities of the *etrx1/2* double mutant in the presence of H_2_O_2_ and free MetSO as physiological MsrAB2 substrate. Hence, we postulate that both extracellular CcdA-Etrx-*Sp*MsrAB2 electron pathways are involved in reduction of oxidized Met residues present in surface-exposed virulence proteins or as free MetSO on the bacterial surface that has to be further explored in future studies.

### Structural determinants of Etrx1 and Etrx2 proteins and evidence for two CcdA-Etrx-*Sp*MsrAB2 pathways

Thiol-disulfide oxidoreductases (TDORs) comprise a large superfamily of proteins that are present in all kingdoms of life where they control the redox state of Cys containing proteins. Cytoplasmic TDORs like the thioredoxin system are usually involved in maintaining protein cysteines in a reduced state (Fernandes & Holmgren, 2004). In contrast, periplasmic TDORs of Gram-negative bacteria like the Dsb family proteins or extracellular TDORs of Gram-positive bacteria catalyse disulphide bond formation or isomerization in the oxidizing periplasm or extracellular space (Cho & Collet, 2013; Denoncin & Collet, 2013). These extra-cytoplasmic or periplasmic TDORs are involved in a wide range of processes, including cytochrome maturation, *e.g. B. subtilis* ResA (Lewin et al, 2006), cell motility, *e.g. E. coli* DsbB (Dailey & Berg, 1993), natural competence development, *e.g. B. subtilis* BdbD (Meima et al, 2002), toxin biosynthesis, *e.g. E. coli* DsbA (Yamanaka et al, 1994) and synthesis of the endospore peptidoglycan cortex protective layer, *e.g. B. subtilis* StoA (Crow et al, 2009). Periplasmic reducing systems are present in this oxidizing compartment of Gram-negative bacteria to reduce sulfenic acids in the periplasm (*e.g*. DsbDG) or to deliver electrons for MetSO reduction (*e.g*. PilB) (Cho & Collet, 2013). Both Etrx1 and Etrx2 share sequence homology (30.48 and 21.83%, respectively) with the C-terminal periplasmic domain of DsbD. CcdA1 and CcdA2 are homolog to the membrane-embedded domain of DsbD (Supporting Information Fig S10). Hence, we postulate that the CcdA-Etrx-MsrAB2 pathways also function in the extracellular compartment of the Gram-positive pneumococci in MetSO reduction.

According to the accepted catalytic mechanism of thioredoxin-like proteins (Crow et al, 2004), Etrx1/Etrx2 would bind their redox partner *Sp*MsrAB2 by means of a hydrophobic surface and subsequently perform a nucleophilic attack on the target disulphide bond via the N-terminal nucleophilic thiolate of the CXXC motif (Cys84 in Etrx1, Cys81 in Etrx2). This process would lead to the formation of a mixed intermolecular disulphide between Etrx and its redox partner proteins, which is resolved by the second C-terminal resolving Cys residue (Cys87 in Etrx1, Cys84 in Etrx2). The N-terminal active-site Cys residue is present in the reactive thiolate anion form, which is stabilized by an interaction with the dipole of helix α2. In several thiol-disulphide oxidoreductases this interaction reduces the p*K*_*a*_ of the nucleophilic active-site Cys by at least two pH units (Roos et al, 2013).

In the CXXC motif of both Etrx proteins, only the active-site Cys is solvent-exposed and accessible to the redox partner (Cys84 in Etrx1 and Cys81 in Etrx2). Many thioredoxin-like proteins share also a proline residue within the CXXC motif. This proline residue is also present in the CXXC motif of Etrx2 (Fig 3D), but not in Etrx1. The presence of proline residues at the cap of the active site helix has been reported to have important consequences for the distribution of the electrostatic field near the cysteines as proline does not possess a standard peptide group (Crow et al, 2009). The absence of proline in Etrx1 would affect the macrodipole arising from α2 helix that is often invoked as primary cause of the lowered p*K*_*a*_ values associated with the active-site cysteine residue of the CXXC motif in most TDORs (Kortemme & Creighton, 1995). Furthermore, the limited conformational freedom of proline (in comparison with other residues) has been reported to be an important factor in maintaining rigidity of the CXXC motif and relevant for the structural changes from the reduced to its oxidized forms. Etrx1, representing one of the rare cases without proline in the CXXC motif, is therefore expected to have more structural variations between reduced and oxidized forms than Etrx2. Another relevant difference between both Etrx proteins and other extra-cytoplasmic TDORs concern a glutamate residue that is placed three positions after the C-terminal cysteine residue of the CXXC motif. Substitution of this glutamate has been shown to have a significant effect on the active site properties of ResA and StoA of *B. subtilis* (Crow et al, 2009; Hodson et al, 2008; Lewin et al, 2006). Etrx1 and Etrx2 do not have a glutamate at this position, but possess instead a serine residue (Ser90 in Etrx1 and Ser87 in Etrx2; Supporting Information Fig S18).

Etrx2 has an unprecedented hydrophobic cavity close to the active site. The presence of a hydrophobic patch near the active site has been associated with substrate recognition in other TDORs (Crow et al, 2009). In the structure of the oxidized forms of Etrx2, a detergent molecule (Cyclofos-3™) or a HED molecule is bound to this hydrophobic pocket, very likely mimicking the redox partner interaction. Etrx1 lacks this cavity and this region shows differences in both the nature of the amino acids and in the conformation of the β4–α3 loop (Fig 3). In agreement with these results, soaking experiments with Etrx1 crystals did not yield a complex with Cyclofos-3™ even at high concentrations of the detergent.

In conclusion, some of the structural determinants of the CXXC motif observed in other extra-cytoplasmic thioredoxins, such as the presence of proline or glutamate residues are not observed in pneumococcal Etrx proteins (except for the proline in Etrx1). Despite strong similarities in the overall fold of both, Etrx1 and Etrx2, relevant differences are observed between their active sites (presence of hydrophobic cavity in Etrx2, different electrostatic potential, different CXXC motifs). These differences provide a structural basis for the specific interaction of Etrx with the MsrA or the MsrB domains of the *Sp*MsrAB2 redox partner observed in the redox potential determination and kinetics.

### Model for the two CcdA-Etrx-*Sp*MsrAB2 electron pathways

**Figure 8:**
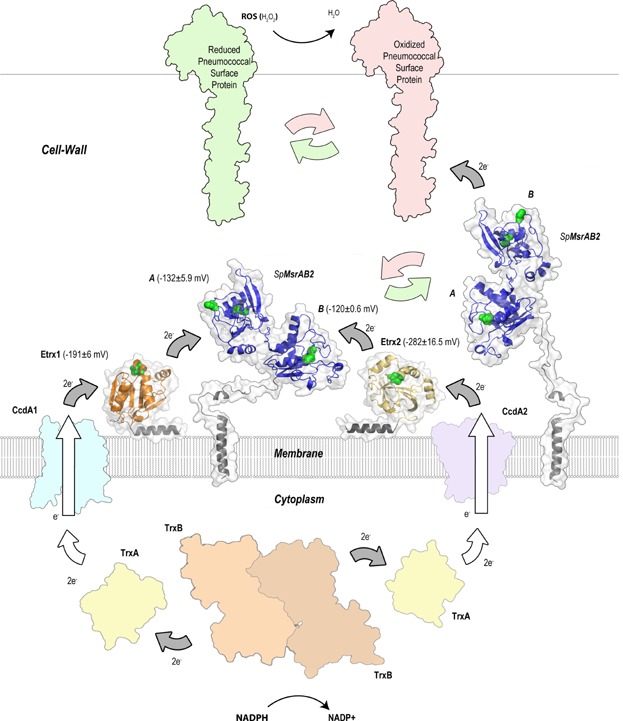
Proposed mechanism of oxidative stress defense mediated by the CcdA1-Etrx1 and CcdA2-Etrx2 electron pathways and their redox partner *Sp*MsrAB2 on the pneumococcal surface Electrons provided from NADPH by the cytoplasmic TrxB (SPD_1287) are transferred to the cytoplasmic TrxA (SPD_1567) and shuttled between the integral membrane protein CcdA1 (SPD_0571) to the surface-exposed thioredoxin-like Etrx1 (SPD_0572), and between the integral membrane protein CcdA2 (SPD_0885) to surface-exposed thioredoxin-like Etrx2 (SPD_0886) following the same mechanism. Both thioredoxin-like proteins provide reducing equivalents to *Sp*MsrAB2 (SPD_0573) for the reduction of the MsrA (by Etrx1 or Etrx2) and MsrB (by Etrx2) domains. *Sp*MsrAB2 remains anchored to the membrane and presents a long and flexible coiled coil region allowing *Sp*MsrAB2 to reach and repair ROS damaged surface proteins. Catalytic cysteine residues in Etrx1, Etrx2 and *Sp*MsrAB2 are represented as green spheres. Experimentally determined redox potentials for each protein are labelled. White arrows indicate presumed interactions and grey arrows indicate those demonstrated experimentally. All accession numbers refer to the *S. pneumoniae* D39 annotation.

The mechanism for the protection against oxidative stress via both CcdA-Etrx-*Sp*MsrAB2 electron pathways is modeled in Fig 8 and Supplementary Information Movie 1. The pneumococcal cell wall is an oxidizing environment in which the sulphur-containing amino acids Met and cysteine are highly susceptible to oxidation by endogenously produced peroxide. Electrons are transported from the cytoplasmic NADPH pool to the cell wall to keep pneumococcal surface proteins in a reduced state. The first proteins of this extracellular electron transport system are the integral membrane proteins CcdA1 and CcdA2. Electrons from the cytoplasmic Trx are shuttled between CcdA1 and CcdA2 to Etrx1 and Etrx2, respectively, similarly to that observed between the transmembrane and the periplasmic domains of DsbD. Surface-exposed Etrx1 and Etrx2 deliver electrons to the *Sp*MsrAB2 protein for the reduction of MsrA2 (by Etrx1 or Etrx2) and MsrB2 (by Etrx2) domains (Fig 8). Oxidation of Met results in a mixture of the two diastereomers methionine-*S*-sulfoxide and methionine-*R*-sulfoxide, which are reduced by MsrA and MsrB, respectively. Besides the catalytic domains, *Sp*MsrAB2 also carries a transmembrane segment that anchors the protein to the membrane. It has also a long and flexible coiled coil region allowing the enzyme to reach damaged virulence proteins and to reduce MetSO (Fig 8 and Supporting Information movie). Thioredoxin-like lipoproteins Etrx1/Etrx2 are critical in the turnover of the system by reducing the methionine sulfoxide reductase *Sp*MsrAB2. Since this seems to be the sole extracellular thioredoxin system of pneumococci, the lack of functional Etrx proteins or *Sp*MsrAB2 protein has direct consequences to resist oxidative stress and host immune defense mechanisms. In this sense, the pneumococcal surface-exposed thioredoxin systems reported here provide an important framework for the development of new antibacterial therapies.

## MATERIALS AND METHODS

### Bacterial strains, culture conditions and transformation techniques

*E. coli* strains and *S. pneumoniae* genotypes and strains used in this study are listed in Supporting Information Table S1. *E. coli* and *S. pneumoniae* strains were cultured and transformed as described recently (Jensch et al, 2010).

### Primers, construction of pneumococcal mutants and protein purification

Primers that were used in this study and plasmids used for the mutagenesis and recombinant protein expression are listed in Supporting Information Table S2. For the generation of the pneumococcal mutants in D39*lux* (Jensch et al, 2010) and D39Δ*cps* the insertion–deletion mutagenesis strategy was used as described (Rennemeier et al, 2007). Mutagenesis, expression cloning and protein purification of His_6_-tagged proteins are described in detail in the Supporting Information.

### Pneumococcal survival under oxidative conditions

The survival experiments under oxidative condition with hydrogen peroxide (H_2_O_2_), paraquat (stimulating superoxide production in cells; kindly provided by A. Littmann, Julius Kühn Institute (JKI), Braunschweig, Germany) or MetSO (Sigma–Aldrich, Taufkirchen, Germany) were conducted as described previously (Johnston et al, 2004). Briefly, wild-type pneumococci and Etrx-deficient mutants were cultured in THY broth at 37°C to mid-log phase and treated for 30 min with 10, 15 or 20 mM H_2_O_2_ and 90 min with 0.25, 0.5 or 0.75 mM paraquat, respectively. Untreated pneumococci were used as a control. To determine the percentage of survival, serial dilutions were plated onto blood agar plates and CFU were counted after overnight incubation at 37°C and 5% CO_2_. In control experiments catalase (5000 U/ml) was added simultaneously with H_2_O_2_ to the bacterial cultures.

### Protein crystallization

Etrx1 crystals were obtained with 30% v/v PEG 4000; 0.1 M tris pH 8.5; 0.2 M MgCl_2_ 18°C. Etrx2:Cyclofos 3™ complex crystal was obtained with 3.4 M sodium malonate pH 6.0 18°C, while the Etrx2:HED Complex crystal was obtained in 30% PEG 1500 supplemented with 14 mM β-mercaptoethanol. Details in Supporting Information.

### Data collection, phasing and model refinement

Native data sets of Etrx1 and Etrx2: Cyclofos-3™ crystals were collected on ESRF ID14-4 beamline in Grenoble, France. Native data set of Etrx2:HED crystal was collected on SLS PXIII beamline in Villigen, Switzerland (Table1). Details in Supporting Information.

### *Sp*MsrAB2 homology model

The 3D model for the catalytic domains of pneumococcal *Sp*MsrAB2 (residues 60–312) was obtained by comparative homology via modeler and energy minimization.

### Ethics statement

Animal experiments were performed in strict accordance with the German regulations of the Society for Laboratory Animal Science (GV-SOLAS) and the European Health Law of the Federation of Laboratory Animal Science Associations (FELASA). All experiments were approved by the ethical board and Landesamt für Landwirtschaft, Lebensmittelsicherheit und Fischerei Mecklenburg-Vorpommern (LALLFV MV), Rostock in Germany (permit no. 7221.3-1.1-006/09 and 7221.3-1.1-019/11).

### Mouse models of infection and bioluminescent optical imaging

Eight weeks old female outbred CD1 mice (Charles River, Sulzfeld, Germany) were infected intranasally or intraperitoneally with bioluminescent pneumococci as described recently (Hartel et al, 2011; Jensch et al, 2010). Briefly, pneumococci were cultured to *A*_600_ = 0.35 in THY supplemented with 10% foetal bovine serum and the infection dose was adjusted to 1.0 × 10^7^ CFU in 25 µl for the intranasal route (*n* = 12) and 5 × 10^3^ CFU in 100 µl for the intraperitoneal route (*n* = 12). Before intranasal infection, mice were anaesthetized by intraperitoneal injection of ketamine (Ketanest S; Pfizer Pharma, Karslruhe, Germany) and xylazine (Rompun®; Provet AG, Lyssach, Germany). Once anesthetized the animals were scuffed, with the nose held upright, and the bacterial suspension of 25 µl was administered intranasally by adding a series of small droplets into the nostrils for the mice to involuntarily inhale. The infection dose was confirmed by determination of the CFU after plating serial dilutions of the infection dose on blood agar plates. Bioluminescent optical imaging using the IVIS® Spectrum Imaging System (Caliper Life Sciences, Hopkinton, US) allowed monitoring of pneumococcal dissemination after intranasal infection (Hartel et al, 2011; Jensch et al, 2010). At pre-chosen time intervals post-infection mice were imaged for 1 min to monitor dissemination of pneumococci into the lungs. A time series of the images was generated and the bioluminescent intensity (BLI) was determined by quantification of the total photon emission using the LivingImage® 4.1 software package (Caliper Life Sciences).

### Phagocytosis experiments

To determine the rate of phagocytosed wild-type and mutant pneumococci and their intracellular survival in macrophages, phagocytosis experiments with J774A.1 murine macrophages (DSMZ, Braunschweig, Germany) were carried out as described (Hartel et al, 2011; Jensch et al, 2010).

### Statistical analysis

All data are reported as mean ± SD unless otherwise noted. Results were statistically analysed using the unpaired two-tailed Student's test. Kaplan–Meier survival curves were compared by the log rank test. *p* Values for bioluminescence measurements were calculated using the unpaired, one-tailed *t*-test for differences between groups, while differences of one group between days were analysed by the paired *t*-test. Statistical significance was confirmed by ANOVA analysis with Bonferroni's multiple comparison *post hoc* test. A *p*-value <0.05 was considered to be statistically significant.

For more detailed materials and methods see the Supporting Information.

### Accession numbers

Sequence data for the *etrx* genes 1 and 2 of D39 or TIGR4 are deposited in the EMBL/GenBank databases under accessions numbers ABJ55360 and ABJ55355 or AAK74804 and AAK75117. Sequence data for the *ccdA1* and *ccdA2* of D39 or TIGR4 are deposited in the EMBL/GenBank databases under accession numbers ABJ54003 and ABJ54567 or AAK74803 and AAK75116. Sequence data for the *msrAB2* gene of D39 or TIGR4 are available from the EMBL/GenBank databases under accessions numbers ABJ53896 or AAK74805. The atomic coordinates and structure factors for Etrx1, Etrx2:Cyclofos-3™ complex and Etrx2:HED complex (codes 4HQS, 2YP6 and 4HQZ, respectively) have been deposited in the Protein Data Bank, Research Collaboratory for Structural Bioinformatics, Rutgers University, New Brunswick, US (http://www.rcsb.org/).

### The paper explained

**PROBLEM**

The respiratory pathogen *Streptococcus pneumoniae* (the pneumococcus) is a serious pathogen causing life-threatening community-acquired pneumonia and invasive diseases. The high morbidity and mortality caused by pneumococcal diseases (more than 1.5 million every year, particularly in infants, elderly and immunocompromised patients), is exacerbated by the increasing prevalence of antibiotic-resistant strains and the suboptimal efficacy of available vaccines. Pneumococci have evolved efficient mechanisms to resist protein damage under oxidative stress conditions and to displace other bacteria in the nasopharynx. While oxidative stress-resistance mechanisms in the cytoplasm are well studied, the extracellular mechanism required to resist attack from the host is less investigated.

**RESULTS:**

We have identified a two-operon system responsible for the extracellular oxidative stress resistance. This system is composed of two integral membrane proteins (CcdA1 and CcdA2), two thioredoxin-like lipoproteins (Etrx1 and Etrx2) and a single methionine sulfoxide reductase (*Sp*MsrAB2). We have solved the crystal structures of both Etrx proteins and analysed the functions of both Etrx and *Sp*MsrAB2 proteins on oxidative stress resistance and virulence. We further observed in phagocytosis experiments with macrophages that both thioredoxin lipoproteins and *Sp*MsrAB2 play a crucial role in pneumococcal pathogenesis. We can finally conclude that both Etrx proteins function as electron donors for the *Sp*MsrAB2 redox partner and are therefore crucial for the extracellular reducing redox pathways of pneumococci.

**IMPACT:**

The data highlight the crucial role of thioredoxin lipoproteins Etrx1 and Etrx2 and *Sp*MsrAB2 for virulence and the redox-reactions of the extracellular oxidative stress resistance mechanism of pneumococci. Suppression of that system severely reduces pneumococcal virulence and lethality. In this sense, the combined effect of antibiotics with new ligands blocking this crucial pneumococcal system could be intended. Therefore, our data provide a new framework for the development of novel bactericidals against this important human pathogen.

## Author contributions

MS, JAH, CHL, HA and SH conceived and designed the experiments. MS, SGB, MRA, IJ, TMA, LP, TP and MG performed the experiments. MS, SGB, MRA, TP, JAH, CHL, HA and SH analysed the data. MS, SGB, HA, JAH and SH wrote and reviewed the paper.

## Notes

As a service to our authors and readers, this journal provides supporting information supplied by the authors. Such materials are peer reviewed and may be re-organized for online delivery, but are not copy-edited or typeset. Technical support issues arising from supporting information (other than missing files) should be addressed to the authors.
