# Supplementary material for: Molecular architecture of Streptococcus pneumoniae surface thioredoxin-fold lipoproteins crucial for extracellular oxidative stress resistance and maintenance of virulence
Source: EMBO Mol Med. 2013 Oct 18;5(12):1852–70. doi: 10.1002/emmm.201202435 (PMC3914529; doi:10.1002/emmm.201202435)
Supplement: Supplementary file 1 [file emmm0005-1852-sd1.pdf]

EMBO Molecular Medicine

Molecular architecture of *Streptococcus pneumoniae* surface thioredoxin-fold lipoproteins crucial for extracellular oxidative stress resistance and maintenance of virulence

Malek Saleh, Sergio G. Bartual, Mohammed R. Abdullah, Inga Jensch, Tauseef M. Asmat, Lothar Petruschka, Thomas Pribyl, Manuela Gellert, Christopher H. Lillig, Haike Antelmann, Juan A. Hermoso, and Sven Hammerschmidt

Corresponding authors: Sven Hammerschmidt, Interfaculty Institute for Genetics and Functional Genomics and Juan A. Hermoso, CSIC

Review timeline:

Submission date:	29 December 2012
Editorial Decision:	30 January 2013
Revision received:	14 June 2013
Editorial Decision:	10 July 2013
Revision received:	15 August 2013
Accepted:	10 September 2013

Transaction Report:

(Note: With the exception of the correction of typographical or spelling errors that could be a source of ambiguity, letters and reports are not edited. The original formatting of letters and referee reports may not be reflected in this compilation.)

Editor: Céline Carret

1st Editorial Decision 30 January 2013

Thank you for the submission of your manuscript to EMBO Molecular Medicine. We have now heard back from the two referees whom we asked to evaluate your manuscript. Although the referees find the study potentially interesting, they both have one major issue:

The function(s) of Etrx1 and Etrx2 must be tested experimentally.

Therefore, I would like to give you the opportunity to revise your manuscript, with the understanding that the referees' concerns must be fully addressed and that acceptance of the manuscript would entail a second round of review.

Please note that it is EMBO Molecular Medicine policy to allow a single round of revision only and that, therefore, acceptance or rejection of the manuscript will depend on the completeness of your responses included in the next, final version of the manuscript.

As you know, EMBO Molecular Medicine has a "scooping protection" policy, whereby similar findings that are published by others during review or revision are not a criterion for rejection. However, I do ask you to get in touch with us after three months if you have not completed your

revision, to update us on the status. Please also contact us as soon as possible if similar work is published elsewhere.

I look forward to receiving your revised manuscript.

***** Reviewer's comments *****

Referee #1 (Comments on Novelty/Model System):

All the experiments are well designed. The biggest drawback is the lack of any experiments whereby the authors test the functional impact of their structural predictions. No ethical issues arise from their model system.

Referee #1 (Remarks):

This paper provides unique mechanistic insight into how the major human pathogen Streptococcus pneumonia controls oxidative damage to extracellular proteins, an important aspect of both the basic biology and the pathogenic cascade of this pathogen. In addition to elucidating a role for the Ertx proteins in host pathogenesis, the authors also provide compelling structural data to support their mechanistic arguments. Overall, the experiments are well designed and the conclusions are for the most part well supported by the data. One of the major shortcomings of the manuscript is the lack of elucidation of the functional consequences of their structural predictions. The authors make a number of very interesting conjectures into the structural aspects of the Ertx proteins that they predict would have major effects on the function of these proteins, however none of these predictions are tested experimentally. Such lines of investigation would greatly enhance and provide more compelling evidence to support the predicted mechanisms underlying their structural modeling.

Major Points

1. The biggest concern of this manuscript is the authors have substantial and intriguing structural data which they hypothesize plays major roles in the function of the Ertx proteins however there is no experimental confirmation of any of their predictions. The manuscript would be significantly enhanced by showing that a subset of the functional predictions discussed extensively in both the Results and Discussion could be shown experimentally. This could be done via expression of the mutagenized Ertx proteins and assaying function through any of the functional assays they discuss throughout the paper.

2. Figure 1- based on the enolase it appears by Western that deletion of either Ertx protein results in the overexpression of the other- is this indeed the case? In panel C a number of the promoter and terminator sequences annotated in panel A are missing- the authors should include these in panel C for consistency.

3. Figure 2. It was slightly perplexing why the authors chose to show the FACS rather than to fractionate the cells and perform the Western Blot. This would give the readers an appreciation of the levels of the Ertx proteins in the cytoplasm, cell wall, and membrane factions respectively. The FACS does indicate that the Ertx proteins do reach the bacterial surface, though the case is stronger for Etrx2 than Etrx1. What proportion of the Ertx proteins that is surface/membrane associated is an important part of evidence to support the contention these proteins function to protect against extracellular oxidative damage.

4. In Figure 2B- have the authors investigated whether hydrogen peroxide production is altered in the Ertx mutants? Is this effect only seen with hydrogen peroxide or is this observed with other oxygen radicals?

5. Overall the murine pathogenesis experiments are well done and convincing. One potential explanation for this phenotype would be that the double mutant is defective at the initial stages of

colonization of the nasopharynx, either due to more effective initial clearance by the host or defective bacterial adherence/replication in this tissue. It might be expected that colonization of the airways would be pronouncedly different in the double mutant due to increased oxygen in this host niche. This would be an interesting clarification into the precise roles of these proteins during pathogenesis.

6. In Figure 7 the model for the most part is well done and provides the reader with how the various proteins are interacting. The one thing the authors might consider here is coloring the arrows either white or gray based on whether the interactions are based on whether the interactions have been demonstrated experimentally or are presumed to occur.

Minor Points

1. The manuscript contains numerous typographical errors- page 3 line 4 e.g. should be approximately, OH should be OH-, page 4 line 18 compartiments should be compartments, Table1 should have a space, page 20 035 should read 0.35, in the results on page 11 phagocytozed should read phagocytosed,

2. The authors indicate that the mutations did not effect production of SpMsrAB2 by Western, this should be included in the Supplementary material if possible.

3. One page 9 the authors state that the Proline reside found in the Ertx proteins 5 residues past the CxxC motif is not found in other thioredoxin like proteins, but the Figure shows that the same proline is found in ResA?

4. On page 13 in the Discussion the authors claim the lost of Ertx1/2 attenuates pneumococcus in the pneumonia and sepsis models but in the results only the pneumonia model shows a statistically significant difference.

5. In the methods the methods the bacteria were cultured both in THY and CDM media for the oxidative stress experiments but only the THY data is shown.

6. For Table 1 it would be helpful to have the PDB# included for the structures.

7. For the quantification of the luminescence, is this per mouse or was this for a selected area of the mouse- was unclear if there should be a unit of area included in this measurement.

Referee #2 (Comments on Novelty/Model System):

I think that the technical quality is low mostly because important data are missing: key experiments should be done.

The novelty is high as it is one of the first reports showing the importance of an extracellular reductive pathway in bacteria (if they provide convincing data confirming the function of Etrx1 and Etrx2).

The medical impact is high as Etrx2 and Etrx1 are attractive drug targets.

Referee #2 (Remarks):

The article by Bartual et al. reports the characterization of two surface -exposed thioredoxin proteins, Etrx1 and Etrx2, from *Streptococcus pneumoniae*. These proteins are encoded by two operons that also include a gene coding for a homolog of ccdA. CcdA is a membrane protein that transports reducing equivalents across the membrane and transfers them to extracellular proteins involved in reductive pathways. The etrx1 operon also includes a putative extracellular methionine

sulfoxide reductase (SpMsrAB2). Loss of function of Etrx1 and Etrx2 together results in a dramatic decrease in virulence and an enhanced uptake by macrophages. The authors propose that the lack of Etrx1 and Etrx2 results in the inactivation of SpMsrAB2, which would partially explain the observed phenotypes. The structures of both proteins are also presented in the paper.

Overall the work is very interesting, but the data presented are too preliminary. There is too much speculation on the function of Etrx1 and Etrx2 in the defense against oxidative stress and in the reduction of SpMsrAB2.

Specific comments:

1-what is the evidence that SpMsrAB2 is surface exposed ?

2-the authors present data (Fig 2A) suggesting that Etrx1 and Etrx2 are surface exposed. Because this is a really central result, I think that it must be confirmed using another method, such as a protease digestion assay. Moreover, more explanation should be provided regarding the results from the flow cytometric analysis.

3-page 7: The authors claim that the deletion of etrx1 has no impact on the production of SpMsrAB2 but they do not show the data. The data must be shown in the supplementary material.

4-Why did the authors use a bioluminescent strain for plating and CFU counting ?

5-What is the impact of cps deletion on the strain. This should be briefly discussed.

6-The functional characterization of Etrx1 and Etrx2 is particularly preliminary. This is clearly my major comment :

a-What is the evidence that Etrx1 and Etrx2 function in a reductive pathway ? The in vivo redox state of these proteins should be determined using classical AMS-trapping assays.

b-what is the evidence (other than the presence in the same operon) that Etrx1 is reduced by CcdA1 and Etrx2 by CcdA2. Can CcdA1 reduce ETrx2 as well ?

c-A key point : the authors claim that Etrx1 and Etrx2 can both reduce SpMsrAB2. They must show it in vitro and, possibly, in vivo (by monitoring SpMsrAB2 redox state in the single and double mutants). I don't agree when they write that "SpMsrAB2 is the only redox partner for both Etrx proteins" (page 13).

7-What is the phenotype of a ΔMsrAB2 mutant ? is it similar to the phenotype of a etrx1etrx2 mutant ?

8-I disagree with the authors when they conclude that the survival of the etrx1 or etrx2 mutant is not significantly affected by H2O2 (Fig. 2B).

9-page 13: the authors write that "the deficiency of both Etrx proteins significantly enhanced oxidative damage to proteins": maybe, but they need to provide evidence to support this conclusion.

In conclusion, this manuscript has the potential to become a very nice paper, but more work must be done to clearly and unambiguously characterize the function of Etrx1, Etrx2 and SpMsrAB2.

1st Revision - authors' response 14 June 2013

Authors response to referee´s comments on EMM-2012-02435

Manuscript title:

Molecular architecture of *Streptococcus pneumoniae* surface thioredoxin-fold lipoproteins crucial for extracellular oxidative stress resistance and maintenance of virulence

Changes made in the new (revised) version

Answers point by point

General remark:

The new results are all described in the main manuscript and due to space limitations we have shifted parts of the Materials and Methods to the Supporting Information. New parts are marked in yellow, while the track changes function of word was used to show re-written paragraphs.

Referee #1

Remarks of Referee #1:

This paper provides unique mechanistic insight into how the major human pathogen Streptococcus pneumonia controls oxidative damage to extracellular proteins, an important aspect of both the basic biology and the pathogenic cascade of this pathogen. In addition to elucidating a role for the Etrx proteins in host pathogenesis, the authors also provide compelling structural data to support their mechanistic arguments. Overall, the experiments are well designed and the conclusions are for the most part well supported by the data. One of the major shortcomings of the manuscript is the lack of elucidation of the functional consequences of their structural predictions. The authors make a number of very interesting conjectures into the structural aspects of the Etrx proteins **that they predict** would have major effects on the function of these proteins, however none of these predictions are tested experimentally. Such lines of investigation

would greatly enhance and provide more compelling evidence to support the predicted mechanisms underlying their structural modeling.

Response:

We thank the reviewer for the laudatory comments. The functional impact of the structural predictions has now been addressed and the experiments, their results and changes are described in the revised version. For details please see our responses to the major and minor points of referee#1.

Major Points

1. The biggest concern of this manuscript is the authors have substantial and intriguing structural data which they hypothesize plays major roles in the function of the Ertx proteins however there is no experimental confirmation of any of their predictions. The manuscript would be significantly enhanced by showing that a subset of the functional predictions discussed extensively in both the Results and Discussion could be shown experimentally. This could **be done via expression of the mutagenized Ertx proteins and assaying function** through any of the functional assays they discuss throughout the paper.

Response:

Following referee´s suggestion we have now determined the redox potential of Etrx1, Etrx2, MsrA2, and MsrB2, and we have also determined the kinetic parameters of methionine sulfoxide reductase activity of MsrA2 or MsrB2. The methionine sulfoxide reductase activity was measured in the presence of Etrx 1 and Etrx 2 protein, respectively. The results are now shown in Table 2 and in the new Figure 5. The reaction was performed at pH 7.4 in a mixture containing one of the Etrx proteins, NADPH, human thioredoxin reductase, and the reaction was started by the addition a MsrAB2 subunits as described in Material and Methods (due to space limitations in the Supporting Information). The determination of the redox potential is also included in Material and Methods. The results of the redox potential confirm our structural predictions and suggest that the electron flow occurs from Etrx1 or Etrx2 to MsrAB2 (methionine sulfoxide reductase AB2). The redox potentials of Etrx proteins and MsrAB2 subunits are: Etrx1: -191 ± 6 mV; Etrx 2: -282 ± 16.5 mV; MsrA2: -132.8 ± 5.9 mV; MsrB2: -120.9 ± 0.6 mV, and MsrAB2: -120.9 ± 0.6 mV.
These results are now shown in Figure 5 (new Figure). This suggested that electrons can be transported from the thioredoxin proteins Etrx1/Etrx2 to the

MsrAB2 (methionine sulfoxide reductase AB2) protein. To confirm this we have performed kinetic experiments and the results revealed that Etrx1 interacts preferentially with the MsrA2 subunit of MsrAB2 but not with MsrB2. In contrast, Etrx2 interacts with both subunits of SpMsrAB2, namely with MsrA2 and MsrB2. However, the efficiency to reduce MsrA2 was lower compared to the efficiency of Etrx1. These results are also shown in the new Figure 5 and Table 2.

The functional consequences of Etrx (CcdA) proteins for the electron transport to MsrAB2 were deciphered by determination of the cysteine-thiol redox state of MsrAB2. The pneumococcal protein-thiols in the reduced state were blocked by alkylation with AMS and the redox state of MsrAB2 was evaluated in diagonal 2D SDS-PAGE followed by immunoblotting with anti-MsrAB2 antibodies (new antibodies). Wild-type pneumococci, *ccdA*-mutants (new mutants that have been generated: Δ*ccdA1*, Δ*ccdA2*, and double knockout Δ*ccdA1*/Δ*ccdA2*), and *msrAB2*-mutant were used in these experiments. The results are also shown in the new Figure 5. Moreover, growth experiments were conducted in the presence of MetSO (methionine sulfoxide). The results clearly confirm the essential role of the extracellular thioredoxin system (new Fig S14).
The new mutants (*ccdA*- and *msrAB*-mutants) were also characterized on the molecular level. These data are included in Fig 1 and additional data are shown in the new Fig S11.

Please see also specific comment 6 of Referee #2.

2. Figure 1- based on the enolase it appears by Western that deletion of either Etrx protein results in the overexpression of the other- is this indeed the case? In panel C a number of the promoter and terminator sequences annotated in panel A are missing- the authors should include these in panel C for consistency.

Response:
Thanks for comment. We regret this inaccurateness; in the revised version we have now included the promotors and terminators in panel C of figure 1.

Since we show in the revised version also the phenotype of the *msrAB2*-mutant, the models of the *msrAB1*- and *msrAB2*-mutants are now included in panel C of Figure 1. In the diagonal 2 SDS-PAGE *ccdA*-mutants are used. These mutant constructs were also included in Fig 1. The molecular characterization is provided in Fig 1 and Fig S11.
Indeed, the expression of Etrx1 in the wild-type is low (as can be seen in the Immunoblot), while the lack of Etrx2 enhances the abundance of Etrx1 protein (anti-Etrx1: lane 3, upper panel in the immunoblots Figure 1C).
In addition, Etrx1 expression is enhanced in the ccdA1-mutant (Fig 1) and MsrAB2 expression is enhanced in the *ccdA1*- and *etrx1*-mutant (Fig S11). These data suggest that the mutant tries to compensate the loss-of-function of Etrx1. This is independent of the inserted antibiotic gene cassette (*ermB* or *aad9*) used and the direction of the cassette. The double knockout strains have another antibiotic gene cassette in *ccdA1* and *etrx1* than the single mutants.

3. Figure 2. It was slightly perplexing why the authors chose to show the FACS rather than to fractionate the cells and perform the Western Blot. This would give the readers an appreciation of the levels of the Etrx proteins in the cytoplasm, cell wall, and membrane factions respectively. The FACS does indicate that the Etrx proteins do reach the bacterial surface, though the case is stronger for Etrx2 than Etrx1. What proportion of the Etrx proteins that is surface/membrane associated is an important part of evidence to support the contention these proteins function to protect against extracellular oxidative damage.

Response:
Flow cytometry is a perfect method to demonstrate quantitative differences in the abundance of proteins on the surface of bacteria and cells. Therefore, we have selected this method to demonstrate the surface localization of our proteins and the absence of Etrx1 or Etrx2 in the mutants. However, the reviewer is correct that a fractionation of the bacterial compartments is able to show the levels of Etrx proteins and MsrAB2 in the cytoplasm and cell wall fraction (Gram-positive bacteria). The results of the new flow cytometric analysis for the MsrAB2 protein and our new immunoblots are now included in Figure 2 (Fig 2B).
Figure 2A shows the histograms (of the flow cytometry) for MsrAB2 and in Figure 2B we show the immunoblots. These data show the presence of MsrAB2 on the surface of wild-type pneumococci. The immunoblot analysis performed

after fractionation further demonstrates that the anti-MsrAB2 IgG raised in mice reacts also with the intracellular MsrAB1.
We have also tested the presence of Etrx1, Etrx2, and MsrAB2 in a mutant deficient for the Lgt protein (D39Δ*cps*Δ*lgt*). Lgt is the prolipoprotein diacylglycerol transferase and required for the lipoprotein biosynthetic process and anchoring to the cell surface (Voss et al., 2013). The results indicate that Etrx1 and Etrx2 but not MsrAB2 are lipoproteins. The MsrAB2 has one transmembrane domain (as shown in Figure 8) and this anchors the protein on the cell-surface of pneumococci. The sequence comparison of MsrAB2 and MsrAB1 shows that MsrAB1 lacks this transmembrane domain and also the flexible region N-terminally to the MsrA1 subunit is not present (see Figure S6). Therefore antibodies generated against MsrAb2 cross-react with teh intracelular MsrAB1.

Reference:

Voss, S., T. Hallstroem, M. Saleh, G. Burchhardt, T. Pribyl, B. Singh, K. Riesbeck, P.F. Zipfel, and S. Hammerschmidt (2013) The choline-binding protein PspC of *Streptococcus pneumoniae* interacts with the C-terminal heparin-binding domain of vitronectin. J Biol Chem. 288(22):15614-15627.

4. In Figure 2B- have the authors investigated whether hydrogen peroxide production is altered in the Ertx mutants? Is this effect only seen with hydrogen peroxide or is this observed with other oxygen radicals?

Response:
We have performed additional experiments and measured the growth and CFU in the presence of oxidative stress-inducing agents such as paraquat (stimulating superoxide production) and methionine sulfoxide (MetSO)
The survival (%) of pneumococci cultured in the presence of paraquat are added to Fig 2C and the growth curves of pneumococci cultured in the presence of MetSO are included in Fig S14 (new Figure) of the Supporting Information. The results show that the effect observed with H_2O_2 is also seen with paraquat and MetSO, further demonstrating the importance of the described extracellular thioredoxin system. Importantly, the phenotype of the *msrAB1*- and *msrAB2*-mutants, respectively, is also shown (for H_2O_2, paraquat, and MetSO).

5. Overall the murine pathogenesis experiments are well done and convincing. One potential explanation for this phenotype would be that the double mutant is defective at the initial stages of colonization of the nasopharynx, either due to more effective initial clearance by the host or defective bacterial adherence/replication in this tissue. It might be expected that colonization of the airways would be pronouncedly different in the double mutant due to increased oxygen in this host niche. This would be an interesting clarification into the precise roles of these proteins during pathogenesis.

Response:

Similar to earlier studies characterizing the effect of pneumococcal adhesins, we have employed the pneumococcal carriage infection model (Jensch et al., 2010). Mice were intranasally infected with 1 x 10^6 pneumococci and bacteria were recovered (after 24, 72, and 120 hrs) from the nasopharynx and bronchoalveolar avage. The CFU were calculated by plating the recovered bacteria on blood agar plates. This *in vivo* infection, with an infection dose of 1 x 10^6 bacteria, has been done with wild-type D39lux and isogenic mutants deficient in Etrx1, Etrx2, Etrx1/2, and MsrAB2 (D39*lux* mutants).

The results showed at all time points a significant reduction of nasopharyngeal carriage for the double mutant D39*lux*Δ*etrx1*Δ*etrx2* compared to the wild-type (Fig S16). The other *etrx*- or *msrAB2*-mutants were not significantly different in nasopharyngeal carriage from the isogenic wild-type (Fig S16). However, the *msrAB2*-mutant showed immediately at 24 h post-infection a significantly reduced number of CFU (Fig S16). (see also page 13)

The results are shown in the new Figure S16 of the Supporting Information

Reference:

Jensch, I., G. Gamez, M. Rothe, S. Ebert, M. Fulde, D. Somplatzki, S. Bergmann, L. Petruschka, M. Rohde, R. Nau and S. Hammerschmidt (2010) PavB is a surface-exposed adhesin of *Streptococcus pneumoniae* contributing to nasopharyngeal colonization and airways infections. Mol Microbiol 77: 22-43

6. In Figure 7 the model for the most part is well done and provides the reader with how the various proteins are interacting. The one thing the authors might consider here is coloring the arrows either white or gray based on whether the interactions are based on whether the interactions have been demonstrated experimentally or are presumed to occur.

Response:
The Figure 8 (formerly Fig 7) has been modified accordingly with white arrows for presume interactions and grey arrows for those demonstrated experimentally. The experimentally calculated values for the redox potential of each protein are now included in the figure.

Minor Points

1. The manuscript contains numerous typographical errors- page 3 line 4 e.g. should be approximately, OH should be OH-, page 4 line 18 compartiments should be compartments, Table1 should have a space, page 20 035 should read 0.35, in the results on page 11 phagocytozed should read phagocytosed,

Response:
The typographical errors have been corrected.

2. The authors indicate that the mutations did not effect production of SpMsrAB2 by Western, this should be included in the Supplementary material if possible.

Response:
We have included the immunoblots in Fig S11, since we now also demonstrate the effect of MsrAB2 deficiency on virulence and susceptibility against oxidative stress.

3. One page 9 the authors state that the Proline reside found in the Ertx proteins 5 residues past the CxxC motif is not found in other thioredoxin like proteins, but the Figure shows that the same proline is found in ResA?

Response:
In the manuscript we mentioned "that a further proline residue found in Etrx1 and Etrx2, respectively five positions after the CXXC motif, Pro92 and Pro89 is not conserved in other thioredoxin-like proteins ….". This is true as far as this proline is not found in all the thioredoxin proteins. However, in order to avoid any confusion we have deleted that sentence in the revised version.

4. On page 13 in the Discussion the authors claim the lost of Ertx1/2 attenuates pneumococcus in the pneumonia and sepsis models but in the results only the pneumonia model shows a statistically significant difference.

Response:
This was correct in the results (see also Figure 5G and 5H now) and we have corrected this in the discussion.

5. In the methods the methods the bacteria were cultured both in THY and CDM media for the oxidative stress experiments but only the THY data is shown.

Response:
This has been corrected in the revised version. All the data are now shown in Fig S15.

6. For Table 1 it would be helpful to have the PDB# included for the structures.

Response:
In the revised version Table 1 contains the PDB codes for each structure

7. For the quantification of the luminescence, is this per mouse or was this for a selected area of the mouse- was unclear if there should be a unit of area included in this measurement.

Response:
We have used and published this method several times and the references are cited. Briefly, the bioluminescence of each individual mouse is measured (not a defined area) and each individual box and whisker plot shows the median, lower quartile (to the minimum) and upper quartile (to the maximum) bioluminescence flux (photons/sec) for one group of mice infected with one bacterial strain and measured at the indicated time point.

<u>Referee #2</u>

Remarks:

The article by Bartual et al. reports the characterization of two surface -exposed thioredoxin proteins, Etrx1 and Etrx2, from Streptococcus pneumoniae. These proteins are encoded by two operons that also include a gene coding for a homolog of ccdA. CcdA is a membrane protein that transports reducing equivalents across the membrane and transfers them to extracellular proteins involved in reductive pathways. The etrx1 operon also includes a putative extracellular methionine sulfoxide reductase (SpMsrAB2). Loss of function of Etrx1 and Etrx2 together results in a dramatic decrease in virulence and an enhanced uptake by macrophages. The authors propose that the lack of Etrx1 and Etrx2 results in the inactivation of SpMsrAB2, which would partially explain the observed phenotypes. The structures of both proteins are also presented in the paper.

Overall the work is very interesting, but the data presented are too preliminary. There is too much speculation on the function of Etrx1 and Etrx2 in the defense against oxidative stress and in the reduction of SpMsrAB2.

Response:

Referee #2 asked for additional experiments, aimed to confirm the function of Etrx1 and Etrx2. We have addressed this aspect and provide new and additional experiments confirming the proposed reductive pathway. We have now, for example, determined the redox potential of Etrx proteins and MsrAB2 subunits. We further show the kinetics of methionine sulfoxide reductase activity in the presence of Etrx1 and Etrx2, respectively. All the new experiments, their results and Figures are included in the revised version of our manuscript.
Please see below for our point-by-point response to the specific comments of referee #2.

Specific comments:

1-what is the evidence that SpMsrAB2 is surface exposed ?

Response:

The phenotype of the *msrAB2*-mutant is included in the revised version. We have, therefore, also included data showing the surface-exposure of MsrAB2. The presence of MsrAB2 on the pneumococcal cell-surface has been shown by flow cytometry and immunoblot analysis (see Figure 2). In

Figure 2A histograms are shown for Etrx1, Etrx2, and MsrAB2 and in Figure 2B we show immunoblots, indicating that the Etrx proteins are surface-exposed lipoproteins and that MsrAB2 is also located on the pneumococcal cell-surface. The annotation in the gene locus of TIGR4 is not correct since a TTG was (mis-) used as a start codon. Upstream of this sequence the real start codon was identified (see sequences below and Supporting Information).

```
                   >SP_0660long gene

start              ATGAATGATAAGTTAAAAATCTTCTTGTTGCTAGGAGTATTTTTTCTAGCCATAACCGGTTTCTATGTTCT
TIGR4 Annotation TTG  ATTGATACGAAATGCAGGGCAGACAGATGCCTCGCAAATTGAAAAGGCGGCAGTTAGCCAAGGAGGAAAAG
                   CAGTGAAAAAAACAGAAATTAGTAAAGACGCAGACTTGCACGAAATTTATCTAGCTGGAGGTTGTTTCTGG
                   GGAGTGGAGGAATATTTCTCACGTGTTCCCGGGGTGACGGATGCCGTTTCAGGCTATGCAAATGGTAGAGG
                   AGAAACAACCAAGTACGAATTGATTAACCAAACAGGTCATGCAGAAACCGTCCATGTCACCTATGATGCCA
                   AGCAAATTTCTCTCAAGGAAATCCTGCTTCACTATTTCCGCATTATCAATCCAACCAGCAAAAATAAACAA
                   GGAAATGATGTGGGGACCCAGTACCGTACTGGTGTTTATTACACAGATGACAAGGATTTGGAAGTGATTAA
                   CCAAGTCTTTGATGAGGTGGCTAAGAAATACGATCAACCTCTAGCAGTTGAAAAGGAAAACTTGAAGAATT
                   TTGTGGTGGCTGAGGATTACCATCAAGACTATCTCAAGAAAAATCCAAATGGCTACTGCCATATCAATGTT
                   AATCAGGCGGCCTATCCTGTCATTGATGCCAGCAAATATCCAAAACCAAGTGATGAGGAATTGAAAAAGAC
                   CCTGTCACCTGAGGAGTATGCAGTTACCCAGGAAAATCAAACAGAACGAGCTTTCTCAAACCGTTACTGGG
                   ATAAATTTGAATCCGGTATCTATGTGGATATAGCAACTGGGGAACCTCTCTTTTCATCAAAAGACAAATTT
                   GAGTCTGGTTGTGGCTGGCCTAGTTTTACCCAACCCATCAGTCCAGATGTTGTCACCTACAAGGAAGATAA
                   GTCCTACAATATGACGCGTATGGAAGTGCGGAGCCGAGTAGGAGATTCTCACCTTGGGCATGTCTTTACGG
                   ATGGTCCACAGGACAAGGGCGGCTTACGTTACTGTATCAATAGCCTCTCTATCCGCTTTATTCCCAAAGAC
                   CAAATGGAAGAAAAAGGCTACGCTTATTTACTAGATTATGTTGATTAA

                   >SP_0660long protein
                   MNDKLKIFLLLGVFFLAITGFYVLLIRNAGQTDASQIEKAAVSQGGKAVKKTEISKDADLHEIYLAGGCFW
                   GVEEYFSRVPGVTDAVSGYANGRGETTKYELINQTGHAETVHVTYDAKQISLKEILLHYFRIINPTSKNKQ
                   GNDVGTQYRTGVYYTDDKDLEVINQVFDEVAKKYDQPLAVEKENLKNFVVAEDYHQDYLKKNPNGYCHINV
                   NQAAYPVIDASKYPKPSDEELKKTLSPEEYAVTQENQTERAFSNRYWDKFESGIYVDIATGEPLFSSKDKF
                   ESGCGWPSFTQPISPDVVTYKEDKSYNMTRMEVRSRVGDSHLGHVFTDGPQDKGGLRYCINSLSIRFIPKD
                   QMEEKGYAYLLDYVD
```

We have also tested the presence of Etrx1, Etrx2, and MsrAB2 in a mutant deficient for the Lgt protein (D39Δ*cps*Δ*lgt*). Lgt is the prolipoprotein diacylglycerol transferase and required for the lipoprotein biosynthetic process and anchoring to the cell surface (Voss et al., 2013). The results indicate that Etrx1 and Etrx2 but not MsrAB2 are lipoproteins. The MsrAB2 has one transmembrane domain (as shown in Figure 8) and this anchors the protein on the cell-surface of pneumococci. The sequence comparison of MsrAB2 and MsrAB1 shows that MsrAB1 lacks this transmembrane domain and also the flexible region N-terminally to the MsrA1 subunit (see Figure S6).

Please see also our response to point 3 of Referee #1.

Reference:

Voss, S., T. Hallstroem, M. Saleh, G. Burchhardt, T. Pribyl, B. Singh, K. Riesbeck, P.F. Zipfel, and S. Hammerschmidt (2013) The choline-binding protein PspC of *Streptococcus pneumoniae* interacts with the C-terminal heparin-binding domain of vitronectin. J Biol Chem. 288(22):15614-15627.

2-the authors present data (Fig 2A) suggesting that Etrx1 and Etrx2 are surface exposed. Because this is a really central result, I think that it must be confirmed using another method, such as a protease digestion assay. Moreover, more explanation should be provided regarding the results from the flow cytometric analysis.

Response:
We have addressed this point as already mentioned above (point 1 of Referee #2 and point 3 of Referee #1). Flow cytometry is a perfect method to demonstrate quantitative differences in the abundance of proteins on the surface of bacteria and cells. Since we have now included the immunoblots we have partially re-written the sentences addressing the surface-exposure of Etrx proteins and MsrAB2 (page 6-7)

3-page 7: The authors claim that the deletion of etrx1 has no impact on the production of SpMsrAB2 but they do not show the data. The data must be shown in the supplementary material.

Response:
The *msrAB2*-mutant is now part of our study and therefore we show now the expression of MsrAB2 in the various mutants including the *etrx1*-mutant. The immunoblots are included in Figure 1C and Figure S11.

4-Why did the authors use a bioluminescent strain for plating and CFU counting?

Response:
We have used in most of the experiments shown in the paper the identical set of strains/mutants. However, we have performed e.g. also phagocytosis experiments with nonencapsulated D39 lacking bioluminescence and the results were similar to data presented in the manuscript. The bioluminescent strains were used in all assays, since bioimaging using the IVIS Spectrum is only feasible with these bacterial strains.

5-What is the impact of cps deletion on the strain. This should be briefly discussed.

Response:
We have published the impact of capsule-deficiency on adherence and phagocytosis in 2005 (Hammerschmidt et al., 2005) and 2009 (Noske et al., 2009). Encapsulated strains such as the D39 are perfectly suited for *in vivo* infections but adherence to professional cells is impaired. Therefore, *in vivo* infections are carried out with wild-type pneumococci producing CPS, while *in vitro* infections are conducted with the isogenic *cps*-mutants.
Since the results and impact of CPS-deficiency are published several times (also by other groups), we have not addressed this point in detail.

References:
Noske, N., U. Kämmerer, M. Rohde, and **S. Hammerschmidt** (2009) Pneumococcal interaction with human dendritic cells: phagocytosis, survival and induced adaptive immune response is manipulated by PavA. J. Immunology 183: 1952-1963.

6-The **functional characterization of Etrx1 and Etrx2 is particularly preliminary**. This is clearly my major comment :

a-What is the evidence that Etrx1 and Etrx2 function in a reductive pathway ? The in vivo redox state of these proteins should be determined using classical AMS-trapping assays.

Response:

Referee #1 also suggested to perform additional experiments. Please find below our response to the major point of referee #1 and to point 6a of referee #2.

Following referee´s suggestion we have now determined the redox potential of Etrx1, Etrx2, MsrA2, and MsrB2, and we have also determined the kinetic parameters of methionine sulfoxide reductase activity of MsrA2 or MsrB2. The methionine sulfoxide reductase activity was measured in the presence of Etrx 1

and Etrx 2 protein, respectively. The results are shown in Table 2 and in the new Figure 5. The reaction was performed at pH 7.4 in a mixture containing one of the Etrx proteins, NADPH, human thioredoxin reductase, and the reaction was started by the addition of MsrAB2 subunits as described in Material and Methods (due to space limitations in the Supporting Information). The determination of the redox potential is also included in Material and Methods. The results of the redox potential confirm our structural predictions and suggest that the electron flow occurs from Etrx1 or Etrx2 to MsrAB2 (methionine sulfoxide reductase AB2). The redox potentials of Etrx proteins and MsrAB2 subunits are: Etrx1: -191 ± 6 mV; Etrx 2: -282 ± 16.5 mV; MsrA2: -132.8 ± 5.9 mV; MsrB2: -120.9 ± 0.6 mV, and MsrAB2: -120.9 ± 0.6 mV.
These results are now shown in Figure 5 (new Figure). This suggested that electrons can be transported from the thioredoxin proteins Etrx1/Etrx2 to the MsrAB2 (methionine sulfoxide reductase AB2) protein. To confirm this we have performed kinetic experiments and the results revealed that Etrx1 interacts preferentially with the MsrA2 subunit of MsrAB2 but not with MsrB2. In contrast, Etrx2 interacts with both subunits of SpMsrAB2, namely with MsrA2 and MsrB2. However, the efficiency to reduce MsrA2 was lower compared to the efficiency of Etrx1. These results are also shown in the new Figure 5 and Table 2.

The functional consequences of Etrx (CcdA) proteins for the electron transport to MsrAB2 were deciphered by determination of the cysteine-thiol redox state of MsrAB2. The pneumococcal protein-thiols in the reduced state were blocked by alkylation with AMS and the redox state of MsrAB2 was evaluated in diagonal 2D SDS-PAGE followed by immunoblotting with anti-MsrAB2 antibodies (new antibodies). Wild-type pneumococci, *ccdA*-mutants (new mutants that have been generated: Δ*ccdA1*, Δ*ccdA2*, and double knockout Δ*ccdA1*/Δ*ccdA2*) and *msrAB2*-mutant were used in these experiments. The results are also shown in the new Figure 5. Moreover, growth experiments were conducted in the presence of MetSO (methionine sulfoxide). The results clearly confirm the essential role of the extracellular thioredoxin system (new Fig S14).
The new mutants (*ccdA*- and *msrAB*-mutants) were also characterized on the molecular level. These data are included in Fig 1 and additional data are shown in the new Fig S11.

b-what is the evidence (other than the presence in the same operon) that Etrx1 is reduced by CcdA1 and Etrx2 by CcdA2. Can CcdA1 reduce ETrx2 as well?

Response:
This is an interesting question, but at this stage we can´t provide an appropriate answer. We don´t know the distribution of CcdA1/CcdA2 on the pneumococcal surface and whether there are protein complexes formed on the pneumococcal cell-surface. Since the homology is high one can assume that both CcdA proteins are able to reduce Etrx1 and Etrx2. However, the results of the *ccdA* double mutant in the diagonal 2D SDS-PAGE suggests that both CcdA proteins are able to reduce Etrx1 and Etrx2.

c-A key point : the authors claim that Etrx1 and Etrx2 can both reduce SpMsrAB2. They must show it in vitro and, possibly, in vivo (by monitoring SpMsrAB2 redox state in the single and double mutants). I don't agree when they write that "SpMsrAB2 is the only redox partner for both Etrx proteins" (page 13).

Response:
We took this specific point to heart by performing different experiments. Please see our comments and new results on your point 6a.
The results have been included in Fig 8 (formerly Fig 7)

7-What is the phenotype of a ∆ MsrAB2 mutant ? is it similar to the phenotype of a etrx1etrx2 mutant ?

Response:
We have performed mouse infection and phagocytosis experiments with the *msrAB*-mutants. The results revealed a phenotype similar to the double-KO *etrx1/2*.
The results are included in Figure 6 (new panel 6E, 6F, and 6H (formerly Fig 5)) and Figure 7 (formerly Fig 6). The results are also mentioned in the text on page 13 and 14 of the revised version.
In addition the carriage rates in mice of mutants (*etrx* and *msrAB2*) was compared to the isogenic parental strain D39lux. These data are shown in Figure S16 of the Supporting Information.

Please see also major point 5 of Referee #1.

8-I disagree with the authors when they conclude that the survival of the etrx1 or etrx2 mutant is not significantly affected by H_2O_2 (Fig. 2B).

Response:
The reviewer is correct and we have corrected this.

9-page 13: the authors write that "the deficiency of both Etrx proteins significantly enhanced oxidative damage to proteins": **maybe**, but they need to provide evidence to support this conclusion.

Response:
The reviewer is correct. Currently we can only hypothesize that damage to proteins is enhanced in the absence of both Etrx proteins, but we have no experimental evidences. Therefore, we have re-written this statement.

In conclusion, this manuscript has the potential to become a very nice paper, but more work must be done to clearly and unambiguously characterize the function of Etrx1, Etrx2 and SpMsrAB2.

Response:
Thanks to the reviewer for this laudatory comment.

2nd Editorial Decision 10 July 2013

Thank you for the submission of your revised manuscript to EMBO Molecular Medicine. We have now received the enclosed reports from the referees that were asked to re-assess it. As you will see, while one referee is now fully supportive, the second referee however, still points to some issues that have to be addressed in the final version of this manuscript.

Referee 2 comments on the new figure 5 and all issues raised have to be satisfactorily addressed. I would like to take this opportunity to mention that we now encourage the publication of source data, particularly for electrophoretic gels and blots, with the aim of making primary data more accessible and transparent to the reader. Would you be willing to provide a single PDF file comprising the original, uncropped and unprocessed scans of all or key gels used in the figures (and especially figure 5)? These should be labeled with the appropriate figure/panel number, and should have molecular weight markers; further annotation could be useful but is not essential. This PDF will be published online with the article as a supplementary "Source Data" file. If you have any questions regarding this just contact me.

We agree with Referee 2 that the structure and clarity of the manuscript need to be improved to increase the impact and readability of your work.

We note that the quality of the figure 4 is a bit low. Please provide higher resolution version, and check to make sure that text/line-art remains clear even when zooming in. You may find that saving the images as EPS or PDF will better preserve the text and line-art resolution. If this does not help, you may need to remake the figures in a quality vector graphics program like Illustrator or the free opensource, alternative Inkscape.

I look forward to seeing a revised form of your manuscript as soon as possible.

***** Reviewer's comments *****

Referee #1 (Comments on Novelty/Model System):

Both the technical quality and novelty of this system have been much improved in this revision. It is a very interesting system from a molecular microbiology standpoint and provides important insights into how the pneumococcus deals with extracellular oxidants. I noted the medical impact only at medium, as this would not directly impact medical practices but it provides a very interesting route for new routes for novel therapeutics.

Referee #1 (Remarks):

In this revision the authors have addressed many of the reviewer's concerns and have responded with additional experiments that add substantially to the mechanisms underlying the structural predictions. The text has also be clarified and improved in many areas, providing greater context of the experimental outcomes to a general readership. The experimental design is well planned and the conclusions are well supported by the data presented. The topic is quite interesting and the manuscript well written to be accessible to a general audience.

I have no additional suggestions to further strengthen the manuscript and my concerns have all been addressed in a sufficient manner.

Referee #2 (Comments on Novelty/Model System):

I still believe that this paper has the potential to become a very interesting one, but I have to admit that I am disappointed by the revised version. The authors added the results of many experiments, but, in its current version, the paper remains vague. Too many questions remain partially answered. The paper is also particularly poorly presented and should be thoroughly reorganized and edited.

© EMBO

Referee #2 (Remarks):

This paper by Saleh et al. reports the characterization of two surface-exposed thioredoxin proteins, Etrx1 and Etrx2, from *Streptococcus pneumoniae*. These two proteins are proposed to deliver electrons to MsrAB2, a surface exposed methionine sulfoxide reductase. Loss of function of Etrx1 and Etrx2 results in a dramatic decrease in virulence and an enhanced uptake by macrophages.

The authors have taken the comments made by the reviewers into account: additional experiments have been performed and new data have been included. However, although I remain convinced that the data reported by this manuscript are interesting and that the Etrx/Msr system is worth investigating, I am not entirely satisfied with this revised version. My major problem is that the new data that have been added to further characterize the functional relationship between Etrx proteins and MsrAB2 are not fully convincing (see specific comments). Another major concern is the lack of clarity of the new manuscript, which seems to have been prepared hastily. The authors must carefully edit the manuscript and improve its structure and clarity. They should also pay a particular attention to introducing protein names and techniques, adding more references to the figures and prepare more detailed figure legends.

Major comments:

Page 11, Fig. 5A/B: the data shown for the redox potential of Etrx2 are not really convincing. In the lower panel (AMS experiments), why is there more reduced MsrA2/B2 than oxidized MsrA2/B2? Were some disulfide-linked oligomers present in the gel but cut to prepare the figure? The units are also missing for the X axis of Fig. 5B.

Page 12, Fig. 5C: the authors state that the redox state of MsrAB2 "in the different etrx- and ccdA mutants was analyzed in vivo" (Fig 5C). I only see the ccdA mutants and the msrAB mutant in this figure but not the Etrx mutants. Furthermore, this figure is not well explained: the choice for the diagonal gel technique should be discussed (and the technique explained for the general audience). This figure, as presented and as explained, is not convincing. The functional relationship between ccdAs, eTrxs and MsrAB2 is only partially clarified.

Minor comments:

Page 7: the data from the immunoblots are shown in Fig 1D and not 1C.

Page 7: a reference to Fig. 2B should be added earlier in the paragraph. Controls corresponding to cytoplasmic and membrane proteins are missing in Fig. 2B. Moreover, the data indicate that Etrx1 and Etrx2 are membrane proteins but not that they are surface exposed. I understand that lipoproteins do not face the cytoplasm, but it would have been nice to unambiguously confirm that Etrx1/2 are surface exposed using experiments such as protease digestion experiments.

Page 7: 90 min to paraquat

Page 8: the authors state that inactivation of either Etrx1 and Etrx2 or of SpMsrAB2 renders pneumococci similarly sensitive to oxidative stress. I think that they need to be more careful as the double mutant is clearly more sensitive than the msrAB2 mutant.

Page 12: how do the authors explain the toxicity of MetSO on the growth of the msrAB2 mutant? I may have missed it, but I do not think it is discussed.

2nd Revision - authors' response 15 August 2013

Referee #1 (Remarks):

I have no additional suggestions to further strengthen the manuscript and my concerns have all been addressed in a sufficient manner.

Response:

We thank the reviewer for her/his comment.

Referee #2 (Remarks):

This paper by Saleh et al. reports the characterization of two surface-exposed thioredoxin proteins, Etrx1 and Etrx2, from Streptococcus pneumoniae. These two proteins are proposed to deliver electrons to MsrAB2, a surface exposed methionine sulfoxide reductase. Loss of function of Etrx1 and Etrx2 results in a dramatic decrease in virulence and an enhanced uptake by macrophages.

The authors have taken the comments made by the reviewers into account: additional experiments have been performed and new data have been included. However, although I remain convinced that the data reported by this manuscript are interesting and that the Etrx/Msr system is worth investigating, I am not entirely satisfied with this revised version. My major problem is that the new data that have been added to further characterize the functional relationship between Etrx proteins and MsrAB2 are not fully convincing (see specific comments). Another major concern is the lack of clarity of the new manuscript, which seems to have been prepared hastily. The authors must carefully edit the manuscript and improve its structure and clarity. They should also pay a particular attention to introducing protein names and techniques, adding more references to the figures and prepare more detailed figure legends.

Response:

As mentioned by the reviewer we have added the results of many experiments.

Most of these experiments were requested by Reviewer 1 and Reviewer 2 and were extremely helpful in improving this study. In our first revised version (R1) we had also included other additional experiments and data supporting all of our hypotheses mentioned in the original version of our manuscript. In addition to the experiments suggested by the reviewers we have used an *lgt*-mutant to demonstrate that Etrx1 and Etrx2 are lipoproteins and surface-displayed. The *lgt*-mutant lacks the diacylglyceryl transferase activity and thus, the lipoprotein maturation is impaired. We have recently submitted a proteome-based study demonstrating that the *lgt*-mutant lacks the Etrx-proteins on the pneumococcal cell surface (Pribyl *et al*., submitted). In this study a biotinylation approach followed by 1D-SDS-PAGE and liquid chromatography-mass spectrometry (1D-GeLC-MS) has been applied to identify surface proteins of *S. pneumoniae* D39. The results identified Etrx1 and Etrx2 as surface-displayed lipoproteins when using the wild type, while the *lgt*-mutant lacks both Etrx proteins.

Moreover, the data of our flow cytometric analysis and our immunoblots indicate that Etrx1, Etrx2, and *Sp*MsrAB2 are surface-displayed proteins. Flow cytometry is a method broadly used to identify surface-displayed antigens on eukaryotic and prokaryotic cells.

Taken together all the data clearly demonstrate that Etrx1, Etrx2, and *Sp*MsrAB2 are surface-displayed proteins (Fig. 2A and Fig. 2B). However, we have now also added the data of trypsin and pronase E treated pneumococci, which confirm that Etrx1 and Etrx2 are surface-displayed proteins of *S. pneumoniae* (new Fig S11). This treatment is generally used to demonstrate e.g. that bacterial

adhesins for host proteins are of proteinaceous nature. We have used this approach several times but prefer now flow cytometric and genetic approaches, as they are more precise.

We have also determined the redox potential and the kinetic parameters of Etrx1, Etrx2, MsrA2, and MsrB2, although this was not recommended by the reviewers. The data confirm that the electrons can be transferred from Etrx1 to MsrA2 but not MsrB2, while Etrx2 is able to regenerate oxidized MsrA2 and MsrB2 (improved Fig. 5A and 5B).

We apologize for the inaccurateness of Fig. 5B, which lacked the x-axis. The quality of Fig. 5A and 5B has now been improved, Figure 5C contains new data.

We have again carefully edited the manuscript and paid particular attention introducing protein names and techniques. Some parts were re-structured as suggested by Reviewer 2 in order to explain in more detail the used approaches and results. The figure legends have been corrected and improved. The figure legend of Figure 5C has been re-written since we have included new data and controls. For details please see our responses to the major comments.

The major changes:

- enolase is included as a control protein in Figure 2B
- the proteolytic digest of pneumococci was performed to confirm that Etrx1 and Etrx2 are surface-exposed proteins– results are shown in Fig S11
- Figure 4 is provided as high quality file (tif format)
- Figure 5 is improved – the quality (resolution) of Fig 5A and 5B has been improved
- additional controls have been included in Fig 5C – this figure has been re-structured
- the diagonal non-reducing/reducing SDS-PAGE is explained in more detail and the figure legend (Fig 5C) has been re-written
- the introduction has been improved according to the suggestion of Reviewer 2
- the source data of Fig 1D, 2B, and Fig 5C are provided

Major comments:

Page 11, Fig. 5A/B: the data shown for the redox potential of Etrx2 are not really convincing. In the lower panel (AMS experiments), why is there more reduced MsrA2/B2 than oxidized MsrA2/B2? Were some disulfide-linked oligomers present in the gel but cut to prepare the figure? The units are also missing for the X axis of Fig. 5B.

Response:

We apologize that the x-axis in Fig. 5B was not displayed. This has been corrected. Full scans of the (stain-free visualized, see manufacturer Biorad) gels are now provided as supplementary material (source data file). These scans clearly demonstrate the absence of any additional bands. In fact, the scans display only those bands corresponding to the two reduced/dithiol and oxidized/disulfide protein bands. There were no additional disulfide-linked oligomers present in the gel which had been cut out to prepare the figure. Please keep in mind that purified (untagged) MsrA or MsrB proteins were used in this assay.

All the figures and the data were prepared according to the rules of good scientific practice. We did not take special care to load exactly the same amount of purified recombinant protein per lane, since in every lane the ratio between reduced and oxidized band were determined individually and used for the calculation of the potentials. The units for both Fig. 5A and B [mV] are clearly included in the common description of the X-axis.

Page 12, Fig. 5C: the authors state that the redox state of MsrAB2 "in the different etrx- and ccdA mutants was analyzed in vivo" (Fig 5C). I only see the ccdA mutants and the msrAB mutant in this figure but not the Etrx mutants. Furthermore, this figure is not well explained: the choice for the diagonal gel technique should be discussed (and the technique explained for the general audience). This figure, as presented and as explained, is not convincing. The functional relationship between ccdAs, eTrxs and MsrAB2 is only partially clarified.

Response:

We thank the reviewer for this important comment and have improved the text (page 11-12) and figure legend (page 34-35) to explain the functional relationship between CcdAs, Etrx1/2 and MsrAB2 in more detail. Figure 5 clearly demonstrates that electrons can be transferred from Etrx1 to MsrA2 but not Etrx2. In contrast, Etrx2 is able to regenerate oxidized MsrA2 and MsrB2 (Fig. 5A and 5B). In Figure 5C the *in vivo* data are presented, which show that the lack of both CcdAs (the transmembrane proteins accepting electrons from an intracellular donor) in the double mutant D39Δ*ccdA1*Δ*ccdA2* impairs the regeneration of oxidized MsrAB2. This is shown in panel d and e of Figure 5C.

We have also included new controls and the mutant D39Δ*etrx1*Δ*etrx2* (panel h of Figure 5C). The immunoblots of the *msrAB1*- and *msrAB2*-mutant were repeated and now developed only with the anti-MsrAB specific antiserum to display only the *Sp*MsrAB proteins and their redox state. The diagonal immunoblots of these controls (panel e to h) simplify the assignment of the proteins to the corresponding spots.

The non-reducing/reducing SDS-PAGE (diagonal assay) and the results are now explained in more detail (page 11-12).

The figure was re-structured, the figure legend was re-written (page 34-35), and new references are provided.

Due to the additional controls and changes in the text we are now convinced that the functional relationship between CcdAs, Etrxs, and SpMsrAB2 is clarified.

Minor comments:

Page 7: the data from the immunoblots are shown in Fig 1D and not 1C.

Response:

This has been corrected.

Page 7: a reference to Fig. 2B should be added earlier in the paragraph. Controls corresponding to cytoplasmic and membrane proteins are missing in Fig. 2B. Moreover, the data indicate that Etrx1 and Etrx2 are membrane proteins but not that they are surface exposed. I understand that lipoproteins do not face the cytoplasm, but it would have been nice to unambiguously confirm that Etrx1/2 are surface exposed using experiments such as protease digestion experiments.

Response:

We have added a further reference earlier in the paragraph, explained the *lgt*-mutant in more detail (page 6) and included the control protein (enolase as cytoplasmic protein) in Fig. 2B.

As mentioned above the data of our flow cytometric analyses (Fig. 2A) and our immunoblots (Fig. 2B) clearly indicate that Etrx1, Etrx2, and *Sp*MsrAB2 are surface-displayed proteins. Flow cytometry is a method broadly used to identify surface-displayed antigens on eukaryotic and prokaryotic cells.

Taken together all the data clearly demonstrate that Etrx1, Etrx2, and *Sp*MsrAB2 are indeed surface-displayed proteins (Fig. 2A and Fig. 2B). However, we have added the data of trypsin and pronase E treated pneumococci, which confirm that Etrx1 and Etrx2 are surface-displayed proteins of *S. pneumoniae* (new Fig. S11).

Page 7: 90 min to paraquat

Response:

This has been corrected.

Page 8: the authors state that inactivation of either Etrx1 and Etrx2 or of SpMsrAB2 renders pneumococci similarly sensitive to oxidative stress. I think that they need to be more careful as the double mutant is clearly more sensitive than the msrAB2 mutant.

Response:

The reviewer is correct and the sentence on page 8 has been re-written (page 8, 1st paragraph, last sentence)

Page 12: how do the authors explain the toxicity of MetSO on the growth of the msrAB2 mutant? I may have missed it, but I do not think it is discussed.

Response:

The reviewer is correct and we discussed this point (page 16/17).

3rd Editorial Decision 10 September 2013

Please find enclosed the final report on your manuscript. We are pleased to inform you that your manuscript is accepted for publication and is now being sent to our publisher to be included in the next available issue of EMBO Molecular Medicine.

***** Reviewer's comments *****

Referee #2 (Remarks):

I have read the revised version of the manuscript. I find it much improved compared to the previous one. The authors have taken the comments into account and modified the manuscript accordingly.

© EMBO
