# Supplementary material for: Molecular architecture of Streptococcus pneumoniae surface thioredoxin-fold lipoproteins crucial for extracellular oxidative stress resistance and maintenance of virulence
Source: EMBO Mol Med. 2013 Oct 18;5(12):1852–70. doi: 10.1002/emmm.201202435 (PMC3914529; doi:10.1002/emmm.201202435)
Supplement: Supplementary file 2 [file emmm0005-1852-sd2.pdf]

Supplementary Information

Molecular architecture of *Streptococcus pneumoniae* surface thioredoxin-fold lipoproteins crucial for extracellular oxidative stress resistance and maintenance of virulence

Malek Saleh,[1,5] Sergio G. Bartual,[2,5] Mohammed R. Abdullah,[1,5] Inga Jensch,[1] Tauseef M. Asmat,[1] Lothar Petruschka,[1] Thomas Pribyl,[1] Manuela Gellert, [3] Christopher H. Lillig,[3] Haike Antelmann,[4] Juan A. Hermoso, [2*] and Sven Hammerschmidt [1*]

(1) Department Genetics of Microorganisms, Interfaculty Institute for Genetics and Functional Genomics, Ernst Moritz Arndt University of Greifswald, D-17487 Greifswald, Germany

(2) Departamento de Cristalografía y Biología Estructural, Instituto de Química-Física "Rocasolano", CSIC, Serrano 119, 28006-Madrid, Spain

(3) Institute for Medical Biochemistry and Molecular Biology, University Medicine, Ernst Moritz Arndt University of Greifswald, D-17487 Greifswald, Germany.

(4) Institute for Microbiology, Ernst Moritz Arndt University of Greifswald, D-17487, Greifswald, Germany.

(5) These authors contributed equally to this work

<u>Contents:</u>

Supplementary Materials and Methods **Page 2-15**
Supplementary References **Page 15-17**

Supplementary Figures **Page 18-35**
Figure S1
Figure S2
Figure S3
Figure S4
Figure S5
Figure S6
Figure S7
Figure S8
Figure S9
Figure S10
Figure S11
Figure S12
Figure S13
Figure S14
Figure S15
Figure S16
Figure S17
Figure S18

Supplemental Movie 1 **Page 36**
Supplementary Tables **Page 37-40**

Table S1
Table S2

Supplementary Materials and Methods

Bioinformatic identification and molecular analysis of the lipoproteins Etrx1 and Etrx2

A bioinformatic approach based on the LocateP dataset (http://www.cmbi.ru.nl/locatep-db/cgi-bin/locatepdb.py), protein sorting tools such as LipoP and SignalP (http://www.cbs.dtu.dk/services/), PSORTdb database of protein subcellular localizations, http://db.psort.org/) and DOLOP database of bacterial lipoproteins (http://www.mrc-lmb.cam.ac.uk/genomes/dolop/) was employed to identify surface-exposed lipoproteins in pneumococcal genome sequences deposited in the CMR database ((http://cmr.jcvi.org/tigr-scripts/CMR/shared/Genomes.cgi) of the J. Craig Venter Institute (JCVI) or GenBank database (http://www.ncbi.nlm.nih.gov/sutils/genom_table.cgi) of the National Center for Biotechnology Information (NCBI). This strategy identified two operons in pneumococci containing in each case a gene encoding a putative extracellular thioredoxin-like lipoprotein, here referred to as Etrx1 or Etrx2 (extracellular thioredoxin) protein. In depth bioinformatic analysis revealed that the gene encoding Etrx1 is located in a highly conserved genomic region in which *etrx1* is part of a three-gene operon (TIGR4: *sp_0658*, *sp_0659* (*etrx1*) and *sp_0660*; D39: *spd_0571*, *spd_0572* (*etrx1*), *spd_0573*). The gene upstream of *etrx1* encodes a cytochrome c-type biogenesis protein (CcdA1), while the gene downstream of *etrx1* encodes a methionine sulfoxide reductase (*Sp*MsrAB2) protein that is also exported and anchored to the membrane by an amino-terminal transmembrane region (Fig 1, S2 and S4). The *msr* gene in TIGR4 is not correctly annotated and lacks the 5´end, while in D39 its

annotation is correct (Fig S4). Strikingly, the two component regulatory system 09 (TCS09; TIGR4: *sp_0661 and sp_0662*, D39: *spd_0574* and *spd_0575*) is located downstream of the *etrx1* operon (Fig S1). The gene encoding Etrx2 is also localized in a highly conserved genomic region (Fig S7 and S8). However, *etrx2* has only one partner and forms a bicistronic operon with a cytochrome c-type biogenesis protein (CcdA2) encoding gene (TIGR4: *sp_0999*, *sp_1000* (*etrx2*); D39: *spd_0885*, *spd_0886* (*etrx2*) (Fig 1). The presence of *etrx* genes in pneumococcal laboratory strains and clinically relevant serotypes was indicated by PCR amplification of *etrx1* and *etrx2* sequences (Fig S5).

Bacterial strains, culture conditions, and transformation techniques

E. coli strains and *S. pneumoniae* genotypes and strains used in this study are listed in Table S1. *E. coli and S. pneumoniae strains* were cultured and transformed as described recently (Jensch et al, 2010). Briefly, *E. coli* were cultured on Luria-Bertani (LB) plates or in LB broth, which were supplemented with ampicillin (100 µg/ml) erythromycin (250 µg/ml), kanamycin (50 µg/ml) and/or spectinomycin (100 µg/ml). Transformation of *E. coli* with plasmid DNA was carried out with $CaCl_2$-treated competent cells or by electroporation according to standard procedures. *S. pneumoniae* and isogenic mutants of serotype 2 strain D39 (NCTC7466) were grown on blood agar plates (Oxoid, Germany), cultured in Todd-Hewitt broth (Oxoid, Basingstoke, England) supplemented with 0.5% yeast extract (THY; Roth, Karlsruhe, Germany) or in chemically defined medium (CDM) (Hartel et al, 2012). Cultivation of pneumococci was conducted at 37°C and 5% CO_2, and liquid cultures were grown without agitation to mid-log phase (A_{600} 0.35 to 0.4). Pneumococci were transformed as described previously (Hammerschmidt et al, 2007) using competence-stimulating peptide-1 and cultivated in the presence of the appropriate antibiotics: erythromycin (5 µg/ml), kanamycin (50 or 150 µg/ml) and/or spectinomycin (50 µg/ml).

Primers and molecular techniques

Primers that were used in this study and plasmids used for the mutagenesis and recombinant protein expression are listed in Table S2. Isolation and purification of genomic pneumococcal DNA was performed using the QIAGEN Genomic Tip 100/G (Qiagen, Hilden, Germany) according to the manufacturer´s instructions with slight modifications described earlier (Hammerschmidt et al, 1997). DNA amplifications needed for mutagenesis were carried out by PCR. To amplify pneumococcal DNA by PCR the Taq DNA polymerase (New England Biolabs, Frankfurt, Germany) was used and the reactions were subjected to 30 cycles of denaturation at 94°C, primer annealing for 30 sec, and elongation at 72°C. The annealing temperature depended on the primers and extension time on the length of PCR product. For expression cloning the proofreading Pfu polymerase was used as specified by the manufacturer (Stratagene, LaJolla, U.S.). Oligonucleotides were synthesized by Eurofins MWG Operon (Germany). PCR products were purified with the PCR DNA purification kit (Qiagen, Hilden, Germany) and plasmids were isolated and purified with the Qiaprep Spin Midi or Maxiprep Kit (Qiagen, Hilden, Germany). The integrity of the DNA was confirmed by sequencing (Eurofins MWG Operon).

Construction of pneumococcal mutants

For the generation of the pneumococcal mutants in D39*lux* (Jensch et al, 2010) and D39Δ*cps* the insertion-deletion mutagenesis strategy was used as described (Rennemeier et al, 2007). To generate *ccdA*-, *etrx*-, and *msrAB*-mutants, respectively, the loci of the genes and their upstream and downstream flanking sequences were PCR amplified from *S. pneumoniae* TIGR4 (serotype 4) genomic DNA as template. Primer pairs ccdA1_905/ccdA1_908 and ccdA2_909/ccdA2_912 were used for the *ccdA1* and *ccdA2* gene, respectively (Table S2). Primer pairs etrx1_402/etrx1_405 and etrx2_423/etrx2_426 were used for the *etrx1* and *etrx2*

gene, respectively (Table S2). Primer pairs msrAB1_769/msrAB1_772 and msrAB2_700/msrAB2_701 were used for the *msrAB1* and *msrAB2* gene, respectively (Table S2). The PCR products were directly cloned into pGEM®-T Easy according to the manufacturer´s instructions (Promega, Madison, U.S.) and transformed into the *E. coli* DH5α competent cells. The recombinant plasmids harboring the desired DNA-inserts were separately isolated, purified and used as templates for inverse PCR reaction with the indicated primer pairs with incorporated restriction sites. The deleted *ccdA, etrx, or msrAB* sequences were replaced with the *ermB* or *aad9* gene (Figure 1), which were PCR amplified from plasmid pE89 or pE96 using primer ermB_105/ ermB_106 and aad9_117/ aad9_118, respectively (Table S2). The integrity of the DNA-inserts was confirmed by PCR, restriction analysis, and DNA sequencing (data not shown). These recombinant plasmids were used to transform pneumococci as described previously (Hammerschmidt et al, 1997) and mutants were selected by the addition of erythromycin (5 µg/ml) or spectinomycin (50 µg/ml) or a combination of antibiotics for the double mutants *S. pneumoniae ΔccdA1ΔccdA2, Δetrx1Δetrx2, and ΔmsrAB1ΔmsrAB2*, repectively. Growth of the mutants was not significantly affected compared to D39, with the exception of the *msrAB1*-mutant in CDM (Fig S15). The lack of Etrx or MsrAB production was confirmed by immunoblot analysis using mouse anti-Etrx1, mouse anti-Etrx2, or mouse anti-MsrAB2 antisera, respectively (Fig 1, Fig 2, Fig S11, Fig S12). To verify the stability of the mutants they were cultivated at least two times in liquid culture without antibiotics before identical culture volumes were spread on blood agar plates with or without erythromycin or spectinomycin. As at least more than 90% of the pneumococci grow on blood agar with erythromycin the mutants were considered to be stable (data not shown).

Expression cloning, His$_6$-tagged protein purification, generation of polyclonal anti-Etrx/anti-MsrAB2 antisera and immunoblotting

The *etrx1* (*sp_0659*; nt 63 to nt 567; residues 21 to 181), *etrx2a* (*sp_1000*; nt 60 to nt 558; residues 20 to 185) and *etrx2b* (*sp_1000*; nt 129 to nt 558; residues 43 to 185) gene sequences were amplified by PCR with primer pairs etrx1_447/etrx1_448 and etrx2_486/etrx2_487, respectively, and *S. pneumoniae* TIGR4 chromosomal DNA as template. Primers had incorporated *Nhe*I/*Hin*dIII and *Nhe*I/*Sac*I restriction sites, respectively (Primers are listed in Table S2). The signal sequences of the genes were not amplified and the PCR products were cloned into the similarly digested pTP1 expression vector. This vector is a modified pET-28 vector (Novagen, Darmstadt, Germany), which allows removal of the attached N-terminal His-tag from the recombinant protein by using TEV protease. In the pET-28TEV vector the thrombin cleavage site was replaced by the TEV protease cleavage of the pProEx HTa vector (Life Technologies, Darmstadt, Germany) and also the multiple cloning site was slightly modified. Similar cloning strategies were used for *msrAB2*, *msrA2*, and *msrB2*. The *msrAB2* (*sp_0660*; nt 181 to nt 1110; residues 61 to 370), *msrA2* (nt 181 to nt 654; residues 61 to 218) and *msrB2* (nt 688 to nt 1110; residues 230 to 370) genes were amplified with primer pairs msrAB2_863/msrAB2_692, msrAB2_863/msrA2_1051, and msrB2_1052/msrAB2_692, respectively. The plasmids containing *etrx* or *msr* sequences were transformed into *E. coli* BL21 (DE3) resulting in plasmids p629 (*etrx1*), p651 (*etrx2*), p792 (*msrAB2*), p888 (*msrA2*), p891 (*msrB2*), respectively. Integrity of insert DNA was confirmed by DNA sequencing. For protein production the recombinant *E. coli* BL21 (DE3) harboring the plasmids p629, p651, or p807 were cultured in LB broth, supplemented with kanamycin (50 µg/ml), and grown to an A_{600} of 0.5 to 0.7 at 30°C. Protein expression was then induced with 1 mM IPTG (Isopropyl-β-D-1-thiogalactopyranoside) and cultivation was continued for 4 h.

The His_6-tagged Etrx and Msr proteins were purified by affinity chromatography using His Trap™ HP Ni-NTA columns (1ml; GE Healthcare, Chalfont St Giles, UK) and the ÄKTApurifier liquid chromatography system (GE Healthcare) according to the instructions of the manufacturers. The His_6-tag was removed by TEV protease cleavage overnight on ice followed by a further purification step using the His Trap™ HP column. The purified rEtrx and rMsr proteins (without His_6-tag) were dialyzed against 20 mM Tris-HCl (pH8) and concentrated to 10-25 mg/ml using Vivaspin Ultra concentrators (Sartorius, Göttingen, Germany). Purity of the proteins was analyzed after sodium dodecyl sulfate polyacrylamide gel electrophoresis (SDS-PAGE) by both Coomassie brilliant-blue (CBB) staining and immunoblotting. Detection of rEtrx and rMsrAB2 proteins (or *S. pneumoniae* Etrx and MsrAB proteins) was carried out by using Penta-His™ antibodies (QIAGEN; 1:500 in PBS) and/or mouse anti-Etrx/anti-MsrAB polyclonal antisera (1:1000). Importantly, anti-MsrAB2 antibodies cross-reacted with the intracellular MsrAB1 protein, which is due to the high sequence homology. After SDS-PAGE separation of His_6-tagged Etrx and MsrAB2 proteins, respectively, or bacterial lysates, proteins were transferred to a nitrocellulose membrane by using a semidry blotting system (Bio-Rad, Laboratories, Munich, Germany). The membrane was blocked with 5% skim milk (Roth, Karlsruhe, Germany). As secondary antibodies goat anti-mouse Ig peroxidase conjugate (Dianova; 1:5000) were used and binding activity was detected by enhanced chemiluminescence (Luminol and p-coumaric acid, Roth). Antibodies against rEtrx proteins or rMsrAB2 were raised in 6 to 8 weeks old female CD-1 mice (Charles River Laboratories, Sulzfeld Germany) by immunizing mice intraperitoneally with 100 µl of a 1:1 emulsion containing 50 µg recombinant protein (rEtrx1, rEtrx2, or rMsrAB2) and complete Freund`s adjuvant (Sigma-Aldrich, Taufkirchen, Germany). Mice were boosted with an emulsion of protein and incomplete Freund´s adjuvant at day 14 and 28 and bled after six weeks.

Flow cytometry

S. pneumoniae wild-type and isogenic mutants were cultured in 30 ml THY to A_{600} = 035 – 0.4 and after sedimentation the bacteria were washed with RPMI1640/1% FBS (fetal bovine serum; PAA Laboratories, Colbe, Germany) and finally resuspended in 1 ml of RPMI1640/1% FBS. To detect Etrx proteins on the surface of pneumococci 1 x 10^8 bacteria were incubated with mouse anti-Etrx polyclonal antisera (1:25 dilution in PBS) or antisera of mice injected with PBS and incubated for 30 min at 4°C. Samples were then washed twice with PBS/0.5% FCS and stained with secondary goat anti-mouse IgG coupled Alexa-Fluor-488 (Invitrogen). After 30 min incubation at 4°C bacteria were washed twice with PBS/0.5% and then fixed with 2% formaldehyde. Flow cytometry was conducted with the FACSCalibur™ (BD Biosciences, Heidelberg, Germany) and the CellQuestPro Software 6.0. (BD Biosciences) was used for data acquisition while analysis of the data was performed with the software WinMDI 2.9. The bacteria were detected and gated as described previously (Jensch et al, 2010) and the forward scatter (FL1-H) in the histograms (Fig 2A) show the increase in fluorescence intensity.

Subcellular fractionation of pneumococcal proteins

Subcellular fractionation of S. *pneumoniae* proteins was performed as described earlier (Andisi et al, 2012; Bergmann et al, 2001). Briefly, pneumococci were grown in THY media to an A_{600} of 0.35 and harvested by centrifugation. The bacterial sediment was resuspended in lysis buffer (100 mM Tris-HCl, pH 8.0, 20% sucrose, 20 mM $MgCl_2$) containing lysozyme (5 mg/ml), mutanolysin (200 U/ml), and 1 x Complete protease inhibitor cocktail (Roche Diagnostics, Mannheim, Germany) and incubated for 30 min at 37°C. After centrifugation at 3000 x g and 4°C for 10 min the protoplasts were resuspended in one volume of sucrose buffer (20% sucrose, 10 mM Tris-HCl, pH 8.0) and disrupted by the addition of 19 volumes

of 100 mM Tris-HCl with 1 mM EDTA and 1x Complete protease inhibitor cocktail. Remaining protoplasts were removed by centrifugation at 4000 x g and 4°C for 10 min. Membranes and the cytoplasmic protein fraction were separated by ultracentrifugation (100000 x g and 4°C for 30 min) and subcellular fractions were analyzed by SDS-PAGE and immunoblot.

Determination of methionine sulfoxide reductase activity and the redox potential of proteins

NADPH-dependent methionine sulfoxide reductase activity was measured in a mix containing 50 mM Tris/HCl pH 7.4, 200 µM NADPH, 100 nM human thioredoxin reductase (Johansson et al, 2004), 26 mM DL-methionine sulfoxide (Sigma-Aldrich), and 5-75 µM of the thioredoxin proteins Etrx1 and Etrx2 (as indicated) at 25°C. The assay was started by addition of up to 1.5 mM of the methionine sulfoxide reductase subunits to the assay mixture (as indicated) in a total volume of 200 µl in 96 well plates. The decrease in absorbance at 340 nm due to the oxidation of NADPH was recorded in a micro plate reader (Tecan, Crailsheim, Germany) and used for the calculation of the specific activity in nmol substrate converted per minute per mg of enzyme (n =3).

The redox state and potential of the proteins were determined by specific alkylation of only reduced thiols (Zander et al, 1998) using 4-acetamido-4'maleimidylstilbene 2,2'disolfonic acid (AMS, Sigma-Aldrich) that adds 536 Da mass to the proteins per thiol alkylated. In brief, proteins (5 µM) were incubated in glutathione redox buffers adjusted to the potentials desired by mixing GSH and GSSG at a total glutathione concentration of 6 mM at different ratios, calculated with the help of the Nernst equation assuming a $E^{0'}$ of -260 mV for the GSH/GSSH redox couple, at 25 °C for three hours in an anaerobic nitrogen environment. Next, all remaining free thiols were alkylated with an access of AMS (5 mM) for 30 min. Mass shifts

were analyzed by SDS PAGE using 18 % Criterion TGX Stain-Free Precast gels visualized with a Criterion Stain-Free imager (Biorad).

Non-reducing/Reducing Diagonal 2D-SDS-PAGE and Western blot analysis. *S. pneumoniae* wild-type bacteria (WT) and isogenic mutants were grown in THY medium to an OD_{600} of 0.35 and harvested under non-stress control conditions in TE-buffer (10 mM Tris-HCl, pH 8.0, 1 mM EDTA) with 20 mM N-ethylmaleimide (NEM) to alkylate all reduced thiols. Pneumococci were disrupted by ultrasonication and the protein extracts obtained after repeated centrifugation. Protein extracts were separated using the non-reducing/reducing diagonal 2D-SDS-PAGE analysis as described (Pother et al, 2009) and subjected to MsrAB-specific immunoblot-blot analysis. This diagonal assay distinguishes intramolecular and intermolecular disulfides in proteins (Leichert & Jakob, 2006). In brief, proteins were first separated using non-reducing SDS-PAGE (1D) without DTT and the lanes were cut. The gel lanes were incubated in SDS sample buffer containing 50 mM DTT to reduce all protein disulfides and the newly formed thiols were subsequently alkylated in SDS sample buffer with iodoacetamide to alkylate all newly formed reduced thiols. Bands were positioned horizontally on an SDS-PAGE and separated using reducing SDS-PAGE (2D). The reducing diagonal SDS-gel (2D) was subjected to *Sp*MrAB-and pneumococcus-specific immunoblot analysis. The non-reducing one-dimensional gel (1D) was subjected to *Sp*MrAB immunoblot analysis. All proteins that migrate along the diagonal represent reduced thiol-containing proteins. Those proteins that form intermolecular disulfides run in the second SDS-PAGE below the diagonal. Proteins with intramolecular disulfides including MsrAB1 and MsrAB2 are located above to the diagonal. Anti-pneumococcus antiserum (Jensch et al. 2010) was used in combination with the MsrAB-specific antiserum to visualize the diagonal.

Etrx1 Crystallization, Data Collection and Processing

Prior to crystallization experiments, monomeric state of the sample was determined by ultra-analytical centrifugation (data not shown). Etrx1 crystals were obtained by the hanging-drop vapour diffusion method at 18ºC mixing 21 mg/ml protein solution with 30% (v/v) PEG 4000; 0. 1M Tris pH 8.5; 0.2 M $MgCl_2$ in 1:1 volume ratio drops. Prior to X-ray data collection, cryo-protected crystals were captured in nylon loops and flash-cooled under a nitrogen flow at 100 K. Cryoprotectant solution used contained 100% Paratone-N. X-ray diffraction data sets were collected at beamline ID14-4 on the European Synchrotron Radiation Facility (ESRF, Grenoble, France) using an ADSC Q210 CCD detector. The Etrx1 crystals diffracted up to 1.3Å resolution and belong to the $P4_32_12$ tetragonal space group (a= 62.85 Å, b= 62.85 Å, c=86.60 Å, $\alpha=\beta=\gamma$= 90º). One single monomer was found in the asymmetric unit yielding a Matthews' coefficient (Matthews, 1968) of 2.67 $Å^3Da^{-1}$ and a solvent content of 53.9 %. Data sets were processed with IMOSFLM (Battye et al, 2011) and scaled up to 1.48 Å with SCALA (Cowtan et al, 2011).

Etrx1 Structure Determination and Refinement

The structure was solved by molecular replacement with Balves server (http://www.ysbl.york.ac.uk/YSBLPrograms/index.jsp) using N-term-PilB homolog (PDB code 2H30) as a search model (Brot et al, 2006). Refinement and map calculations were performed with simulated annealing in Phenix (Adams et al, 2010). Then, successive cycles of manual rebuilding were performed with Coot (Emsley & Cowtan, 2004). Further refinement was performed in Phenix (Adams et al, 2010). The refinement converged to the final values of R = 0.18 and R_{free} = 0.20 (Table 1). Analysis of the model with MolProbity showed a good stereochemistry (Chen et al, 2010). The excellent quality of electron density maps allowed the modeling of Etrx1 from Ala53 to Leu187. The final model presents 135

amino acids with and the first 22 amino acids from the N-terminal tail were disordered and were not visible in the structure. All graphics representations were prepared with PyMOL. During the preparation of this manuscript the Etrx1 related thioredoxin from *S. pneumoniae* strain Canada MDR_19A (PDB: 4EVM) has been deposited on PDB database by the Center for Structural Genomics of Infectious Diseases structural genomics consortium.

Etrx2 Crystallization, Data Collection and Processing

Two Etrx2 crystalline forms were obtained. Etrx2 in complex with 2-Hydroxyethyl Disulfide (HED) crystals were obtained by the hanging-drop vappour-diffusion method at 18ºC by mixing 10 mg/ml protein solution previously incubated with β-mercaptoethanol (14 mM end concentration) for 2 h at 4ºC with a solution of 30% PEG 1500 in 1:1 volume ratio drops. Etrx2 crystals in complex with Cyclofos-3^{TM} detergent were obtained by the micro-batch crystallization method at 18ºC mixing 10mg/ml protein solution with 3.4 M sodium malonate pH 6.0 into 1:1 volume ratio drops covered with al's oils (Hampton Research, USA) to prevent excessive evaporation. Droplets were supplemented with 86 mM (end concentration) Cyclofos-3^{TM} (3-Cyclohexyl-1-propylphosphocholine) detergent. Preceding X-ray data collection, crystals captured in nylon loops were soaked in a 100% Paratone-N and and flash-cooled under a nitrogen flow at 100 K. X-ray diffraction Etrx2:HED datasets were obtained at SLS beam-line PXIII with a Pilatus 2M detector while Etrx2:Cyclofos-3^{TM} data sets were collected at beam-line ID14-1 on the ESRF using an ADSC Q210 CCD detector. The Etrx2:HED crystals diffracted up to 1.2 Å resolution and belongs to the P1 triclinic space group (a= 31.25 Å, b= 35.88 Å, c= 58.21 Å, α=102.02, β=99.28, γ=101.57). Two monomers were found in the asymmetric unit yielding a Matthews coefficient (Matthews, 1968) of 1.93 $Å^3$/Da and a solvent content of 36.35%. The Etrx2:Cyclofos-3^{TM} crystal diffracted up to 1.7 Å resolution, and belonged to the $P2_12_12_1$ orthorhombic space group (a= 61.40 Å, b= 116.31 Å,

c= 116.42 Å). Four monomers were found in the asymmetric unit yielding a Matthew's coefficient (Matthews, 1968) of 2.80 Å^3/Da and a solvent content of 56.03%. Data sets were processed with IMOSFLM (Battye et al, 2011) and scaled up with SCALA (Cowtan et al, 2011).

Etrx2 Structure Determination and Refinement

The structure was solved by molecular replacement with Balves server (http://www.ysbl.york.ac.uk/YSBLPrograms/index.jsp) using the extracytoplasmic thioredoxin ResA (PDB code 2H1B) as a search model (Lewin et al, 2006). Refinement and map calculation were performed with simulated annealing in Phenix (Adams et al, 2010). Then, successive cycles of manual rebuilding were performed with Coot (Emsley & Cowtan, 2004). Further refinement was performed with Phenix (Adams et al, 2010). The refinement converged to the final values of R = 0.16 and R_{free} = 0.19 for the Etrx2:Cyclofos-3^{TM} form and R = 0.15 and R_{free} = 0.18 for the Etrx2:HED form (Table 1). Further analysis of the model with MolProbity showed a good stereochemistry (Chen et al, 2010). All graphics representations were prepared with PyMOL.

***Sp*MsrAB2 homology model**

The 3D model for the catalytic domains of *Sp*MsrAB2 (residues 60 to 312 in SP_0660 of TIGR4 or SPD_0573 in D39) was obtained by comparative homology search against PDB structural database by automatic ESyPred3D (via modeller) server (Lambert et al, 2002). The crystal structure of cytoplasmic pneumococcal *Sp*MsrAB1 (PDB code 3OEM) was selected and used as template. The model structure was verified by PROSA analysis and GROMOS96 was used for further optimization of the model by energy minimization. TMHMM 2.0 software (http://www.cbs.dtu.dk/services/TMHMM/) predicted the presence of a

transmembrane helix between residues 7 to 28, that was confirmed by SignalP 4.0 (Petersen et al, 2011). An additional short helix (residues 35 to 39) was predicted with PSIPRED (McGuffin et al, 2000). Both structural elements were ideally modelled with Coot (Emsley & Cowtan, 2004) and added to the MrsAB homology model.

Phagocytosis experiments

To determine the rate of phagocytosed wild-type and mutant pneumococci and their intracellular survival in macrophages, phagocytosis experiment with J774A.1 murine macrophages (DSMZ, Braunschweig, Germany) were carried out as described (Hartel et al, 2011; Jensch et al, 2010). Briefly, confluent monolayers of J774 cells in 96-well cell culture plates and cultured in RPMI1640/10% FBS (PAA Laboratories) were incubated for 30 min with pneumococci. Extracellular bacteria and non-adherent pneumococci were then killed by the antibiotic protection assay. The infected cells were washed three times with the infection medium and then replaced and incubated for 1 h with RPMI1640 medium containing gentamicin (100 μg/ml) and penicillin G (100 units/ml) to kill non-internalized bacteria. Intracellular pneumococci were recovered by a saponin-mediated lysis (1% w/v) of macrophages (Jensch et al, 2010) and the number of recovered and viable pneumococci (survivors) was determined by quantitative plating of the released intracellular pneumococci on sheep blood agar plates. Experiments were conducted at least three times with four replicate wells. Fluorescence microscopy was performed to visualize attached or ingested pneumococci. Macrophages (10^5) were seeded on glass cover slips (12 mm ∅) in wells of 24 cell culture plate and infected the following day with pneumococci as described above. Post infection unbound bacteria were removed and the infected host cells were fixed on the glass cover slips with 3.7% paraformaldehyde) and double immunofluorescence microscopy was carried out as described (Jensch et al, 2010). Image acquisition was performed with a confocal

laser scanning microscope (Zeiss LSM510 META) and the LSM software. Each bar in the images represents 20 µm. All experiments were performed at least three times with two or more replicate wells tested for each experimental setup.

REFERENCES

Adams PD, Afonine PV, Bunkoczi G, Chen VB, Davis IW, Echols N, Headd JJ, Hung LW, Kapral GJ, Grosse-Kunstleve RW et al (2010) PHENIX: a comprehensive Python-based system for macromolecular structure solution. Acta Crystallogr D Biol Crystallogr 66**:** 213-221

Andisi VF, Hinojosa CA, de Jong A, Kuipers OP, Orihuela CJ, Bijlsma JJ (2012) Pneumococcal gene complex involved in resistance to extracellular oxidative stress. Infect Immun 80**:** 1037-1049

Battye TG, Kontogiannis L, Johnson O, Powell HR, Leslie AG (2011) iMOSFLM: a new graphical interface for diffraction-image processing with MOSFLM. Acta Crystallogr D Biol Crystallogr 67**:** 271-281

Bergmann S, Rohde M, Chhatwal GS, Hammerschmidt S (2001) alpha-Enolase of Streptococcus pneumoniae is a plasmin(ogen)-binding protein displayed on the bacterial cell surface. MolMicrobiol 40**:** 1273-1287

Bergmann S, Wild D, Diekmann O, Frank R, Bracht D, Chhatwal GS, Hammerschmidt S (2003) Identification of a novel plasmin(ogen)-binding motif in surface displayed alpha-enolase of Streptococcus pneumoniae. MolMicrobiol 49**:** 411-423

Brot N, Collet J-F, Johnson LC, Jönsson TJ, Weissbach H, Lowther WT (2006) The thioredoxin domain of Neisseria gonorrhoeae PilB can use electrons from DsbD to reduce downstream methionine sulfoxide reductases. The Journal of biological chemistry 281**:** 32668-32675

Chen VB, Arendall WB, Headd JJ, Keedy DA, Immormino RM, Kapral GJ, Murray LW, Richardson JS, Richardson DC (2010) MolProbity: all-atom structure validation for macromolecular crystallography. Acta Crystallogr D Biol Crystallogr 66**:** 12-21

Cowtan K, Emsley P, Wilson KS (2011) From crystal to structure with CCP4. Acta Crystallogr D Biol Crystallogr 67**:** 233-234

Emsley P, Cowtan K (2004) Coot: model-building tools for molecular graphics. Acta Crystallogr D Biol Crystallogr 60**:** 2126-2132

Hammerschmidt S, Agarwal V, Kunert A, Haelbich S, Skerka C, Zipfel PF (2007) The host immune regulator factor H interacts via two contact sites with the PspC protein of *Streptococcus pneumoniae* and mediates adhesion to host epithelial cells. J Immunol 178**:** 5848-5858

Hammerschmidt S, Talay SR, Brandtzaeg P, Chhatwal GS (1997) SpsA, a novel pneumococcal surface protein with specific binding to secretory immunoglobulin A and secretory component. Mol Microbiol 25**:** 1113-1124

Hammerschmidt S, Tillig MP, Wolff S, Vaerman JP, Chhatwal GS (2000) Species-specific binding of human secretory component to SpsA protein of *Streptococcus pneumoniae* via a hexapeptide motif. Mol Microbiol 36**:** 726-736

Hartel T, Eylert E, Schulz C, Petruschka L, Gierok P, Grubmuller S, Lalk M, Eisenreich W, Hammerschmidt S (2012) Characterization of central carbon metabolism of *Streptococcus pneumoniae* by isotopologue profiling. J Biol Chem 287**:** 4260-4274

Hartel T, Klein M, Koedel U, Rohde M, Petruschka L, Hammerschmidt S (2011) Impact of glutamine transporters on pneumococcal fitness under infection-related conditions. Infect Immun 79**:** 44-58

Holmes AR, McNab R, Millsap KW, Rohde M, Hammerschmidt S, Mawdsley JL, Jenkinson HF (2001) The pavA gene of Streptococcus pneumoniae encodes a fibronectin-binding protein that is essential for virulence. MolMicrobiol 41**:** 1395-1408

Jensch I, Gamez G, Rothe M, Ebert S, Fulde M, Somplatzki D, Bergmann S, Petruschka L, Rohde M, Nau R et al (2010) PavB is a surface-exposed adhesin of *Streptococcus pneumoniae* contributing to nasopharyngeal colonization and airways infections. Mol Microbiol 77**:** 22-43

Johansson C, Lillig CH, Holmgren A (2004) Human mitochondrial glutaredoxin reduces S-glutathionylated proteins with high affinity accepting electrons from either glutathione or thioredoxin reductase. J Biol Chem 279**:** 7537-7543

Johnston JW, Myers LE, Ochs MM, Benjamin WH, Jr., Briles DE, Hollingshead SK (2004) Lipoprotein PsaA in virulence of Streptococcus pneumoniae: surface accessibility and role in protection from superoxide. Infect Immun 72**:** 5858-5867

Lambert C, Leonard N, De Bolle X, Depiereux E (2002) ESyPred3D: Prediction of proteins 3D structures. Bioinformatics 18**:** 1250-1256

Leichert LI, Jakob U (2006) Global methods to monitor the thiol-disulfide state of proteins in vivo. Antioxid Redox Signal 8**:** 763-772

Lewin A, Crow A, Oubrie A, Le Brun NE (2006) Molecular basis for specificity of the extracytoplasmic thioredoxin ResA. The Journal of biological chemistry 281**:** 35467-35477

Matthews BW (1968) Solvent content of protein crystals. J Mol Biol 33**:** 491-497

McGuffin LJ, Bryson K, Jones DT (2000) The PSIPRED protein structure prediction server. Bioinformatics 16**:** 404-405

Petersen TN, Brunak S, von Heijne G, Nielsen H (2011) SignalP 4.0: discriminating signal peptides from transmembrane regions. Nat Methods 8**:** 785-786

Pother DC, Liebeke M, Hochgrafe F, Antelmann H, Becher D, Lalk M, Lindequist U, Borovok I, Cohen G, Aharonowitz Y et al (2009) Diamide triggers mainly S Thiolations in the cytoplasmic proteomes of Bacillus subtilis and Staphylococcus aureus. J Bacteriol 191**:** 7520-7530

Pracht D, Elm C, Gerber J, Bergmann S, Rohde M, Seiler M, Kim KS, Jenkinson HF, Nau R, Hammerschmidt S (2005) PavA of *Streptococcus pneumoniae* modulates adherence, invasion, and meningeal inflammation. Infect Immun 73**:** 2680-2689

Rennemeier C, Hammerschmidt S, Niemann S, Inamura S, Zahringer U, Kehrel BE (2007) Thrombospondin-1 promotes cellular adherence of gram-positive pathogens via recognition of peptidoglycan. FASEB J 21**:** 3118-3132

Tettelin H, Nelson KE, Paulsen IT, Eisen JA, Read TD, Peterson S, Heidelberg J, DeBoy RT, Haft DH, Dodson RJ et al (2001) Complete genome sequence of a virulent isolate of *Streptococcus pneumoniae*. Science 293**:** 498-506

Tomasz A, Hotchkiss RD (1964) Regulation of the Transformability of Pheumococcal Cultures by Macromolecular Cell Products. Proc Natl Acad Sci U S A 51**:** 480-487

Voss S, Hallstroem T, Saleh M, Burchhardt G, Pribyl T, Singh B, Riesbeck K, Zipfel PF, Hammerschmidt S (2013) The choline-binding protein PspC of Streptococcus pneumoniae interacts with the C-terminal heparin-binding domain of vitronectin. J Biol Chem

Zander T, Phadke ND, Bardwell JC (1998) Disulfide bond catalysts in Escherichia coli. Methods Enzymol 290**:** 59-74

Figure S1

```
Etrx1    MKKWQTCVLGAGSLLCLTACSGKSVTSEHQTKDEMKTEQTASKTSAAKGKEVADFELMGV 60
Etrx2    MKKVMFAGLSLLSLVVLMAC-GEEETKKTQAAQQPKQQTTVQQISVG--KDVPDFTLQSM 57
         ***   . *.  **: * ** *:. *.: *: :: * : *..: *..  *:*.** * .:

                              CXXC motif
Etrx1    DGKTYRLSDYKGKKVYLKFWASWCSICLASLPDTDEIAKEAGDDYVVLTVVSPGHKGEQS 120
Etrx2    DGKEVKLSDFKGKKVYLKFWASWCGPCKKSMPELMELAAKPDRDFEILTVIAPGIQGEKT 117
         ***  :***:**************. *  *:*:  *:* :.. *: :***::** :**::

Etrx1    EADFKNWYKGLDYKNLPVLVDPSGKLLETYGVRSYPTQAFIDKEGKLVKTHPGFMEKDAI 180
Etrx2    VEQFPQWFQEQGYKDIPVLYDTKATTFQAYQIRSIPTEYLIDSQGKIGKIQFGAISNADA 177
           :* :*::  .**::*** *.... :::* :** **: :**.:**: * : * :.:

Etrx1    LQTLKELS 188
Etrx2    EAAFKEMN 185
           ::**:.
```

Figure S1. Comparison of Etrx1 (SPD_0572) and Etrx2 (SPD_0886) protein sequences of *S. pneumoniae* D39, showing an identity of 39.4%.

Figure S2

```
TIGR4_spn_SP_0658                 MGHIFFFLSVFLAGILSFFSPCILPLLPVYTGVLLDDKDGAQASSGKFSISVTSLLRTLAFIAGISFIFILLGYGAGFLGDLLYASWFQYLTGAIIIILLGLHQMEILHFKGLYKEKRLQL
D39_spd_SPD_0571                  MGHIFFFLSVFLAGILSFFSPCILPLLPVYTGVLLDDKDGAQASSGKFSISVTSLLRTLAFIAGISFIFILLGYGAGFLGDLLYASWFQYLTGAIIIILLGLHQMEILHFKGLYKEKRLQL
R6_spr_spr0575                    MGHIFFFLSVFLAGILSFFSPCILPLLPVYTGVLLDDKDGAQASSGKFSISVTSLLRTLAFIAGISFIFILLGYGAGFLGDLLYASWFQYLTGAIIIILLGLHQMEILHFKGLYKEKRLQL
G54_spx_SPG_0599                  MGHIFFFLSVFLAGILSFFSPCILPLLPVYTGVLLDDKDGAQASSGKFSISVTSLLRTLAFIAGISFIFILLGYGAGFLGDLLYASWFQYLTGAIIIILLGLHQMEILHFKGLYKEKRLQL
CGSP14_spw_SPCG_0614              MGHIFFFLSVFLAGILSFFSPCILPLLPVYTGVLLDDKDGAQASSGKFSISVTSLLRTLAFIAGISFIFILLGYGAGFLGDLLYASWFQYLTGAIIIILLGLHQMEILHFKGLYKEKRLQL
ATCC700669_sne_SPN23F_05930       MGHIFFFLSVFLAGILSFFSPCILPLLPVYTGVLLDDKDGAQASSGKFSISVTSLLRTLAFIAGISFIFILLGYGAGFLGDLLYASWFQYLTGAIIIILLGLHQMEILHFKGLYKEKRLQL
Hungary19A_6_spv_SPH_0753         MGHIFFFLSVFLAGILSFFSPCILPLLPVYTGVLLDDKDGAQASSGKFSISVTSLLRTLAFIAGISFIFILLGYGAGFLGDLLYASWFQYLTGAIIIILLGLHQMEILHFKGLYKEKRLQL
JJA_sjj_SPJ_0607                  MGHIFFFLSVFLAGILSFFSPCILPLLPVYTGVLLDDKDGAQASSGKFSISVTSLLRTLAFIAGISFIFILLGYGAGFLGDLLYASWFQYLTGAIIIILLGLHQMEILHFKGLYKEKRLQL
670_6B_snb_SP670_0716             MGHIFFFLSVFLAGILSFFSPCILPLLPVYTGVLLDDKDGAQASSGKFSISVTSLLRTLAFIAGISFIFILLGYGAGFLGDLLYASWFQYLTGAIIIILLGLHQMEILHFKELYKEKRLQL
P1031_spp_SPP_0678                MGHIFFFLSVFLAGILSFFSPCILPLLPVYTGVLLDDKDGAQASSGKFSISVTSLLRTLAFIAGISFIFILLGYGAGFLGDLLYASWFQYLTGAIIIILLGLHQMEILHFKGLYKEKRLQL
TCH8431/19A_snc_HMPREF0837_10950  MGHIFFFLSVFLAGILSFFSPCILPLLPVYTGVLLDDKDGAQASSGKFSISVTSLLRTLAFIAGISFIFILLGYGAGFLGDLLYASWFQYLTGAIIIILLGLHQMEILHFKGLYKEKRLQL
Taiwan19F_snt_SPT_0682            MGHIFFFLSVFLAGILSFFSPCILPLLPVYTGVLLDDKDGAQASSGKFSISVTSLLRTLAFIAGISFIFILLGYGAGFLGDLLYASWFQYLTGAIIIILLGLHQMEILHFKGLYKEKRLQL

TIGR4_spn_SP_0658                 QGQGQNGKGYSQAFLLGLTFSFAWTPCVGPVLGSVLALAASGGSGAWQGAGLMLVYTLGLALPFLLLALTSSYVLKHFRKLHPYLGILKKVGGFLIIVMGFLVLFGNASILSQLFE
D39_spd_SPD_0571                  QGQGQNGKGYSQAFLLGLTFSFAWTPCVGPVLGSVLALAASGGSGAWQGAGLMLVYTLGLALPFLLLALTSSYVLKHFRKLHPYLGILKKVGGFLIIVMGFLVLFGNASILSQLFE
R6_spr_spr0575                    QGQGQNGKGYSQAFLLGLTFSFAWTPCVGPVLGSVLALAASGGSGAWQGAGLMLVYTLGLALPFLLLALTSSYVLKHFRKLHPYLGILKKVGGFLIIVMGFLVLFGNASILSQLFE
G54_spx_SPG_0599                  QGQGQNGKGYSQAFLLGLTFSFAWTPCVGPVLGSVLALAASGGSGAWQGAGLMLVYTLGLALPFLLLALTSSYVLKHFRKLHPYLGILKKVGGFLIIVMGLLVLFGNASILSQLFE
CGSP14_spw_SPCG_0614              QGQGQNGKGYSQAFLLGLTFSFAWTPCVGPVLGSVLALAASGGSGAWQGAGLMLVYTLGLALPFLLLALTSSYVLKHFRKLHPYLGILKKVGGFLIIVMGLLVLFGNASILSQLFE
ATCC700669_sne_SPN23F_05930       QGQGQNGKGYSQAFLLGLTFSFAWTPCVGPVLGSVLALAASGGSGAWQGAGLMLVYTLGLALPFLLLALTSSYVLKHFRKLHPYLGILKKVGGFLIIVMGLLVLFGNASILSQLFE
Hungary19A_6_spv_SPH_0753         QGQGQNGKGYSQAFLLGLTFSFAWTPCVGPVLGSVLALAASGGSGAWQGAGLMLVYTLGLALPFLLLALTSSYVLKHFRKLHPYLGILKKVGGFLIIVMGLLVLFGNASILSQLFE
JJA_sjj_SPJ_0607                  QGQGQNGKGYSQAFLLGLTFSFAWTPCVGPVLGSVLALAASGGSGAWQGAGLMLVYTLGLALPFLLLALTSSYVLKHFRKLHPYLGILKKVGGFLIIVMGLLVLFGNASILSQLFE
670_6B_snb_SP670_0716             QGQGQNGKGYSQAFLLGLTFSFAWTPCVGPVLGSVLALAASGGSGAWQGAGLMLVYTLGLALPFLLLALTSSYVLKHFRKLHPYLGILKKVGGFLIIVMGLLVLFGNASILSQLFE
P1031_spp_SPP_0678                QGQGQNGKGYSQAFLLGLTFSFAWTPCVGPVLGSVLALAASGGSGAWQGAGLMLVYTLGLALPFLLLALTSSYVLKHFRKLHPYLGILKKVGGFLIIVMGLLVLFGNASILSQLFE
TCH8431/19A_snc_HMPREF0837_10950  QGQGQNGKGYSQAFLLGLTFSFAWTPCVGPVLGSVLALAASGGSGAWQGAGLMLVYTLGLALPFLLLALTSSYVLKHFRKLHPYLGILKKVGGFLIIVMGLLVLFGNASILSQLFE
Taiwan19F_snt_SPT_0682            QGQGQNGKGYSQAFLLGLTFSFAWTPCVGPVLGSVLALAASGGSGAWQGAGLMLVYTLGLALPFLLLALTSSYVLKHFRKLHPYLGILKKVGGFLIIVMGLLVLFGNASILSQLFE
```

Figure S2. Comparison of CcdA1 protein sequences of *S. pneumoniae* as deposited in databases for 12 pneumococcal strains.

Figure S3

```
TIGR4_spn_SP_0659                 MKKWQTCVLGAGSLLCLTACSGKSVTSEHQTKDEMKTEQTASKTSAAKGKEVADFELMGVDGKTYRLSDYKGKKVYLKFWASWCSICLASLPDTDEIAKEAGDDYVVLTVVSPGHKGEQS
D39_spd_SPD_0572                  MKKWQTCVLGAGSLLCLTACSGKSVTSEHQTKDEMKTEQTASKTSAAKGKEVADFELMGVDGKTYRLSDYKGKKVYLKFWASWCSICLASLPDTDEIAKEAGDDYVVLTVVSPGHKGEQS
R6_spr_spr0576                    MKKWQTCVLGAGSLLCLTACSGKSVTSEHQTKDEMKTEQTASKTSAAKGKEVADFELMGVDGKTYRLSDYKGKKVYLKFWASWCSICLASLPDTDEIAKEAGDDYVVLTVVSPGHKGEQS
G54_spx_SPG_0600                  MKKWQTCVLGAGSLLCLTACSGKSVTSEHQTKDEMKTEQTASKTSAAKGKEVADFELMGVDGKTYRLSDYKGKKVYLKFWASWCSICLASLPDTDEIAKEAGDDYVVLTVVSPGHKGEQS
CGSP14_spw_SPCG_0615              MKKWQTCVLGAGSLLCLTACSGKSVTSEHQTKDEMKTEQTASKTSAAKGKEVADFELMGVDGKTYRLSDYKGKKVYLKFWASWCSICLASLPDTDEIAKEAGDDYVVLTVVSPGHKGEQS
ATCC700669_sne_SPN23F_05940       MKKWQTCVLGAGSLLCLTACSGKSVTSEHQTKDEMKTEQTASKTSAAKGKEVADFELMGVDGKTYRLSDYKGKKVYLKFWASWCSICLASLPDTDEIAKEAGDDYVVLTVVSPGHKGEQS
Hungary19A_6_spv_SPH_0754         MKKWQTCVLGAGSLLCLTACSGKSVTSEHQTKDEMKTEQTASKTSAAKGKEVADFELMGVDGKTYRLSDYKGKKVYLKFWASWCSICLASLPDTDEIAKEAGDDYVVLTVVSPGHKGEQS
JJA_sjj_SPJ_0608                  MKKWQTCVLGAGSLLCLTACSGKSVTSEHQTKDEMKTEQTASKTSAAKGKEVADFELMGVDGKTYRLSDYKGKKVYLKFWASWCSICLASLPDTDEIAKEAGDDYVVLTVVSPGHKGEQS
670_6B_snb_SP670_0717             MKKWQTCVLGAGSLLCLTACSGKSVTSEHQTKDEMKTEQTASKTSAAKGKEVADFELMGVDGKTYRLSDYKGKKVYLKFWASWCSICLASLPDTDEIAKEAGDDYVVLTVVSPGHKGEQS
P1031_spp_SPP_0679                MKKWQTCVLGAGSLLWLTACSGKSVTSEHQTKDEMKTEQTASKTSAAKGKEVADFELMGVDGKTYRLSDYKGKKVYLKFWASWCSICLASLPDTDEIAKEAGDDYVVLTVVSPGHKGEQS
TCH8431/19A_snc_HMPREF0837_10951  MKKWQTCVLGAGSLLCLTACSGKSVTSEHQTKDEMKTEQTASKTSAAKGKEVADFELMGVDGKTYRLSDYKGKKVYLKFWASWCSICLASLPDTDEIAKEAGDDYVVLTVVSPGHKGEQS
Taiwan19F_snt_SPT_0683            MKKWQTCVLGAGSLLCLTACSGKSVTSEHQTKDEMKTEQTASKTSAAKGKEVADFELMGVDGKTYRLSDYKGKKVYLKFWASWCSICLASLPDTDEIAKEAGDDYVVLTVVSPGHKGEQS


TIGR4_spn_SP_0659                 EADFKNWYKGLDYKNLPVLVDPSGKLLETYGVRSYPTQAFIDKEGKLVKTHPGFMEKDAILQTLKELA
D39_spd_SPD_0572                  EADFKNWYKGLDYKNLPVLVDPSGKLLETYGVRSYPTQAFIDKEGKLVKTHPGFMEKDAILQTLKELS
R6_spr_spr0576                    EADFKNWYKGLDYKNLPVLVDPSGKLLETYGVRSYPTQAFIDKEGKLVKTHPGFMEKDAILQTLKELS
G54_spx_SPG_0600                  EADFKNWYKGLDYKNLPVLVDPSGKLLETYGVRSYPTQAFIDKEGKLVKTHPGFMEKDAILQTLKELA
CGSP14_spw_SPCG_0615              EADFKNWYKGLDYKNLPVLVDPSGKLLETYGVRSYPTQAFIDKEGKLVKTHPGFMEKDAILQTLKELA
ATCC700669_sne_SPN23F_05940       EADFKNWYKGLDYKNLPVLVDPSGKLLETYGVRSYPTQAFIDKEGKLVKTHPGFMEKDAILQTLKELA
Hungary19A_6_spv_SPH_0754         EADFKNWYKGLDYKNLPVLVDPSGKLLETYGVRSYPTQAFIDKEGKLVKTHPGFMEKDAILQTLKELA
JJA_sjj_SPJ_0608                  EADFKNWYKGLDYKNLPVLVDPSGKLLETYGVRSYPTQAFIDKEGKLVKTHPGFMEKDAILQTLKELA
670_6B_snb_SP670_0717             EADFKNWYKGLDYKNLPVLVDPSGKLLETYGVRSYPTQAFIDKEGKLVKTHPGFMEKDAILQTLKELA
P1031_spp_SPP_0679                EADFKNWYKGLDYKNLPVLVDPSGKLLETYGVRSYPTQAFIDKEGKLVKTHPGFMEKDAILQTLKELA
TCH8431/19A_snc_HMPREF0837_10951  EADFKNWYKGLDYKNLPVLVDPSGKLLETYGVRSYPTQAFIDKEGKLVKTHPGFMEKDAILQTLKELA
Taiwan19F_snt_SPT_0683            EADFKNWYKGLDYKNLPVLVDPSGKLLETYGVRSYPTQAFIDKEGKLVKTHPGFMEKDAILQTLKELA
```

Figure S3. Comparison of Etrx1 protein sequences of *S. pneumoniae* as deposited in databases for 12 pneumococcal strains.

Figure S4

```
TIGR4_spn_SP_0660                 MNDKLKIFLLLGVFFLAITGFYVLLIRNAGQTDASQIEKAAVSQGGKAVKKTEISKDADMHEIYLAGGCFWGVEEYFSRVPGVTDAVSGYANGRGETTKYELINQTGHAETVHVTYDAKQ
D39_spd_SPD_0573                  MNDKLKIFLLLGVFFLAITGFYVLLIRNAGQTDASQIEKAAVSQGGKAVKKTEISKDADMHEIYLAGGCFWGVEEYFSRVPGVTDAVSGYANGRGETTKYELINQTGHAETVHVTYDAKQ
R6_spr_spr0577                    MNDKLKIFLLLGVFFLAITGFYVLLIRNAGQTDASQIEKAAVSQGGKAVKKTEISKDADLHEIYLAGGCFWGVEEYFSRVPGVTDAVSGYANGRGETTKYELINQTGHAETVHVTYDAKQ
G54_spx_SPG_0601                  MNDKLKIFLLLGVFFLAITGFYVLLIRNAGQTDASQIEKAAVSQGGKAVKKTEISKDADLHEIYLAGGCFWGVEEYFSRVPGVTDAVSGYANGRGETTKYELINQTGHAETVHVTYDAKQ
CGSP14_SPCG_0616                  MNDKLKIFLLLGVFFLAITGFYVLLIRNAGQTDASQIEKAAVSQGGKAVKKTEISKDADLHEIYLAGGCFWGVEEYFSRVPGVTDAVSGYANGRGETTKYELINQTGHAETVHVTYDAKQ
ATCC700669_sne_SPN23F_05950       MNDKLKIFLLLGVFFLAITGFYVLLIRNAGQTDASQIEKAAVSQGGKAVKKTEISEDADLHEIYLAGGCFWGVEEYFSRVPGVTDAVSGYANGRGETTKYELINQTGHAETVHVTYDAKQ
Hungary19A_spv_SPH_0755           MNDKLKIFLLLGVFFLAITGFYVLLIRNAGQTDASQIEKAAVSQGGKAVKKTEISKDADLHEIYLAGGCFWGVEEYFSRVPGVTDAVSGYANGRGETTKYELINQTGHAETVHVTYDAKQ
JJA_sjj_SPJ_0609                  MNDKLKIFLLLGVFFLAITGFYVLLIRNAGQTDASQIEKAAVSQGGKAVKKTEISEDADLHEIYLAGGCFWGVEEYFSRVPGVTDAVSGYANGRGETTKYELINQTGHAETVHVTYDAKQ
670_6B_snb_SP670_0718             MNDKLKIFLLLGVFFLAITGFYVLLIRNAGQTDASQIEKAAVSQGGKAVKKTEISKDADLHEIYLAGGCFWGVEEYFSRVPGVTDAVSGYANGRGETTKYELINQTGHAETVHVTYDAKQ
P1031_spp_SPP_0680                MNDKLKIFLLLGVFFLAITGFYVLLIRNAGQTDASQIEKAAVSQGGKAVKKTEISKDADLHEIYLAGGCFWGVEEYFSRVPGVTDAVSGYANGRGETTKYELINQTGHAETVHVTYDAKQ
TCH8431/19A_snc_HMPREF0837_10952  MNDKLKIFLLLGVFFLAITGFYVLLIRNAGQTDASQIEKAAVSQGGKAVKKTEISEDADLHEIYLAGGCFWGVEEYFSRVPGVTDAVSGYANGRGETTKYELINQTGHAETVHVTYDAKQ
Taiwan19F_snt_SPT_0684            MNDKLKIFLLLGVFFLAITGFYVLLIRNAGQTDASQIEKAAVSQGGKTVKKTEISKDADLHEIYLAGGCFWGVEEYFSRVPGVTDAVSGYANGRGETTKYELINQTGHAETVHVTYDAKQ

TIGR4_spn_SP_0660                 ISLKEILLHYFRIINPTSKNKQGNDVGTQYRTGVYYTDDKDLEVINQVFDEVAKKYDQPLAVEKENLKNFVVAEDYHQDYLKKNPNGYCHINVNQAAYPVIDASKYPKPSDEELKKTLSP
D39_spd_SPD_0573                  ISLKEILLHYFRIINPTSKNKQGNDVGTQYRTGVYYTDDKDLEVINQVFDEVAKKYDQPLAVEKENLKNFVVAEDYHQDYLKKNPNGYCHINVNQAAYPVIDASKYPKPSDEELKKTLSP
R6_spr_spr0577                    ISLKEILLHYFRIINPTSKNKQGNDVGTQYRTGVYYTDDKDLEVINQVFDEVAKKYDQPLAVEKENLKNFVVAEDYHQDYLKKNPNGYCHINVNQAAYPVIDASKYPKPSDEELKKTLSP
G54_spx_SPG_0601                  ISLKEILLHYFRIINPTSKNKQGNDVGTQYRTGVYYTDDKDLEVINQVFDEVAKKYDQPLAVEKENLKNFVVAEDYHQDYLKKNPNGYCHINVNQAAYPVIDASKYPKPSDEELKKTLSP
CGSP14_SPCG_0616                  ISLKEILLHYFRIINPTSKNKQGNDVGTQYRTGVYYTDDKDLEVINQVFDEVAKKYDQPLAVEKENLKNFVVAEDYHQDYLKKNPNGYCHINVNQAAYPVIDASKYPKPSDEELKKTLSP
ATCC700669_sne_SPN23F_05950       ISLKEILLHYFRIINPTSKNKQGNDVGTQYRTGVYYTDDKDLEVINQVFDEVAKKYDQPLAVEKENLKNFVVAEDYHQDYLKKNPNGYCHINVNQAAYPVIDASKYPKPSDEELKKTLSP
Hungary19A_spv_SPH_0755           ISLKEILLHYFRIINPTSKNKQGNDVGTQYRTGVYYTDDKDLEVINQVFDEVAKKYDQPLAVEKENLKNFVVAEDYHQDYLKKNPNGYCHINVNQAAYPVIDASKYPKPSDEELKKTLSP
JJA_sjj_SPJ_0609                  ISLKEILLHYFRIINPTSKNKQGNDVGTQYRTGVYYTDDKDLEVINQVFDEVAKKYDQPLAVEKENLKNFVVAEDYHQDYLKKNPNGYCHINVNQAAYPVIDASKYPKPSDEELKKTLSP
670_6B_snb_SP670_0718             ISLKEILLHYFRIINPTSKNKQGNDVGTQYRTGVYYTDDKDLEVINQVFDEVAKKYDQPLAVEKENLKNFVVAEDYHQDYLKKNPNGYCHINVNQAAYPVIDASKYPKPSDEELKKTLSP
P1031_spp_SPP_0680                ISLKEILLHYFRIINPTSKNKQGNDVGTQYRTGVYYTDDKDLEVINQVFDEVAKKYDQPLAVEKENLKNFVVAEDYHQDYLKKNPNGYCHINVNQAAYPVIDASKYPKPSDEELKKTLSP
TCH8431/19A_snc_HMPREF0837_10952  ISLKEILLHYFRIINPTSKNKQGNDVGTQYRTGVYYTDDKDLEVINQVFDEVAKKYDQPLAVEKENLKNFVVAEDYHQDYLKKNPNGYCHINVNQAAYPVIDASKYPKPSDEELKKTLSP
Taiwan19F_snt_SPT_0684            ISLKEILLHYFRIINPTSKNKQGNDVGTQYRTGVYYTDDKDLEVINQVFDEVAKKYDQPLAVEKENLKNFVVAEDYHQDYLKKNPNGYCHINVNQAAYPVIDASKYPKPSDEELKKTLSP

TIGR4_spn_SP_0660                 EEYAVTQENQTERAFSNRYWDKFESGIYVDIATGEPLFSSKDKFESGCGWPSFTQPISPDVVTYKEDKSYNMTRMEVRSRVGDSHLGHVFTDGPQDKGGLRYCINSLSIRFIPKDQMEEK
D39_spd_SPD_0573                  EEYAVTQENQTERAFSNRYWDKFESGIYVDIATGEPLFSSKDKFESGCGWPSFTQPISPDVVTYKEDKSYNMTRMEVRSRVGDSHLGHVFTDGPQDKGGLRYCINSLSIRFIPKDQMEEK
R6_spr_spr0577                    EEYAVTQENQTERAFSNRYWDKFESGIYVDIATGEPLFSSKDKFESGCGWPSFTQPISPDVVTYKEDKSYNMTRMEVRSRVGDSHLGHVFTDGPQDKGGLRYCINSLSIRFIPKDQMEEK
G54_spx_SPG_0601                  EEYAVTQENQTERAFSNRYWDKFESGIYVDIATGEPLFSSKDKFESGCGWPSFTQPISPDVVTYKEDKSYNMTRMEVRSRVGDSHLGHVFTDGPQDKGGLRYCINSLSIRFIPKDQMEEK
CGSP14_SPCG_0616                  EEYAVTQENQTERAFSNRYWDKFESGIYVDIATGEPLFSSKDKFESGCGWPSFTQPISPDVVTYKEDKSYNMTRMEVRSRVGDSHLGHVFTDGPQDKGGLRYCINSLSIRFIPKDQMEEK
ATCC700669_sne_SPN23F_05950       EEYAVTQENQTERAFSNRYWDKFESGIYVDIATGEPLFSSKDKFESGCGWPSFTQPISPDVVTYKEDKSYNMTRMEVRSRVGDSHLGHVFTDGPQDKGGLRYCINSLSIRFIPKDQMEEK
Hungary19A_spv_SPH_0755           EEYAVTQENQTERAFSNRYWDKFESGIYVDIATGEPLFSSKDKFESGCGWPSFTQPISPDVVTYKEDKSYNMTRMEVRSRVGDSHLGHVFTDGPQDKGGLRYCINSLSIRFIPKDQMEEK
JJA_sjj_SPJ_0609                  EEYAVTQENQTERAFSNRYWDKFESGIYVDIATGEPLFSSKDKFESGCGWPSFTQPISPDVVTYKEDKSYNMTRMEVRSRVGDSHLGHVFTDGPQDKGGLRYCINSLSIRFIPKDQMEEK
670_6B_snb_SP670_0718             EEYAVTQENQTERAFSNRYWDKFESGIYVDIATGEPLFSSKDKFESGCGWPSFTQPISPDVVTYKEDKSYNMTRMEVRSRVGDSHLGHVFTDGPQDKGGLRYCINSLSIRFIPKDQMEEK
P1031_spp_SPP_0680                EEYAVTQENQTERAFSNRYWDKFESGIYVDIATGEPLFSSKDKFESGCGWPSFTQPISPDVVTYKEDKSYNMTRMEVRSRVGDSHLGHVFTDGPQDKGGLRYCINSLSIRFIPKDQMEEK
TCH8431/19A_snc_HMPREF0837_10952  EEYAVTQENQTERAFSNRYWDKFESGIYVDIATGEPLFSSKDKFESGCGWPSFTQPISPDVVTYKEDKSYNMTRMEVRSRVGDSHLGHVFTDGPQDKGGLRYCINSLSIRFIPKDQMEEK
Taiwan19F_snt_SPT_0684            EEYAVTQENQTERAFSNRYWDKFESGIYVDIATGEPLFSSKDKFESGCGWPSFTQPISPDVVTYKEDKSYNMTRMEVRSRVGDSHLGHVFTDGPQDKGGLRYCINSLSIRFIPKDQMEEK

TIGR4_spn_SP_0660                 GYAYLLDYVD
D39_spd_SPD_0573                  GYAYLLDYVD
R6_spr_spr0577                    GYAYLLDYVD
G54_spx_SPG_0601                  GYAYLLDYVD
CGSP14_SPCG_0616                  GYAYLLDYVD
ATCC700669_sne_SPN23F_05950       GYAYLLDYVD
Hungary19A_spv_SPH_0755           GYAYLLDYVD
JJA_sjj_SPJ_0609                  GYAYLLDYVD
670_6B_snb_SP670_0718             GYAYLLDYVD
P1031_spp_SPP_0680                GYAYLLDYVD
TCH8431/19A_snc_HMPREF0837_10952  GYAYLLDYVD
Taiwan19F_snt_SPT_0684            GYAYLLDYVD
```

Figure S4. Comparison of MsrAB2 protein sequences of *S. pneumoniae* as deposited in databases for 12 pneumococcal strains.

Figure S5

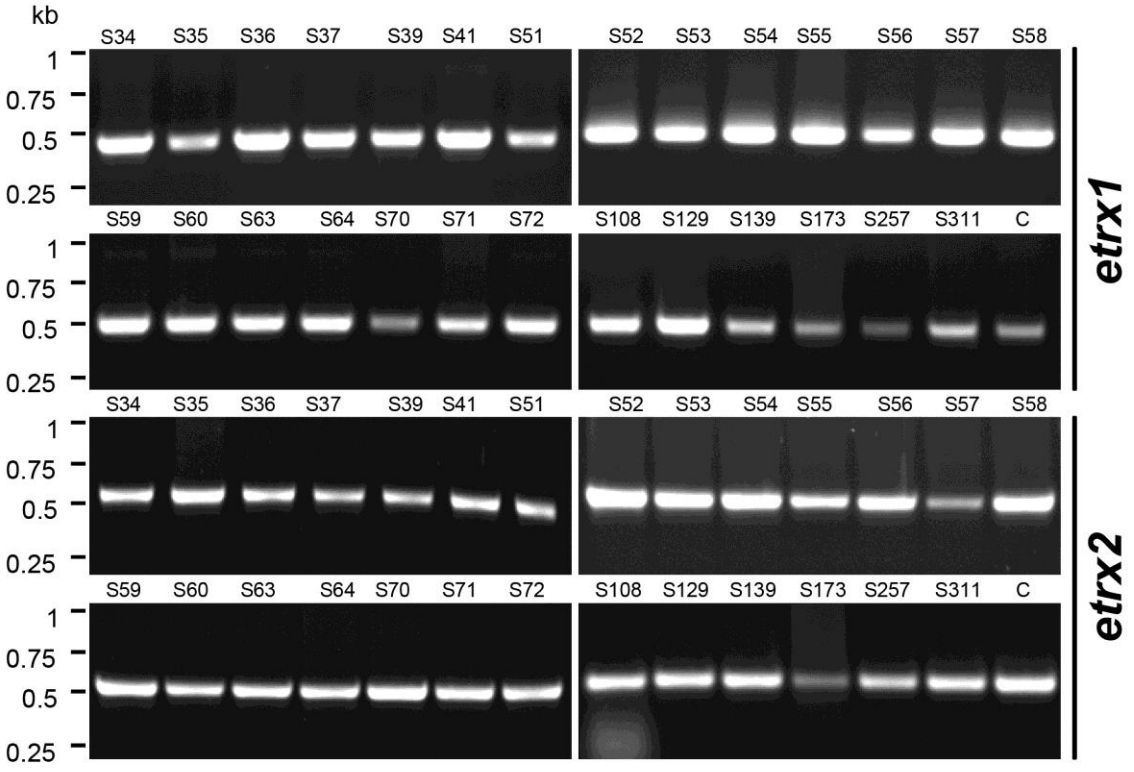

Figure S5. Conservation and distribution of *etrx* genes in pneumococci as analyzed by PCR. Gene fragments of *etrx1* and *etrx2* were amplified by PCR using specific primer pairs etrx1_447/ etrx1_448 and etrx2_486/ etrx2_487, respectively.

Figure S6

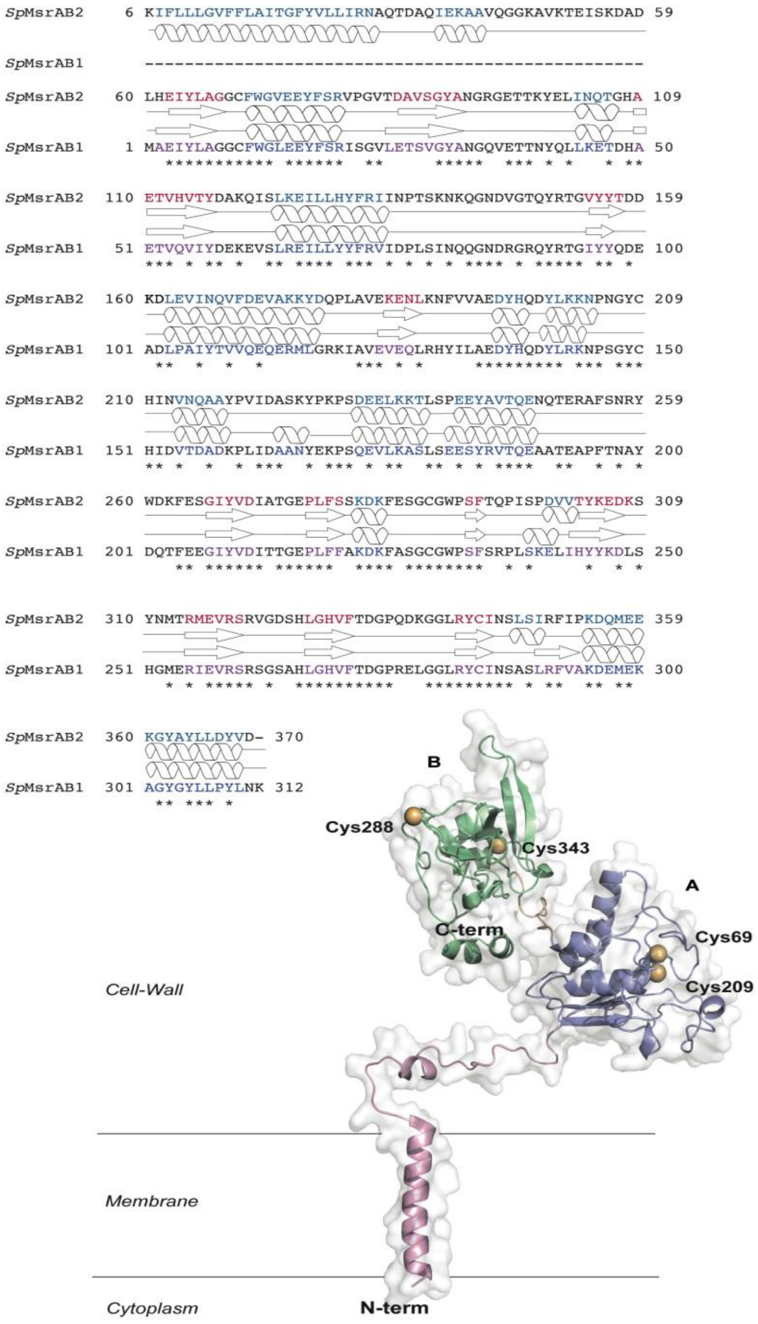

Figure S6. Sequence alignment between pneumococcal cytoplasmic *Sp*MsrAB1 and surface-exposed *Sp*MsrAB2. Secondary structural elements are also drawn. Conserved residues are highlighted with asterisk. On the bottom, the putative three-dimensional model is drawn in a ribbon representation. The MsrA domain is shown in blue and the MsrB in green, the transmembrane and coiled coil regions are colored in magenta. Catalytic cysteines are labeled and represented as spheres. Sequence homology for D39 *Sp*MsrAB1 (SPD_1193) and *Sp*MsrAB2 (SPD_0573) is 77%.

Figure S7

```
TIGR4_spn_SP_0999                  METIVFLISVFLAGVLSFFSPCIFPLLPVYAGILLDDQESAKSFSLFGRKVLWSGLIRTLCFIAGISLIFFILGFGAGYFGHILYANWFRYGMGAIIIILGLHQMEIFHLKKLEVQKSFT
D39_spd_SPD_0885                   METIVFLISVFLAGVLSFFSPCIFPLLPVYAGILLDDQESAKSFSLFGRKVLWSGLIRTLCFIAGISLIFFILGFGAGYFGHILYANWFRYVMGAIIIILGLHQMEIFHLKKLEVQKSFT
R6_spr_spr0903                     METIVFLISVFLAGVLSFFSPCIFPLLPVYAGILLDDQESAKSFSLFGRKVLWSGLIRTLCFIAGISLIFFILGFGAGYFGHILYANWFRYVMGAIIIILGLHQMEIFHLKKLEVQKSFT
G54_spx_SPG_0924                   METIVFLISVFLAGVLSFFSPCIFPLLPVYAGILLDDQESAKSFSLFGRKVLWSGLIRTLCFIAGISLIFFILGFGAGYFGHILYANWFRYGMGAIIIILGLHQMEIFHLKKLEVQKSFT
CGSP14_spw_SPCG_0973               METIVFLISVFLAGVLSFFSPCIFPLLPVYAGILLDDQESAKSFSLFGRKVLWSGLIRTLCFIAGISLIFFILGFGAGYFGHILYANWFRYVMGAIIIILGLHQMEIFHLKKLEVQKSFT
ATCC700669_sne_SPN23F_09250        METIVFLISVFLAGVLSFFSPCIFPLLPVYAGILLDDQESAKSFSLFGRKVLWSGLIRTLCFIAGISLIFFILGFGAGYFGHILYANWFRYGMGAIIIILGLHQMEIFHLKKLEVQKSFT
Hungary19A_spv_SPH_1100            METIVFLISVFLAGVLSFFSPCIFPLLPVYAGILLDDQESAKSFSLFGRKVLWSGLIRTLCFIAGISLIFFILGFGAGYFGHILYANWFRYVMGAIIIILGLHQMEIFHLKKLEVQKSFT
JJA_sjj_SPJ_0940                   METIVFLISVFLAGVLSFFSPCIFPLLPVYAGILLDDQESAKSFSLFGRKVLWSGLIRTLCFIAGISLIFFILGFGAGYFGHILYANWFRYGMGAIIIILGLHQMEIFHLKKLEVQKSFT
670_6B_snb_SP670_1322              METIVFLISVFLAGVLSFFSPCIFPLLPVYAGILLDDQESAKSFSLFGRKVLWSGLIRTLCFIAGISLIFFILGFGAGYFGHILYANWFRYGMGAIIIILGLHQMEIFHLKKLEVQKSFT
P1031_spp_SPP_1005                 METIVFLISVFLAGVLSFFSPCIFPLLPVYAGILLDDQESAKSFSLFGRKVLWSGLIRTLCFIAGISLIFFILGFGAGYFGHILYANWFRYGMGAIIIILGLHQMEIFHLKKLEVQKSFT
TCH8431/19A_snc_HMPREF0837_11485   METIVFLISVFLAGVLSFFSPCIFPLLPVYAGILLDDQESAKSFSLFGRKVLWSGLIRTLCFIAGISLIFFILGFGAGYFGHILYANWFRYGMGAIIIILGLHQMEIFHLKKLEVQKSFT
Taiwan19F_snt_SPT_1202             METIVFLISVFLAGVLSFFSPCIFPLLPVYAGILLDDQESAKSFSLFGRKVLWSGLIRTLCFIAGISLIFFILGFGAGYFGHILYANWFRYGMGAIIIILGLHQMEIFHLKKLEVQKSFT
```

```
TIGR4_spn_SP_0999                  FKKSDSNRYWSAFLLGITFSFGWTPCIGPVLSSVLALAASGGNGAWQGAIYTLIYTLGMALPFLVLALASGLVMPYFSKIKRHMMLLKKIGGFLIVLMGILLLLGQVNVLAGIFE
D39_spd_SPD_0885                   FKKSDSNRYWSAFLLGITFSFGWTPCIGPVLSSVLALAASGGNGAWQGAIYTLIYTLGMALPFLVLALASGLVMPYFSKIKRHMMLLKKIGGFLIVLMGILLLLGQVNVLAGIFE
R6_spr_spr0903                     FKKSDSNRYWSAFLLGITFSFGWTPCIGPVLSSVLALAASGGNGAWQGAIYTLIYTLGMALPFLVLALASGLVMPYFSKIKRHMMLLKKIGGFLIVLMGILLLLGQVNVLAGIFE
G54_spx_SPG_0924                   FKKSDSNRYWSAFLLGITFSFGWTPCIGPVLSSVLALAASGGNGAWQGAIYTLIYTLGMALPFLVLALASGLVMPYFSKIKRHMMLLKKIGGFLIVLMGILLLLGQVNVLAGIFE
CGSP14_spw_SPCG_0973               FKKSDSNRYWSAFLLGITFSFGWTPCIGPVLSSVLALAASGGNGAWQGAIYTLIYTLGMALPFLVLALASGLVMPYFSKIKRHMMLLKKIGGFLIVLMGILLLLGQVNVLAGIFE
ATCC700669_sne_SPN23F_09250        FKKSDSNRYWSAFLLGITFSFGWTPCIGPVLSSVLALAASGGNGAWQGAIYTLIYTLGMALPFLVLALASGLVMPYFSKIKRHMMLLKKIGGFLIVLMGILLLLGQVNVLAGIFE
Hungary19A_spv_SPH_1100            FKKSDSNRYWSAFLLGITFSFGWTPCIGPVLSSVLALAASGGNGAWQGAIYTLIYTLGMALPFLVLALASGLVMPYFSKIKRHMMLLKKIGGFLIVLMGILLLLGQVNVLAGIFE
JJA_sjj_SPJ_0940                   FKKSDSNRYWSAFLLGITFSFGWTPCIGPVLSSVLALAASGGNGAWQGAIYTLIYTLGMALPFLVLALASGLVMPYFSKIKRHMMLLKKIGGFLIVLMGILLLLGQVNVLAGIFE
670_6B_snb_SP670_1322              FKKSDSNRYWSAFLLGITFSFGWTPCIGPVLSSVLALAASGGNGAWQGAIYTLIYTLGMALPFLVLALASGLVMPYFSKIKRHMMLLKKIGGFLIVLMGILLLLGQVNVLAGIFE
P1031_spp_SPP_1005                 FKKSDSNRYWSAFLLGITFSFGWTPCIGPVLSSVLALAASGGNGAWQGAIYTLIYTLGMALPFLVLALASGLVMPYFSKIKRHMMLLKKIGGFLIVLMGILLLLGQVNVLAGIFE
TCH8431/19A_snc_HMPREF0837_11485   FKKSDSNRYWSAFLLGITFSFGWTPCIGPVLSSVLALAASGGNGAWQGAIYTLIYTLGMALPFLVLALASGLVMPYFSKIKRHMMLLKKIGGFLIVLMGILLLLGQVNVLAGIFE
Taiwan19F_snt_SPT_1202             FKKSDSNRYWSAFLLGITFSFGWTPCIGPVLSSVLALAASGGNGAWQGAIYTLIYTLGMALPFLVLALASGLVMPYFSKIKRHMMLLKKIGGFLIVLMGILLLLGQVNVLAGIFE
```

Figure S7. Comparison of CcdA2 protein sequences of *S. pneumoniae* as deposited in databases for 12 pneumococcal strains.

Figure S8

```
TIGR4_spn_SP_1000                  MKKVMFAGLSLLSLVVLMACGEEETKKTQAAQQPKQQTTVQQIAVGKDAPDFTLQSMDGKEVKLSDFKGKKVYLKFWASWCGPCKKSMPELMELAAKPDRDFEILTVIAPGIQG
D39_spd_SPD_0886                   MKKVMFAGLSLLSLVVLMACGEEETKKTQAAQQPKQQTTVQQISVGKDVPDFTLQSMDGKEVKLSDFKGKKVYLKFWASWCGPCKKSMPELMELAAKPDRDFEILTVIAPGIQG
R6_spr_spr0904                     MKKVMFAGLSLLSLVVLMACGEEETKKTQAAQQPKQQTTVQQISVGKDVPDFTLQSMDGKEVKLSDFKGKKVYLKFWASWCGPCKKSMPELMELAAKPDRDFEILTVIAPGIQG
G54_spx_SPG_0925                   MKKVMFAGLSLLSLVVLMACGQEETKKTQAAQQPKQQTTVQQISVGKDAPDFTLQSMDGKEVKLSDFKGKKVYLKFWASWCGPCKKSMPELMELAAKPDRDFEILTVIAPGIQG
CGSP14_spw_SPCG_0974               MKKVMFAGLSLLSLVVLMACGEEETKKTQAAQQPKQQTTVQQISVGKDVPDFTLQSMDGKEVKLSDFKGKKVYLKFWASWCGPCKKSMPELMELAAKPDRDFEILTVIAPGIQG
ATCC700669_sne_SPN23F_09260        MKKVMFAGLSLLSLVVLMACGEEETKKTQAAQQPKQQTTVQQIAVGKDAPNFTLQSMDGKEVKLSDFKGKKVYLKFWASWCGPCKKSMPELMELAAKPDRDFEILTVIAPGIQG
Hungary19A_spv_SPH_1101            MKKVMFAGLSLLSLVVLMACGEEETKKTQAAQQPKQQTTVQQISVGKDVPDFTLQSMDGKEVKLSDFKGKKVYLKFWASWCGPCKKSMPELMELAAKPDRDFEILTVIAPGIQG
JJA_sjj_SPJ_0941                   MKKVMFAGLSLLSLVVLMACGEEETKKTQAAQQPKQQTTVQQIAVGKDAPDFTLQSMDGKEVKLSDFKGKKVYLKFWASWCGPCKKSMPELMELAAKPDRDFEILTVIAPGIQG
670_6B_snb_SP670_1321              MKKVMFAGLSLLSLVVLMACGEEETKKTQAAQQPKQQTTVQQIAVGKDAPDFTLQSMDGKEVKLSDFKGKKVYLKFWASWCGPCKKSMPELMELAAKPDRDFEILTVIAPGIQG
P1031_spp_SPP_1006                 MKKVMFAGLSLLSLVVLMACGEEETKKTQAAQQPKQQTTVQQIAVGKDASNFTLQSMDGKEVKLSDFKGKKVYLKFWASWCGPCKKSMPELMELAAKPDRDFEILTVIAPGIQG
TCH8431/19A_snc_HMPREF0837_11484   MKKVMFAGLSLLSLVVLMACGEEETKKTQAAQQPKQQTTVQQIAVGKDAPNFTLQSMDGKEVKLSDFKGKKVYLKFWASWCGPCKKSMPELMELAAKPDRDFEILTVIAPGIQG
Taiwan19F_snt_SPT_1201             MKKVMFAGLSLLSLVVLMACGEEETKKTQAAQQPKQQTTVQQIAVGKDAPDFTLQSMDGKEVKLSDFKGKKVYLKFWASWCGPCKKSMPELMELAAKPDRDFEILTVIAPGIQG

TIGR4_spn_SP_1000                  EKTVEQFPQWFQEQGYKDIPVLYDTKATTFQAYQIRSIPTEYLIDSQGKIGKIQFGAISNADAEAAFKEMN
D39_spd_SPD_0886                   EKTVEQFPQWFQEQGYKDIPVLYDTKATTFQAYQIRSIPTEYLIDSQGKIGKIQFGAISNADAEAAFKEMN
R6_spr_spr0904                     EKTVEQFPQWFQEQGYKDIPVLYDTKATTFQAYQIRSIPTEYLIDSQGKIGKIQFGAISNADAEAAFKEMN
G54_spx_SPG_0925                   EKTVEQFPQWFQEQGYKDIPVLYDTKATTFQAYQIRSIPTEYLIDSQGKIGKIQFGAISNADAEAAFKEMN
CGSP14_spw_SPCG_0974               EKTVEQFPQWFQEQGYKDIPVLYDTKATTFQAYQIRSIPTEYLIDSQGKIGKIQFGAISNADAEAAFKEMN
ATCC700669_sne_SPN23F_09260        EKIVEQFPQWFQEQGYKDIPVLYDTKATTFQAYQIRSIPTEYLIDSQGKIGKIQFGAISNADAEAAFKEMN
Hungary19A_spv_SPH_1101            EKTVEQFPQWFQEQGYKDIPVLYDTKATTFQAYQIRSIPTEYLIDSQGKIGKIQFGAISNADAEAAFKEMN
JJA_sjj_SPJ_0941                   EKTVEQFPQWFQEQGYKDIPVLYDTKATTFQAYQIRSIPTEYLIDSQGKIGKIQFGAISNADAEAAFKEMN
670_6B_snb_SP670_1321              EKTVEQFPQWFQEQGYKDIPVLYDTKATTFQAYQIRSIPTEYLIDSQGKIGKIQFGAISNADAEAAFKEMN
P1031_spp_SPP_1006                 EKTVEQFPQWFQEQGYKDIPVLYDTKATTFQAYQIRSIPTEYLIDSQGKIGKIQFGAISNADAETAFKEMN
TCH8431/19A_snc_HMPREF0837_11484   EKIVEQFPQWFQEQGYKDIPVLYDTKATTFQAYQIRSIPTEYLIDSQGKIGKIQFGAISNADAEAAFKEMN
Taiwan19F_snt_SPT_1201             EKTVEQFPQWFQEQGYKDIPVLYDTKATTFQAYQIRSIPTEYLIDSQGKIGKIQFGAISNADAEAAFKEMN
```

Figure S8. Comparison of Etrx2 protein sequences of *S. pneumoniae* as deposited in databases for 12 pneumococcal strains.

```
CCdA1           MGHIFFFLSVFLAGILSFFSPCILPLLPVYTGVLLDDKDGAQASSGKFS--ISVTSLLRT 58
CcdA2           METIVFLISVFLAGVLSFFSPCIFPLLPVYAGILLDDQESAKSFS-LFGRKVLWSGLIRT 59
                *  *.*::******:********:******:*:****::.*:: *  *.  :  :.*:**

CcdA1           LAFIAGISFIFILLGYGAGFLGDLLYASWFQYLTGAIIILLGLHQMEILHFKGLYKEKRL 118
CcdA2           LCFIAGISLIFFILGFGAGYFGHILYANWFRYVMGAIIIILGLHQMEIFHLKKLEVQKSF 119
                *.******:**::**:***::*.:***.**:*: *****:********:*:* *  :* :

CcdA1           QLQGQGQNGKGYSQAFLLGLTFSFAWTPCVGPVLGSVLALAASGGSGAWQGAGLMLVYTL 178
CcdA2           TFKKSDSN--RYWSAFLLGITFSFGWTPCIGPVLSSVLALAASGGNGAWQGAIYTLIYTL 177
                 :: ...*   * .*****:****.****:****.**********.******   *:***

CcdA1           GLALPFLLLALTSSYVLKHFRKLHPYLGILKKVGGFLIIVMGFLVLFGNASILSQLFE 236
CcdA2           GMALPFLVLALASGLVMPYFSKIKRHMMLLKKIGGFLIVLMGILLLLGQVNVLAGIFE 235
                *:*****:***:*. *: :* *:: :: :***:*****::**:*:*:*:..:*: :**
```

Figure S9. **Sequence alignment between *S. pneumoniae* D39 CcdA1 (SPD_0571) and CcdA2 (SPD_0885).** The conserved residues are marked with asterisk. Protein sequences showed an identity of 58.8%.

Figure S10

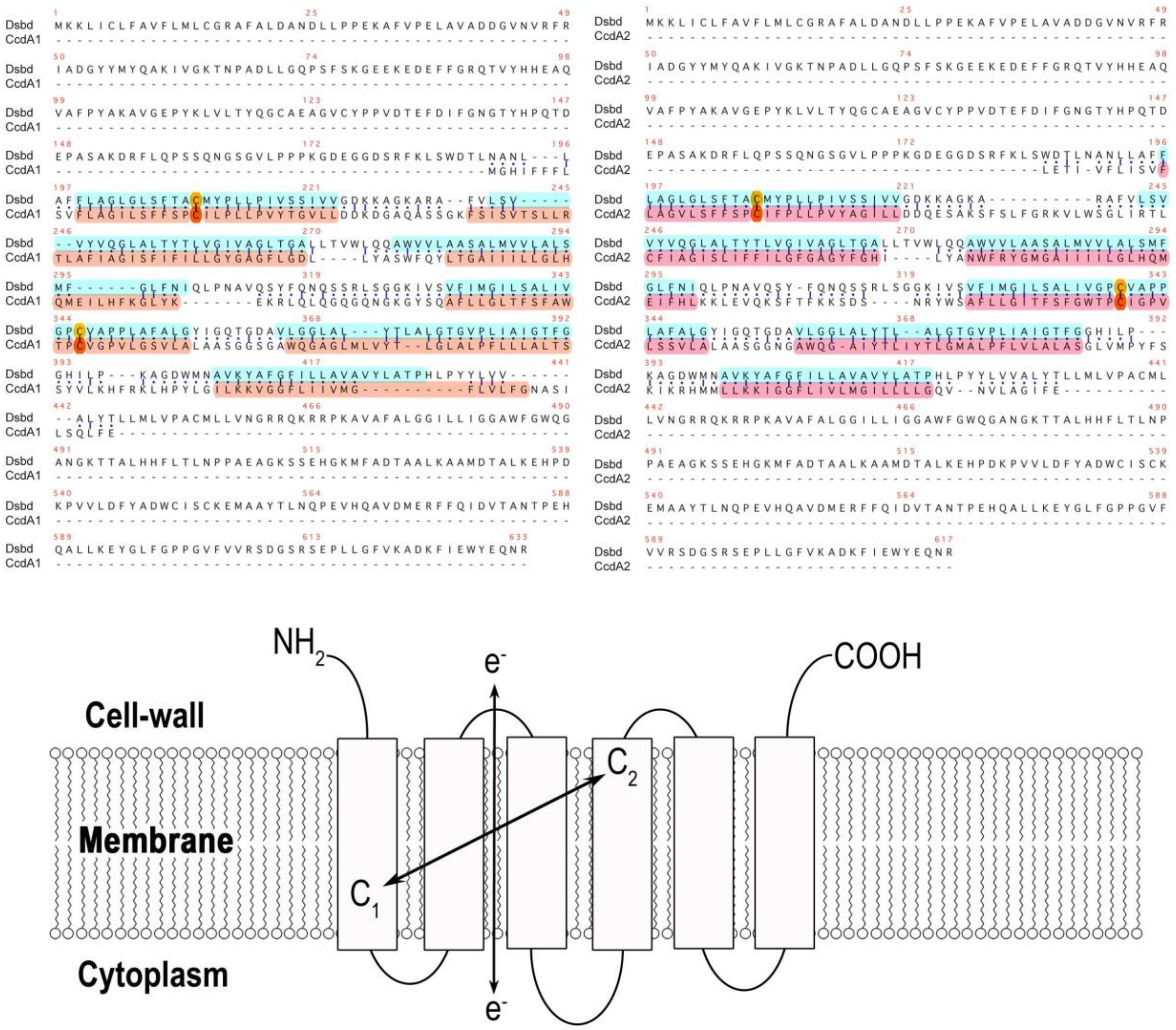

Figure S10. **Sequence analysis between *S. pneumoniae* CcdA proteins and DsbD of *N. meningitidis*.** CcdA1 (SPD_0571) (left) and CcdA2 (SPD_0885) (right) of *S. pneumoniae* D39 were aligned with the *N. meningitidis* DsbD protein. The conserved transmembrane regions are coloured. Hypothetical transfer cysteines are highlighted in red or orange in the *S. pneumoniae* and *N. meningitidis* proteins, respectively. The disposition of transmembrane segments is shown.

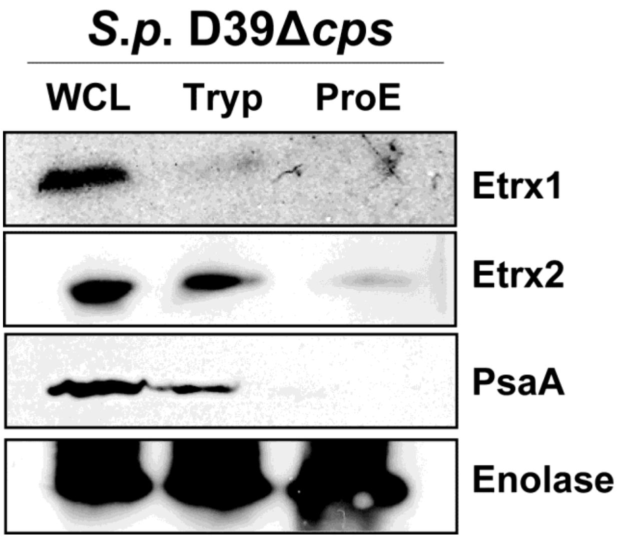

Figure S11. Abundance of Etrx and *Sp*MsrAB proteins after proteolytic treatment of pneumococci.

Pneumococci were grown in THY medium, harvested at OD_{600} of 0.3 and resuspended in 1 ml PBS pH 7.4 supplemented with 1% choline chloride to avoid autolysis. The proteolytic digest of surface-exposed proteins was carried out by treatment of 10^{10} pneumococci with trypsin (Tryp; Sigma; 1 mg/ml) or pronase E (ProE; Merck; 1 mg/ml). The untreated whole pneumococcal cell lysate was used as control (WCL). After 1 h at 37°C the untreated and trypsin or pronase E treated bacteria were sedimented, resuspended in SDS sample buffer, and proteolytic enzymes were inactivated at 95°C, which also lysed the bacteria. Proteins were separated by SDS-PAGE and immunoblotting was performed. The cytoplasmic enolase and the surface-exposed lipoprotein PsaA were used as controls (Bergmann et al, 2003; Johnston et al, 2004). The results revealed that proteolytic treatment of intact pneumococci reduces the abundance of Etrx1, Etrx2, and PsaA, while the abundance of cytoplasmic proteins such as the enolase was not affected. This confirms that Etrx1 and Etrx2 are surface-exposed proteins.

Figure S12

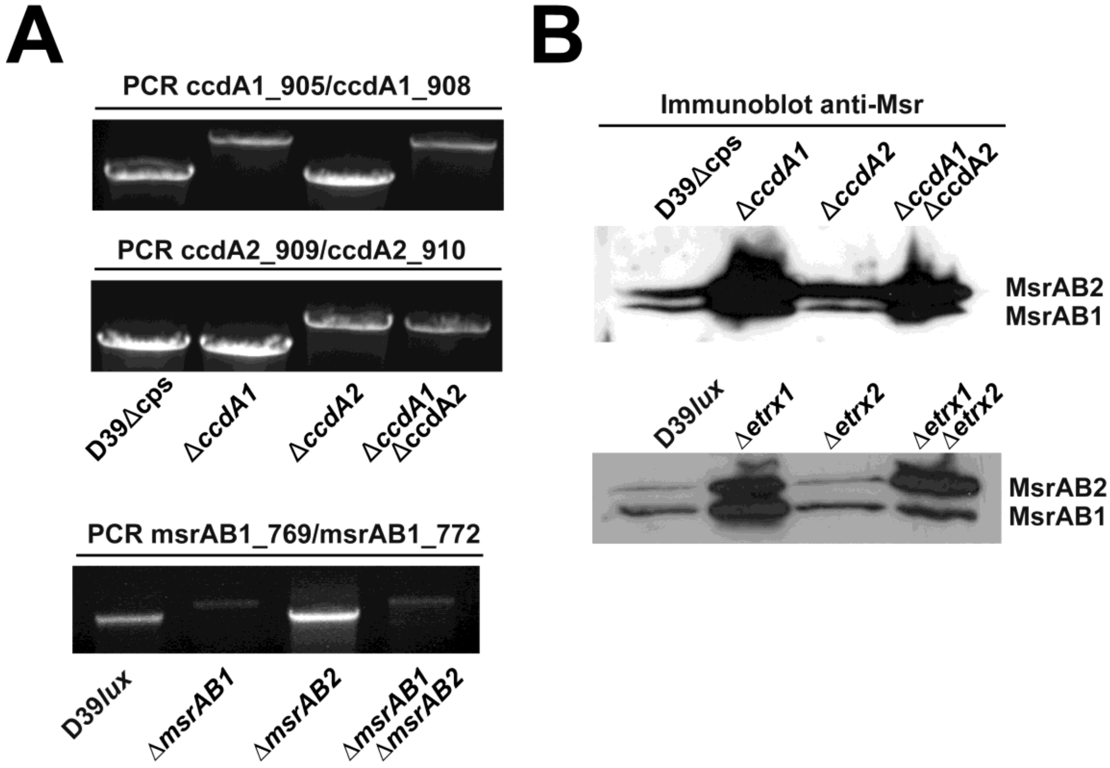

Figure S12. Molecular analysis of *ccdA-*, *etrx-* and *msrAB1*-mutants.

A. PCR analysis of pneumococcal mutants generated by allelic replacement. The mutants were verified by the size of the PCR product, which was larger compared to the PCR product obtained from wild-type DNA.

B. Production of MsrAB2 (and MsrAB1) in *ccdA*-, and *etrx*-mutants was confirmed by immunoblot analysis using mouse anti-MsrAB specific antibodies. The deficiency of CcdA1 or Etrx1 resulted in a higher production of MsrAB2, irrespective of the direction of the antibiotic gene cassette in the gene locus.

Figure S13

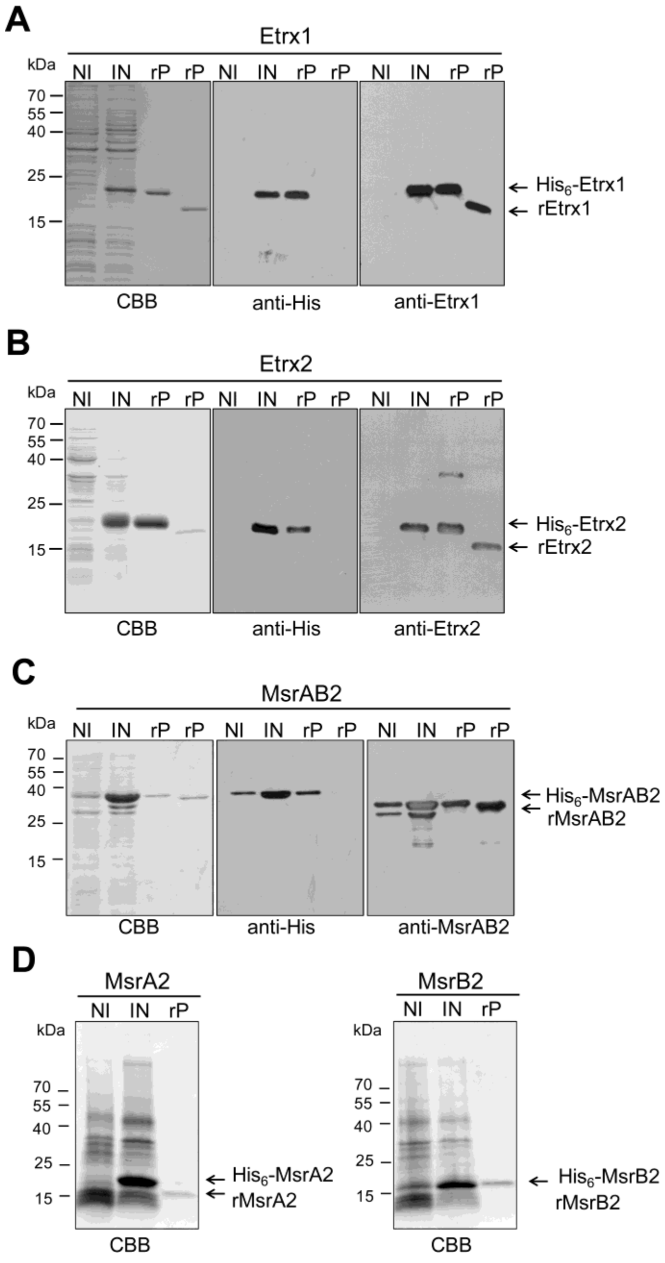

Figure S13. Purification and immunoblot analysis of recombinant Etrx proteins.

Etrx1 (**A**), Etrx2 (**B**), *Sp*MsrAB2 (**C**), MsrA2 (**D**), and MsrB2 (**D**) protein expression was induced with IPTG and total protein lysates were subjected to SDS-PAGE followed by Coomassie Brilliant Blue (CBB) staining. The His_6-tagged proteins (His_6-Etrx1 and His_6-Etrx2, His_6-MsrAB2, His_6-MsrA2, His_6-MsrB2) were purified by affinity chromatography and the His_6-tag was removed using TEV protease. The proteins were detected by immunoblot analysis using anti-Histidine, anti-Etrx or anti-MsrAB2 antibodies. NI, non-induced; IN, induced; rP, recombinant protein.

Figure S14

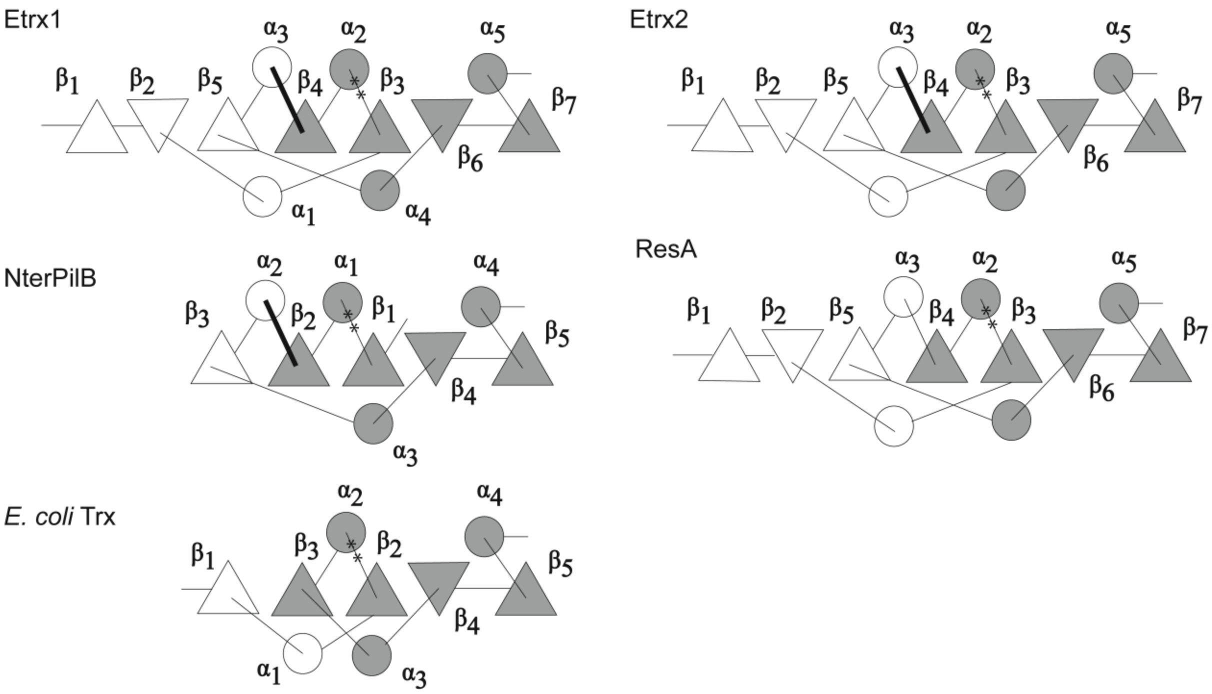

Figure S14. Topology diagrams of the Etrx1 and Etrx2 structural elements in comparison with domain 1 of PilB (NterPilB), ResA and the *E. coli* Trx proteins fold. Canonical thioredoxin-fold is shown in light grey in all cases while the approximate positions of catalytic and the resolving cysteine residues are shown as asterisks.

Figure S15

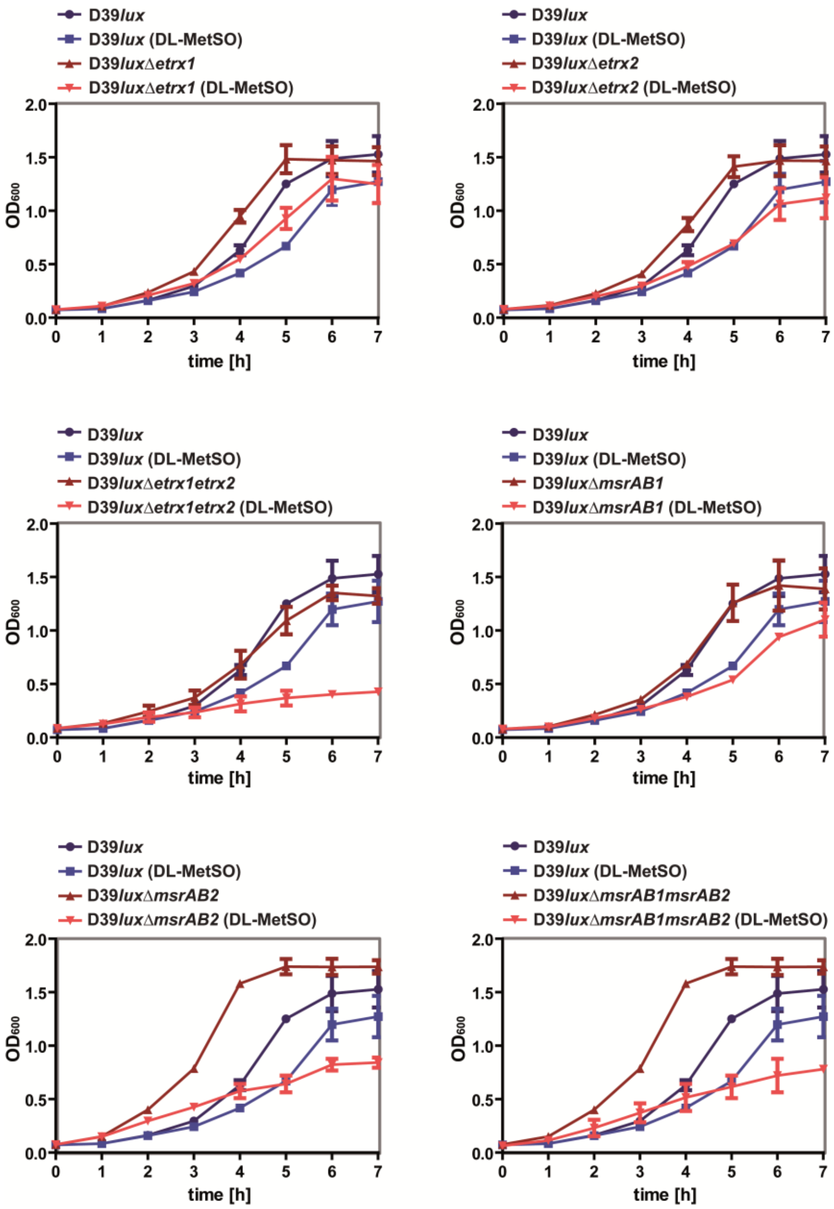

Figure S15. Influence of Etrx and MsrAB2 on pneumococcal growth in the presence of DL-methionine sulfoxide. The pneumococcal strain D39*lux* and its isogenic pneumococcal *etrx*-mutants and *msrAB*-mutants were cultured in THY supplemented with 6 mM DL-methionine sulfoxide (MetSO) and the OD_{600} was measured continuously. In the absence of both Etrx proteins or MsrAB2 protein MetSO impairs pneumococcal growth compared to the isogenic wild-type.

Figure S16

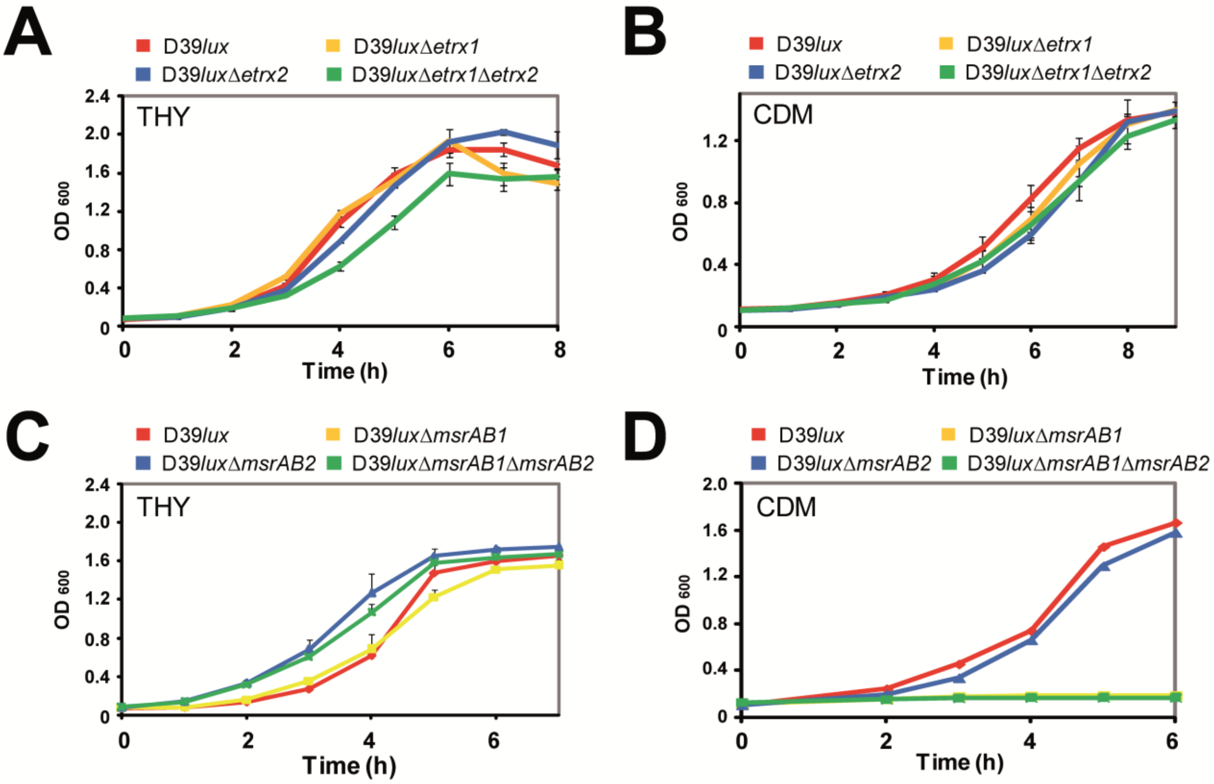

Figure S16. Growth curves of *S. pneumoniae* wild-type strains D39*lux* and its isogenic pneumococcal *etrx*- and *msrAB*-mutants. The wild-type D39*lux* and the mutants D39*lux*Δ*etrx1*, D39*lux*Δ*etrx2*, D39*lux*Δ*etrx1*Δ*etrx2*, D39*lux*Δ*msrAB1*, D39*lux*Δ*msrAB2*, D39*lux*Δ*msrAB1*Δ*msrAB2* were grown at 37°C and 5% CO_2 in complex THY media with erythromycin (2.5 µg/ml) (**A** and **C**) or in chemical defined medium CDM (**B** and **D**), respectively. The growth curves in THY and CDM indicated similar growth behaviour of the D39*lux*Δ*etrx1*Δ*etrx2* mutant compared to the wild-type and individual *etrx*-mutants. Similarly, growth of the *msrAB2*-mutant was not changed, while the deficiency of MsrAB1 severely affected bacterial fitness in CDM.

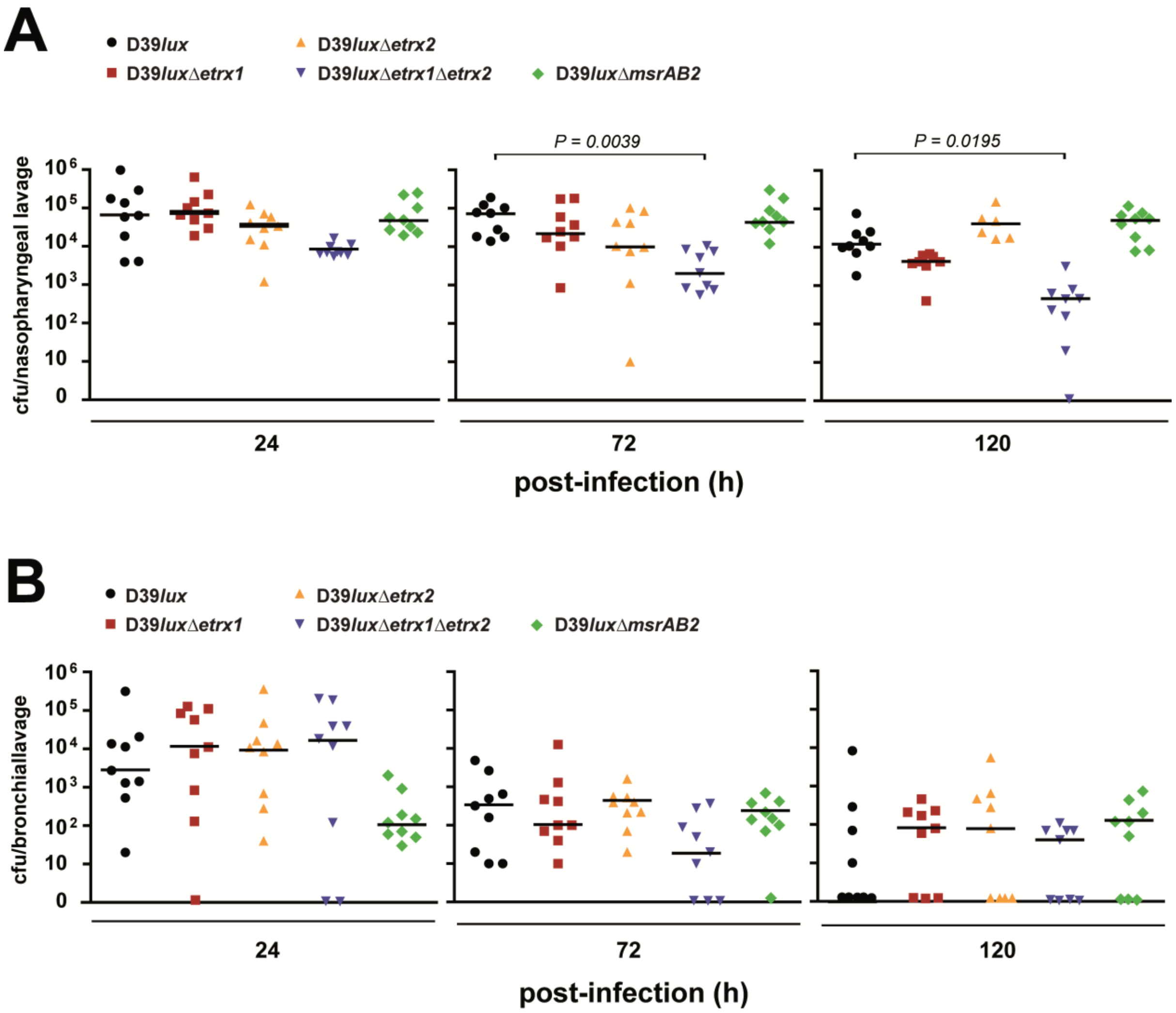

Figure S17. Influence of Etrx and MsrAB2 on pneumococcal colonization of the mouse nasopharynx.

A. Bacterial load in the nasopharynx.

B. Bacterial load in the bronchoalveolar lavage. Groups of CD-1 mice (n=9) were infected intranasally with 1x 10^6 CFU of D39*lux* or its isogenic *etrx-* and *msrAB2* mutants, respectively. One, 3, and 5 days post-infection the bacteria were recovered by a nasopharyngeal (**A**) and bronchoalveolar (**B**) wash. The bacteria were plated on blood agar and after overnight incubation at 37°C, 5% CO_2 the CFU were determined. Results are presented as single values and medians.

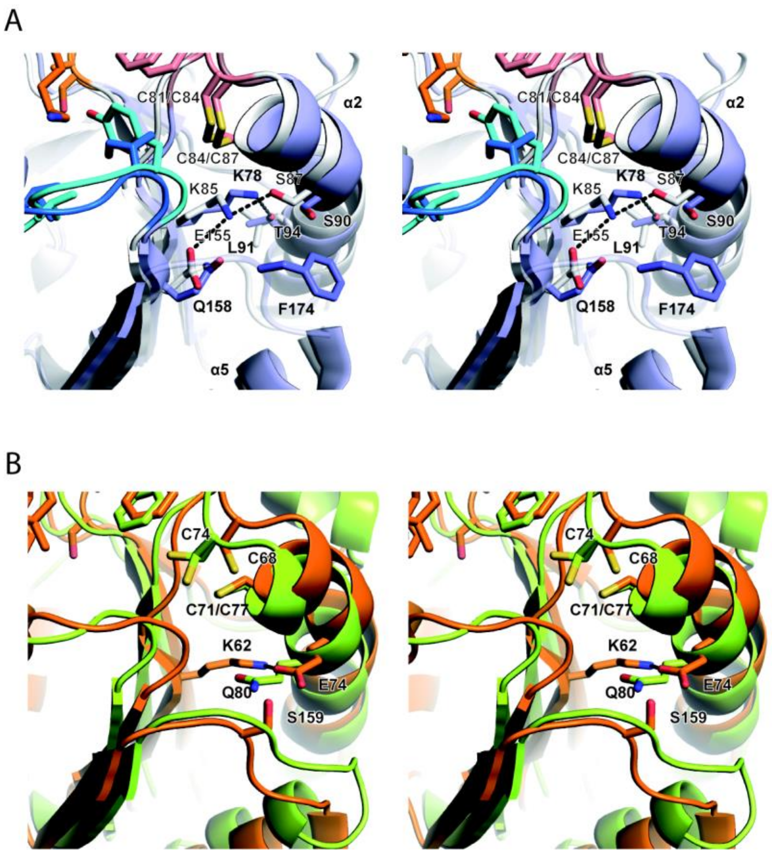

Figure S18. Stereo view of the catalytic core of pneumococcal Etrx proteins.

A. Etrx1 (light blue) and Etrx2 (white) superimposition. Relevant residues are labeled and shown as capped sticks. β4-α3 loop is colored in orange while the active CXXC region is colored in pink.

B. NterPilB (orange) and ResA (green) superimposition. Relevant residues are labeled and shown as capped sticks. Polar interactions are shown as dashed lines.

Supplemental Movie 1

Extracellular oxidative stress resistance mechanism of *Streptococcus pneumoniae*

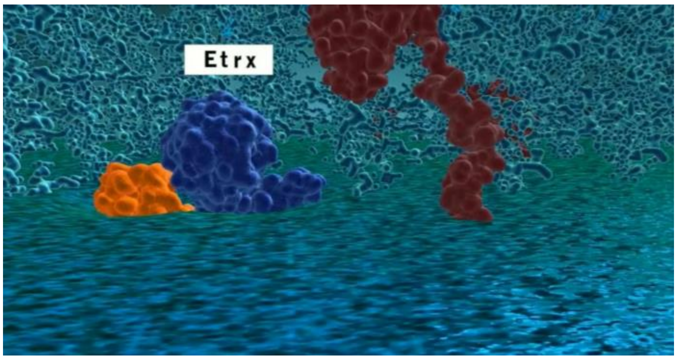

Table S1. Strain and plasmid list

Strain or plasmid	Serotype and relevant Genotype[a]	Resistance	Source or Reference
Streptococcus pneumoniae			
P34	1	None	ATTC 33400
P35	2	None	NCTC 7466
P36	3	None	NCTC 7978
P37	35A	None	NCTC 10319
P39	3	None	ATCC 6303
P41	5	None	ATCC 6305
P51	2	None	ATCC 11733
P52	1	None	ATCC 12213
P53	1	None	Statens Serum Institute
P54	3	None	Statens Serum Institute
P55	4	None	Statens Serum Institute
P56	6A	None	Statens Serum Institute
P57	6B	None	Statens Serum Institute
P58	8	None	Statens Serum Institute
P59	7F	None	Statens Serum Institute
P60	9V	None	Statens Serum Institute
P63	19F	None	Statens Serum Institute
P64	23F	None	Statens Serum Institute
P70	12F	None	MUD[b]
P71	7B	None	MUD[b]
P72	8B	None	MUD[b]
P108	19F	None	MUD[b]
P129	19F	None	MUD[b]
P139 (R6)	nonencapsulated derivative of D39	None	(Tomasz & Hotchkiss, 1964)
P173 (R800)	nonencapsulated derivative of R36A	None	(Holmes et al, 2001)
P257 (D39)	2	None	NCTC7466
P261 (TIGR4)	4	None	(Tettelin et al, 2001)
PN111	D39Δ*cps*	Km^r	(Jensch et al, 2010)
PN149	D39*lux*	Km^r	This work
PN220	D39Δ*cps*Δ*lgt*	Km^r, Erm^r	(Voss et al, 2013)
PN253	D39Δ*cps*Δ*spd_0572(etrx1)*	Km^r, Erm^r	This work
PN280	D39Δ*cps*Δ*spd_0886(etrx2)*	Km^r, Erm^r	This work
PN371	D39Δ*cps*Δ*spd_0572*Δ*spd_0886*	Km^r, Erm^r, Spec^r	This work
PN247	D39*lux*Δ*spd_0572(etrx1)*	Km^r, Erm^r	This work
PN285	D39*lux*Δ*spd_0886(etrx2)*	Km^r, Erm^r	This work
PN331	D39*lux*Δ*spd_0572*Δ*spd_0886*	Km^r, Erm^r, Spec^r	This work
PN367	D39Δ*cps*Δ*spd_0573(msrAB2)*	Km^r, Erm^r	This work
PN384	D39Δ*cps*Δ*spd_1193(msrAB1)*	Km^r, Erm^r	This work
PN368	D39*lux*Δ*spd_0573(msrAB2)*	Km^r, Erm^r	This work
PN386	D39*lux*Δ*spd_1193(msrAB1)*	Km^r, Spec^r	This work
PN387	D39*lux*Δ*spd_0573*Δ*spd_1193*	Km^r, Erm^r, Spec^r	This work
PN453	D39Δ*cps*Δ*spd_0571(ccdA1)*	Km^r, Erm^r	This work
PN454	D39Δ*cps*Δ*spd_0885(ccdA2)*	Km^r, Spec^r	This work
PN455	D39Δ*cps*Δ*spd_0571*Δ*spd_0885*	Km^r, Erm^r, Spec^r	This work
Escherichia coli			
DH5α	*Δ(lac)U169, endA1, gyrA46, hsdR17, Φ80Δ(lacZ)M15, recA1, relA1, supE44, thi-1*	None	Novagen
BL21 (DE3)	*E.coli B, F- dcm ompT hsdS gal λ (DE3), T7 polymerase gene under control of the lacUV5 promoter*	None	Stratagene
Plasmids			
pGEM®-T Easy	TA cloning vector for PCR products; Ap^r	Ap^r	Promega
pE89	pCR2.1Topo with erythromycin (*ermB*) cassette	Ap^r, Km^r, Erm^r	(Hammerschmidt et al, 2000)
pE96	pGEM-T with spectinomycin (*aad9*) cassette	Ap^r, Spec^r	(Pracht et al, 2005)
p561	pGEM-T derivative with *sp_0659* (*etrx1*) 5´and 3´ flanking region for mutagenesis	Ap^r	This work
p567	pGEM-T derivative with *sp_0659* (*etrx1*) interrupted by *ermB* resistance gene cassette	Ap^r, Erm^r	This work
p601	pGEM-T derivative with *sp_0659* (*etrx1*) interrupted by *aad9* resistance gene	Ap^r, Spec^r	This work
p575	pGEM-T derivative with *sp_1000* (*etrx2*) 5´and 3´ flanking region for mutagenesis	Ap^r	This work
p645	pGEM-T derivative with *sp_1000* (*etrx2*) interrupted by *ermB* gene resistance cassette	Ap^r, Erm^r	This work
p735	pGEM-T derivative with *sp_0660* (*msrAB2*) 5´and 3´ flanking region for mutagenesis	Ap^r	This work
p737	pGEM-T derivative with *sp_0660* (*msrAB2*) interrupted by *ermB* gene resistance cassette	Ap^r, Erm^r	This work
p742	pGEM-T derivative with *sp_1359* (*msrAB1*) 5´and 3´ flanking region for mutagenesis	Ap^r	This work
P748	pGEM-T derivative with *sp_1359* (*msrAB1*) interrupted by *aad9* gene resistance cassette	Ap^r, Spec^r	This work
p894	pGEM-T derivative with *sp_0658* (*ccdA1*) 5´and 3´ flanking region for mutagenesis	Ap^r	This work
P899	pGEM-T derivative with *sp_0658* (*ccdA1*) interrupted by *ermB* gene resistance cassette	Ap^r, Erm^r	This work
p895	pGEM-T derivative with *sp_0999* (*ccdA2*) 5´and 3´ flanking region for mutagenesis	Ap^r	This work

Table S1: *continuing*

Strain or plasmid	Serotype and relevant Genotype[a]	Resistance	Source or Reference
p900	pGEM-T derivative with *sp_0999* (*ccdA2*) interrupted by *aad9* gene resistance cassette	Ap^r, $Spec^r$	This work
pET28	Protein expression vector	Km^r	Novagen
pTP1	pET28 expression vector with a TEV protease cleavage site	Km^r, Erm^r	This work
p629	pTP1 with TIGR4 *sp_0659* (*etrx1*) for protein production and mice immunization	Km^r	This work
p651	pTP1 with TIGR4 *sp_1000* (*etrx2a*) for protein production and mice immunization	Km^r	This work
p807	pTP1 with TIGR4 *sp_1000* (*etrx2b*) for protein production and mice immunization	Km^r	This work
p792	pTP1 with TIGR4 *sp_0660* (*msrAB2*) for protein production and mice immunization	Km^r	This work
p888	pTP1 with *msrA2* for protein production	Km^r	This work
p891	pTP1 with *msrB2* for protein production	Km^r	This work

[a]Ap, ampicillin; Km, kanamycin; Erm, erythromycin; Spec, spectinomycin; r, resistant; [b]MUD, Medical University of Düsseldorf, Germany

Table S2. Primer list

Primer intended use	Primer	Sequence (5'-3')[a]
Insertion-deletion mutagenesis		
Amplification of *sp_0659* + 5´ and 3´ flanking region	etrx1_402	5`-CTACTACTAGAATTCGGATGGTGCTCAGGCTTC-3`
	etrx1_405	5`-AACCTTCCAAGCTGCAGAGGATAGGCCGCCTGATTA-3`
Inverse PCR of *sp_0659* + 5´ and 3´ flanking region (pGEM-T Easy)	etrx1_404	5`-ACTCACTCACTGAAGCTTGCCTAGGAGGCGTCTTATGAA-3`
	etrx1_403	5`-ATCATCATCATCGAAGCTTGCGAACCTGCTCCAAGAA-3`
Amplification of *sp_1000* + 5´ and 3´ flanking region	etrx2_423	5`-CTACTAGAATTCTCCTCTTCTGCCAGTCTATGC-3`
	etrx2_426	5`-CCAAGCTGCAGCCAACCAGGCAGGGAAT-3`
Inverse PCR of *sp_1000* + 5´ and 3´ flanking region (pGEM-T Easy)	etrx2_425	5`-CTCACTGCTCGAGCGGATGCAGAAGCAGCA-3`
	etrx2_424	5`-ATCATCGGTACCTGACAAGAGACTTAAGCCAGCA-3`
Amplification of *sp_0660* + 5´ and 3´ flanking region	msrAB2_700	5`-GGCAAACATGTGTTCTTGGAG-3`
	msrAB2_701	5`-AAAGGCAAGCCCAACTCTTTC-3`
Inverse PCR of *sp_0660* + 5´ and 3´ flanking region (pGEM-T Easy)	msrAB2_695	5`-CTCACTGAAGCTTATTCCCAAAGACCAAATGGA-3`
	msrAB2_694	5`-ATCATCGAAGCTTACTCCTAGCAACAAGAAG-3`
Amplification of *sp_1359* + 5´ and 3´ flanking region	msrAB1_769	5`-CTGGGAATTCTTGAAGGGCAAGTCTCTGCT-3`
	msrAB1_772	5`-CAACTGCAGTTTCTTGACCAAAAGCACGA-3`
Inverse PCR of *sp_1359* + 5´ and 3´ flanking region (pGEM-T Easy)	msrAB1_771	5`-CTCACTGAAGCTTAAACAGAGAGTGGGGCTTCC-3`
	msrAB1_770	5`-ATCATCGAAGCTTATTCCTCTAGGCCCCAAAAA-3`
Amplification of *sp_0658* + 5´ and 3´ flanking region	ccdA1_905	5`-CTGGGAATTCTTTACAAGGGTTGGCAGGTC-3`
	ccdA1_908	5`-CAACTGCAGTCACTGCTTTTCCTCCTTGG-3`
Inverse PCR of *sp_0658* + 5´ and 3´ flanking region (pGEM-T Easy)	ccdA1_907	5`-CTCACTGAAGCTTTGGGCTTCTTGGTTCTGTTT-3`
	ccdA1_906	5`-ATCGAAGCTTAAGGATAGAATCCCTGCCAAA-3`
Amplification of *sp_0998* + 5´ and 3´ flanking region	ccdA2_909	5`-CTGGGAATTCGCAGGAGTGATTGTGGTCAG-3`
	ccdA2_912	5`-CAACTGCAGGGCTATTTGTCGTGCACTT-3`
Inverse PCR of *sp_0998* + 5´ and 3´ flanking region (pGEM-T Easy)	ccdA2_911	5`-CTCACTGGATCCTGGTGGTTTCCTCATTGTTTT-3`
	ccdA2_910	5`-ATCATCGCTAGCCAGCATAGACTGGCAGA-3`
Antibiotic cassette amplification		
erythromycin (*ermB*)	ermB_105	5`-GATGATGATGATCCCGGGTACCAAGCTTGAATTCACGGTTCGT GTTCGTGCTG-3`
	ermB_106	5`-AGTGAGTGAGTCCCGGGCTCGAGAAGCTTGA ATTCGTAGGCGCTAGGGACCTC-3`
spectimomycin (*aad9*)	aad9_117	5`-AAAAAGCTTGAATTCGGATCCATCGATTTTCGTTCGTGAATAC-3`
	aad9_118	5`-AAAAAGCTTGCTAGCAATTAGAATGAATATTTCCC-3`
Recombinant protein production		
sp_0659 (TIGR4; etrx1)	etrx1_447	5`-AAGCGCTAGCTCAGGCAAGTCCGTGACTAG-3`
	etrx1_448	5`-GGCCAAGCTTAGGCTAATTCCTTCAAAGTTTG-3`
sp_1000 (TIGR4; *etrx2a*)	etrx2_486	5`-AAACGCTAGCGGTGAGGAAGAAACTAAAAAG-3`
	etrx2_487	5`-ACGCGAGCTCCTAGTTCATTTCTTTAAATGC-3`
sp_1000 (TIGR4; *etrx2b*)	etrx2_789	5`-AAGGGCTAGCGCTGTTGGAAAAGATGCT-3`
	etrx2_487	5`-ACGCGAGCTCCTAGTTCATTTCTTTAAATGC-3`
sp_0660 (TIGR4; *msrAB2*)	msrAB2_863	5`-ATATGCTAGCCACGAAATTTATCTAGCTGG-3`
	msrAB2_692	5`-CCCGGGCCGAGCTCTTAATCAACATAATCTAG-3`
sp_0660 (TIGR4; *msrA2*)	msrAB2_863	5`-ATATGCTAGCCACGAAATTTATCTAGCTGG-3`
	msrA2_1051	5`-GGCCGAGCTCTTAATAGGCCGCCTGATTAAC-3`
sp_0660 (TIGR4; *msrB2*)	msrB2_1052	5`-GCATGCTAGCAGTGATGAGGAATTGAAAAA-3`
	msrAB2_692	5`-CCCGGGCCGAGCTCTTAATCAACATAATCTAG-3`
RT-PCR		
spd_0571 (D39)	ccdA1_708	5`-GGATGGTGCTCAGGCTTCTA-3`
	ccdA1_709	5`-ATCTCCATTTGGTGCAAACC-3`
spd_0572 (D39)	etrx1_710	5`-TGGGAGTAGATGGCAAGACC-3`
	etrx1_711	5`-GTCCGCTTCAGATTGCTCTC-3`
spd_0573 (D39)	msrAB2_712	5`-TTAGCCAAGGAGGAAAAGCA-3`
	msrAB2_713	5`-TGGCATCATAGGTGACATGG-3`
spd_0885 (D39)	ccdA2_775	5`-TGCCAGTCTATGCTGGGATT-3`

Table S2: *continuing*

Primer intended use	Primer	Sequence (5’-3’)
spd_0886 (D39)	ccdA2_776	5`-CCCATGACATATCGAAACCA-3`
	etrx2_777	5`-GCCTGTGGTGAGGAAGAAAC-3`
	etrx2_778	5`-CACCATGAAGCCCAAAACTT-3`

[a]Restriction sites are underlined
