# Supplementary figures and images for: Molecular architecture of Streptococcus pneumoniae surface thioredoxin-fold lipoproteins crucial for extracellular oxidative stress resistance and maintenance of virulence
Source: EMBO Mol Med. 2013 Oct 18;5(12):1852–70. doi: 10.1002/emmm.201202435 (PMC3914529; doi:10.1002/emmm.201202435)

Figure 1D

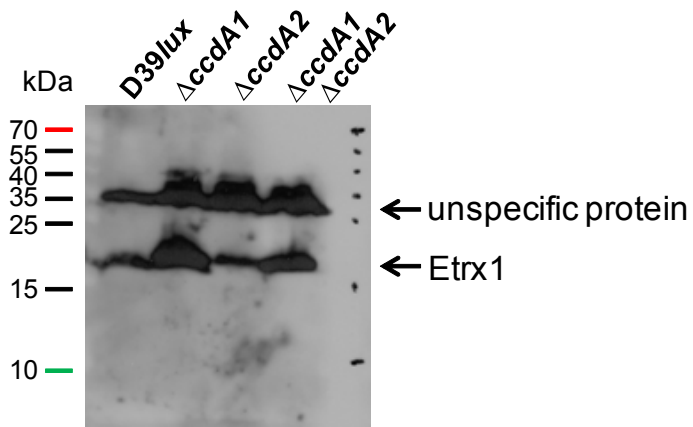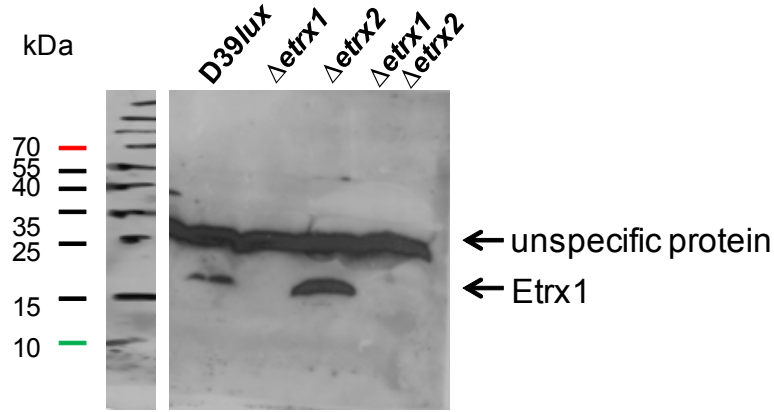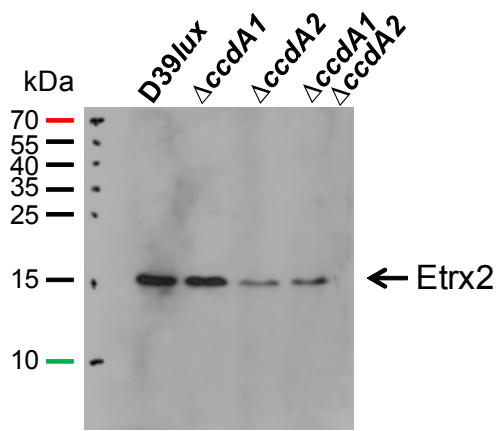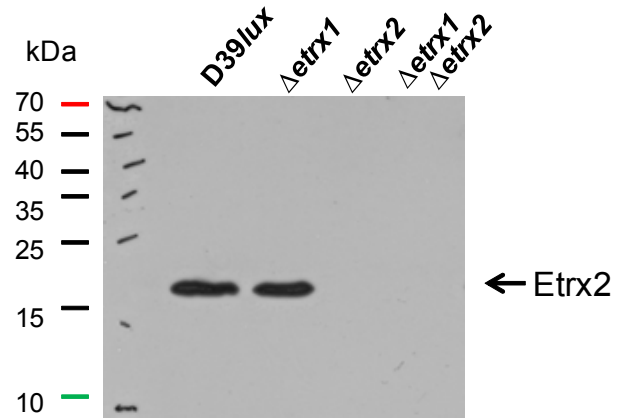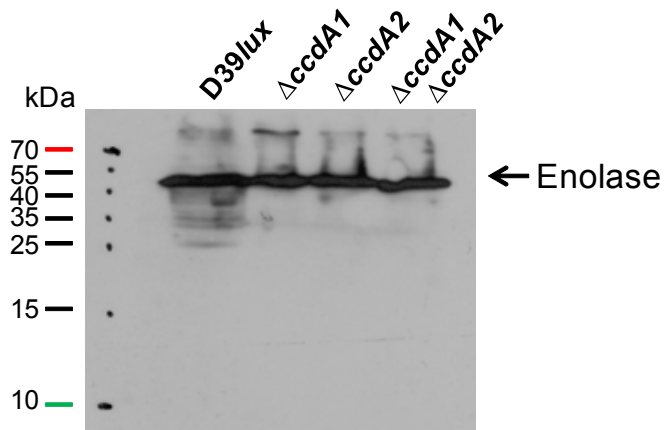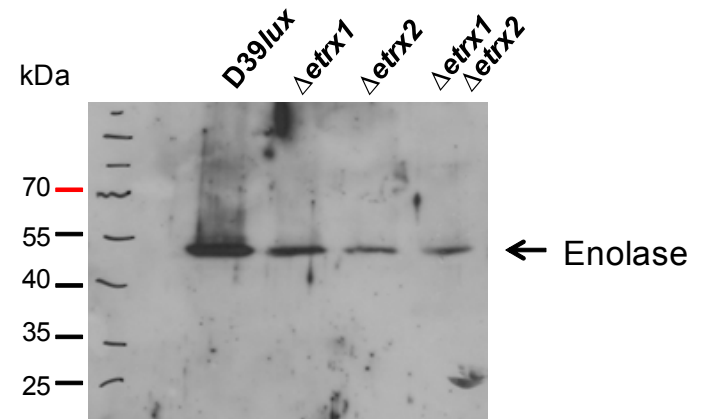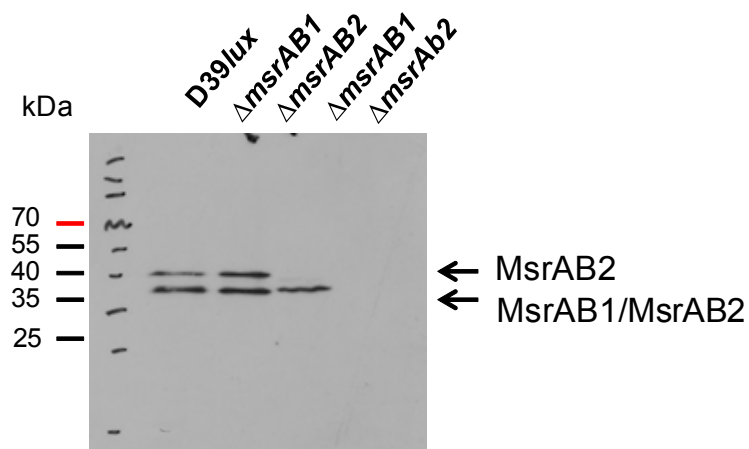

# Figure 2B

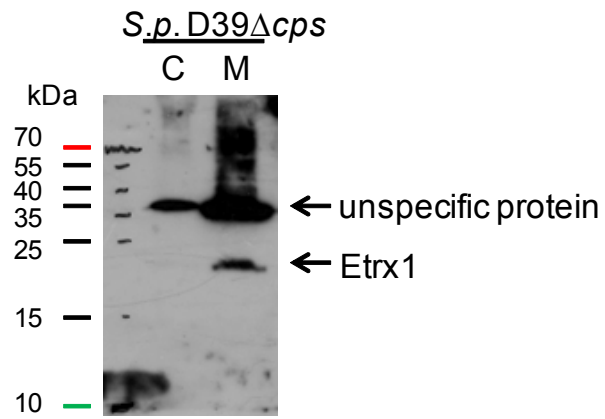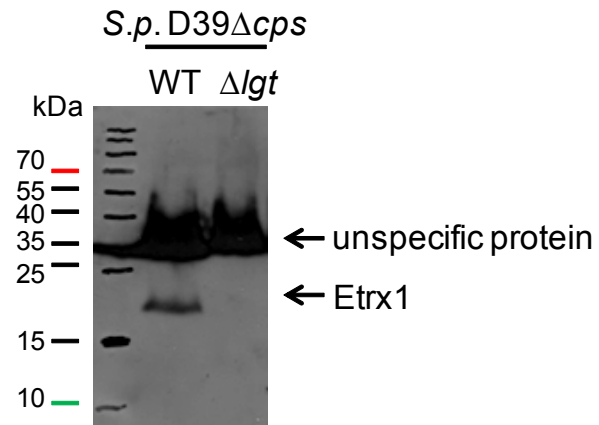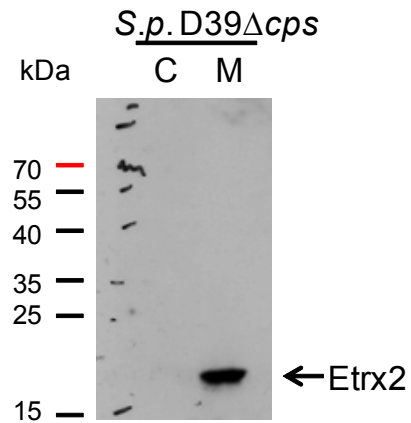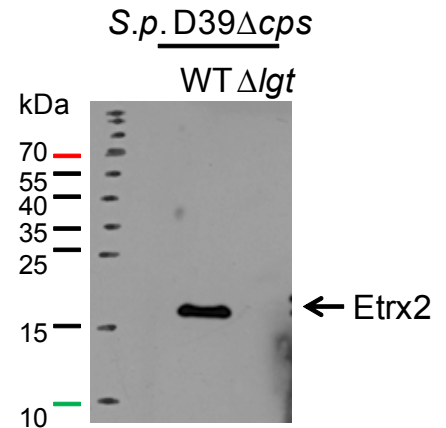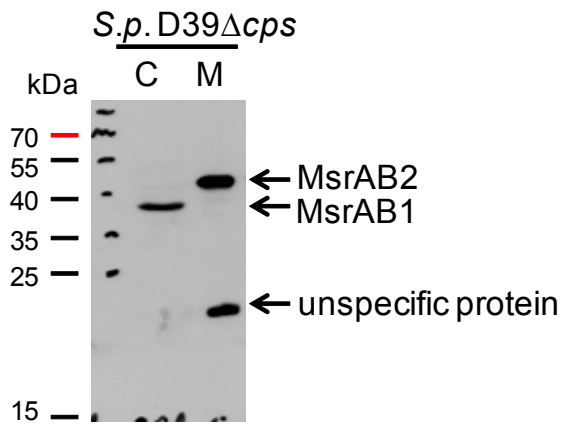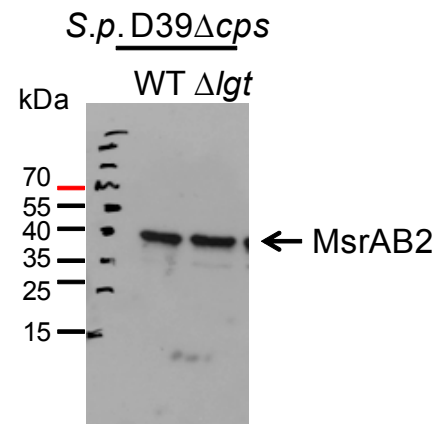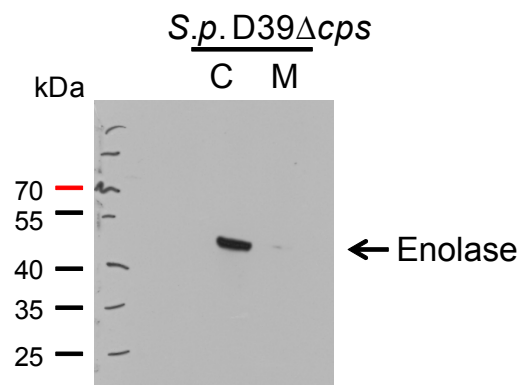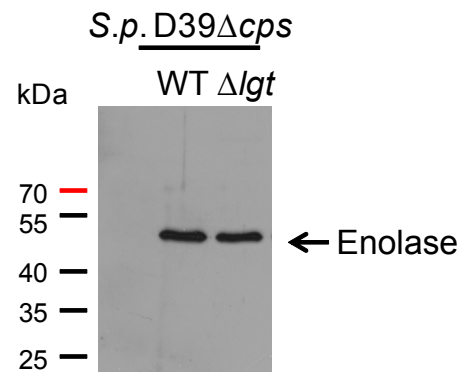

Figure 5A

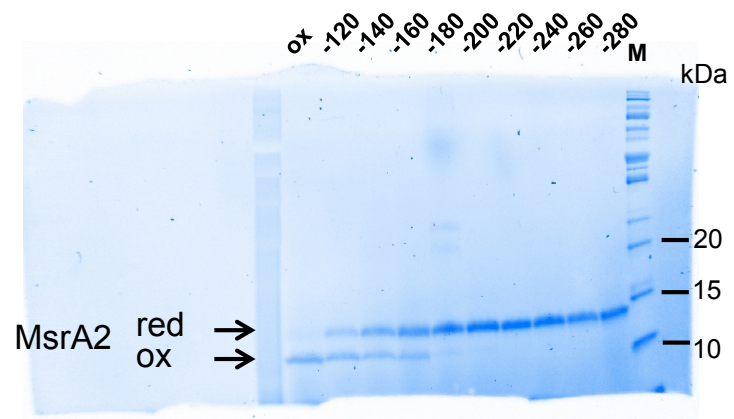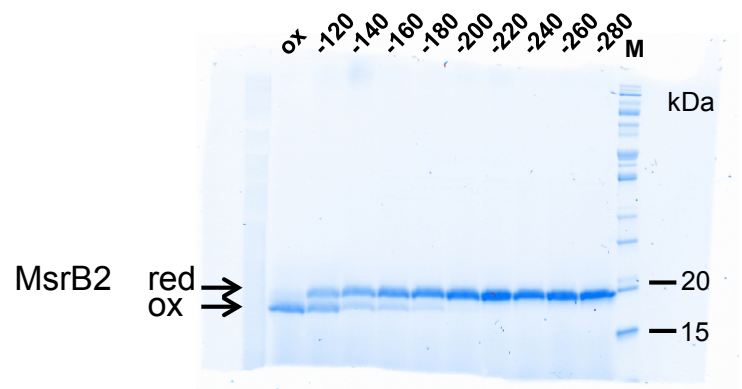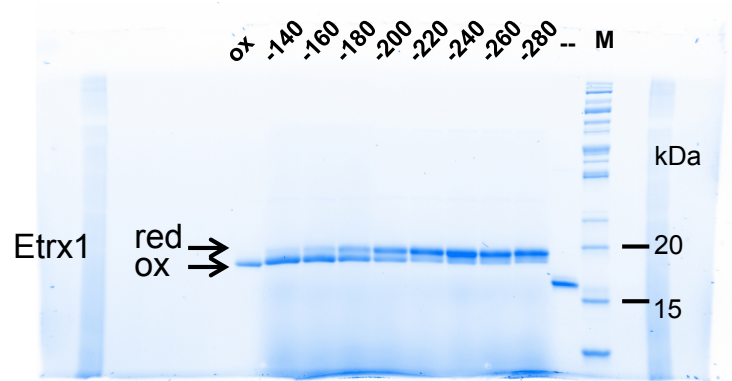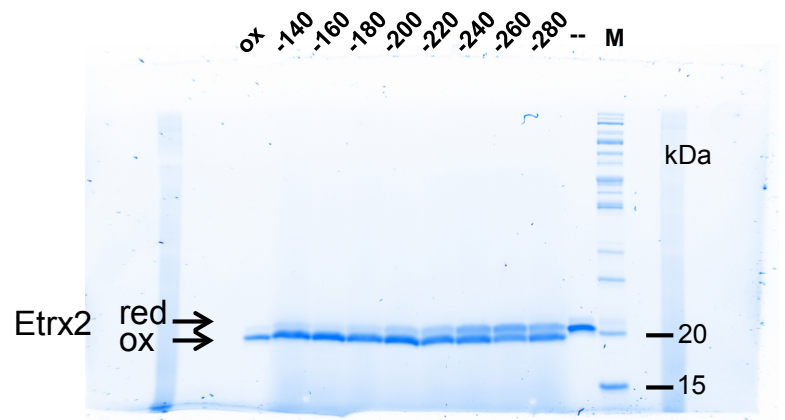

Figure 5C

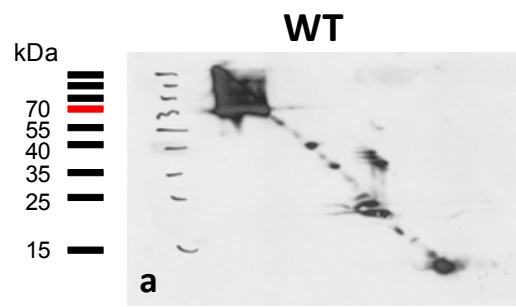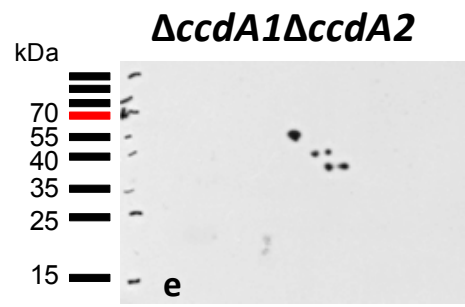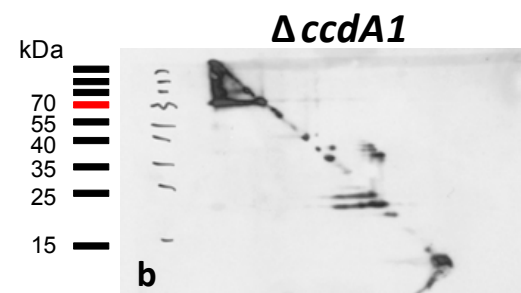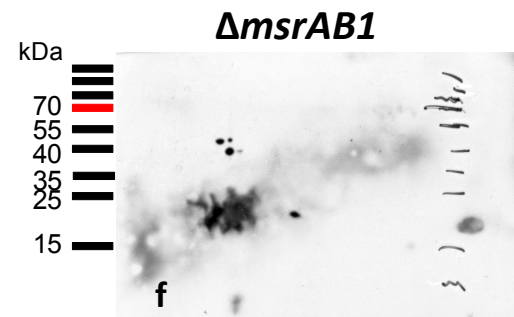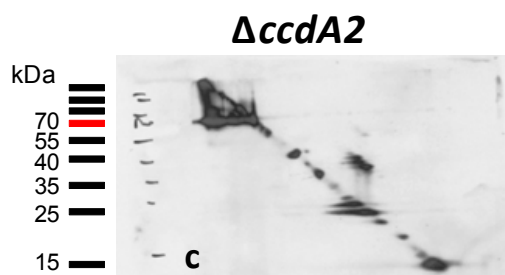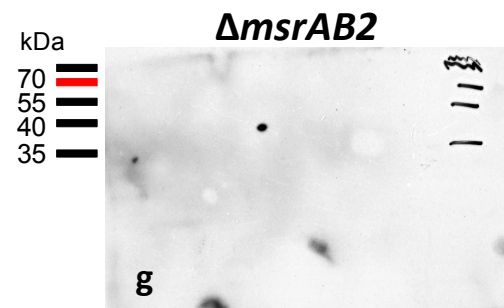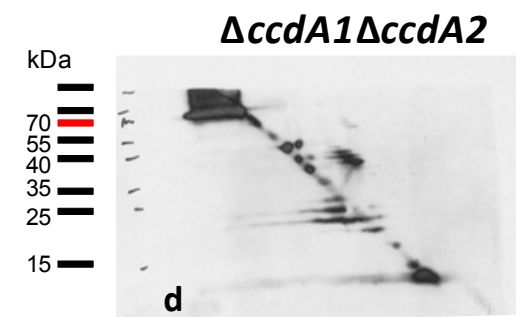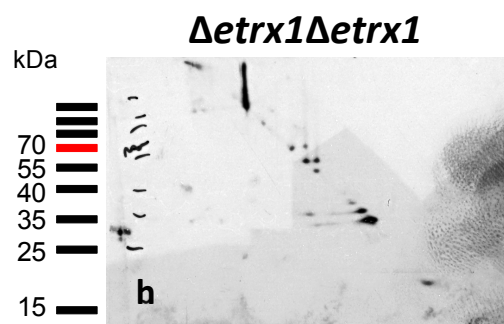

Supplement: Supplementary file 3 [file emmm0005-1852-sd3.pdf]
